# Supplementary material for: 4Ms for Early Learners: A Skills-Based Geriatrics Curriculum for Second-Year Medical Students
Source: MedEdPORTAL. 2022 Jun 28;18:11264. doi: 10.15766/mep_2374-8265.11264 (PMC9237204; doi:10.15766/mep_2374-8265.11264)
Supplement: Supplementary file 1 — The 4Ms Approach.pptxFaculty Guide.docxStudent A Handout.docxStudent B Handout.docxStudent C Handout.docxPre- and Postsession Student Surveys.docxLarge-Group Session Evaluation Form.docxGeriatrics SP Case.docxGeriatrics SP Checklist.docx [file mep_2374-8265.11264-s001.zip › A. The 4Ms Approach.pptx]

## Slide 1
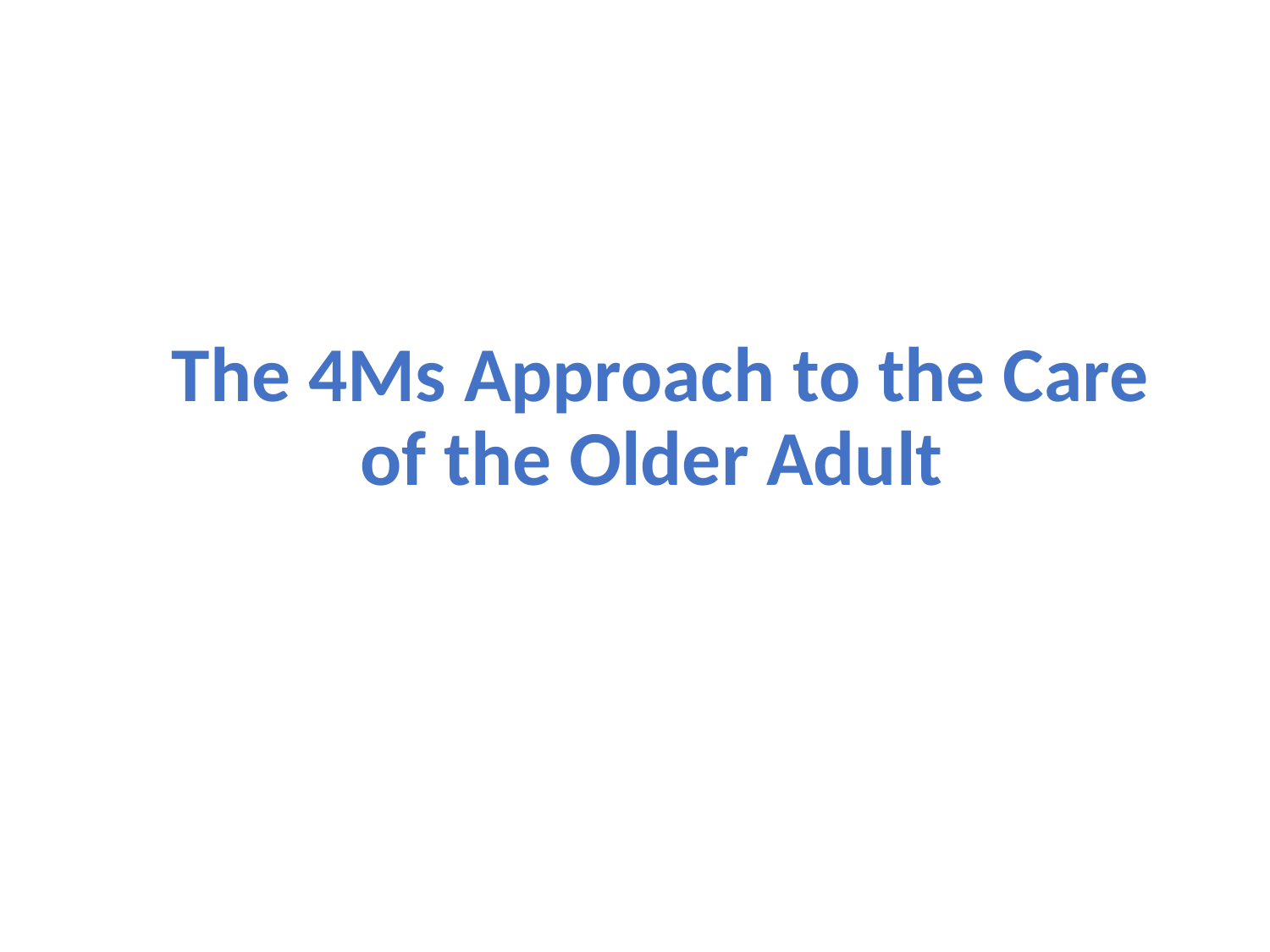

The 4Ms Approach to the Care of the Older Adult

## Slide 2
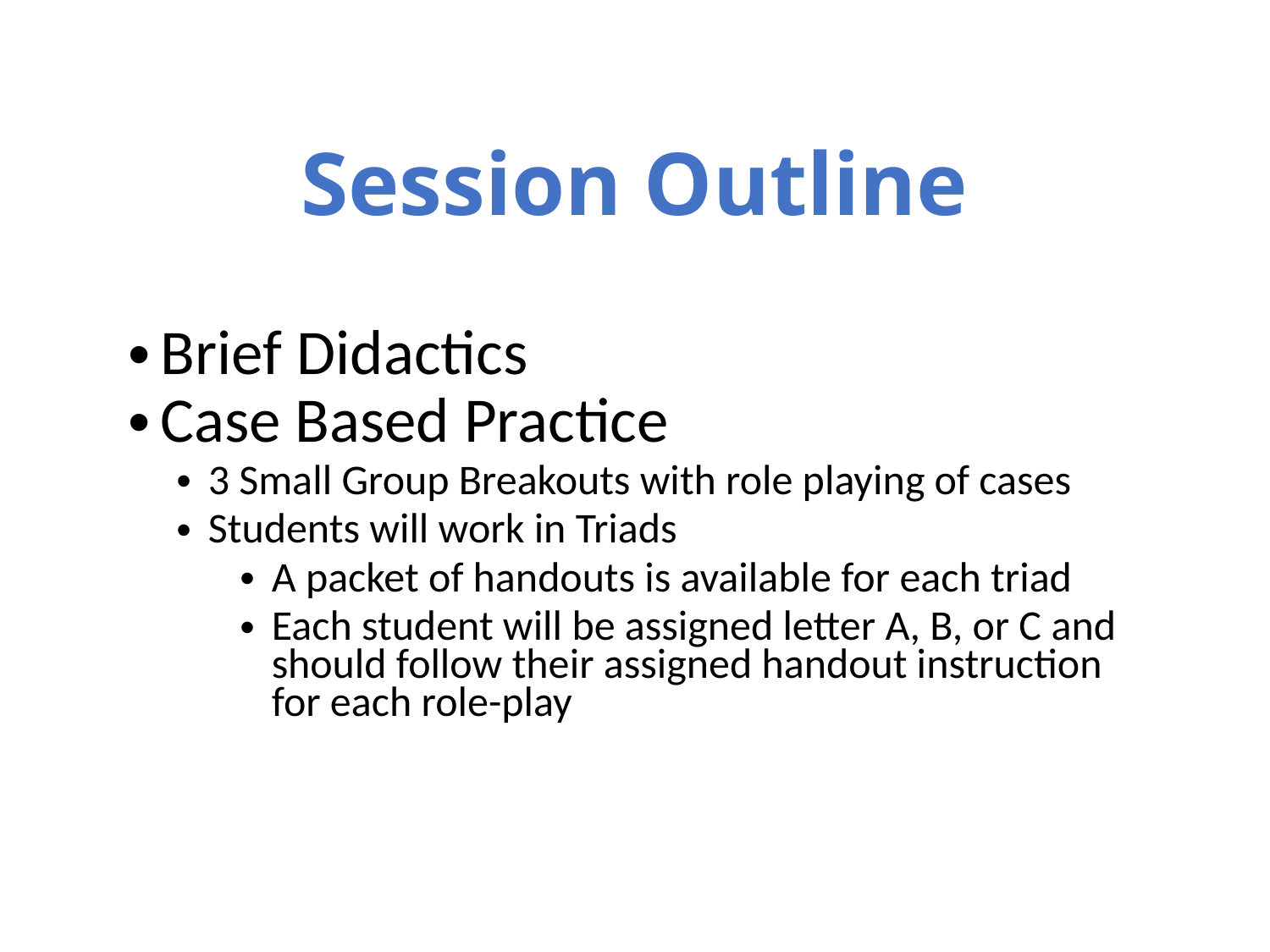

Session Outline
Brief Didactics
Case Based Practice
3 Small Group Breakouts with role playing of cases
Students will work in Triads
A packet of handouts is available for each triad
Each student will be assigned letter A, B, or C and should follow their assigned handout instruction for each role-play

## Slide 3
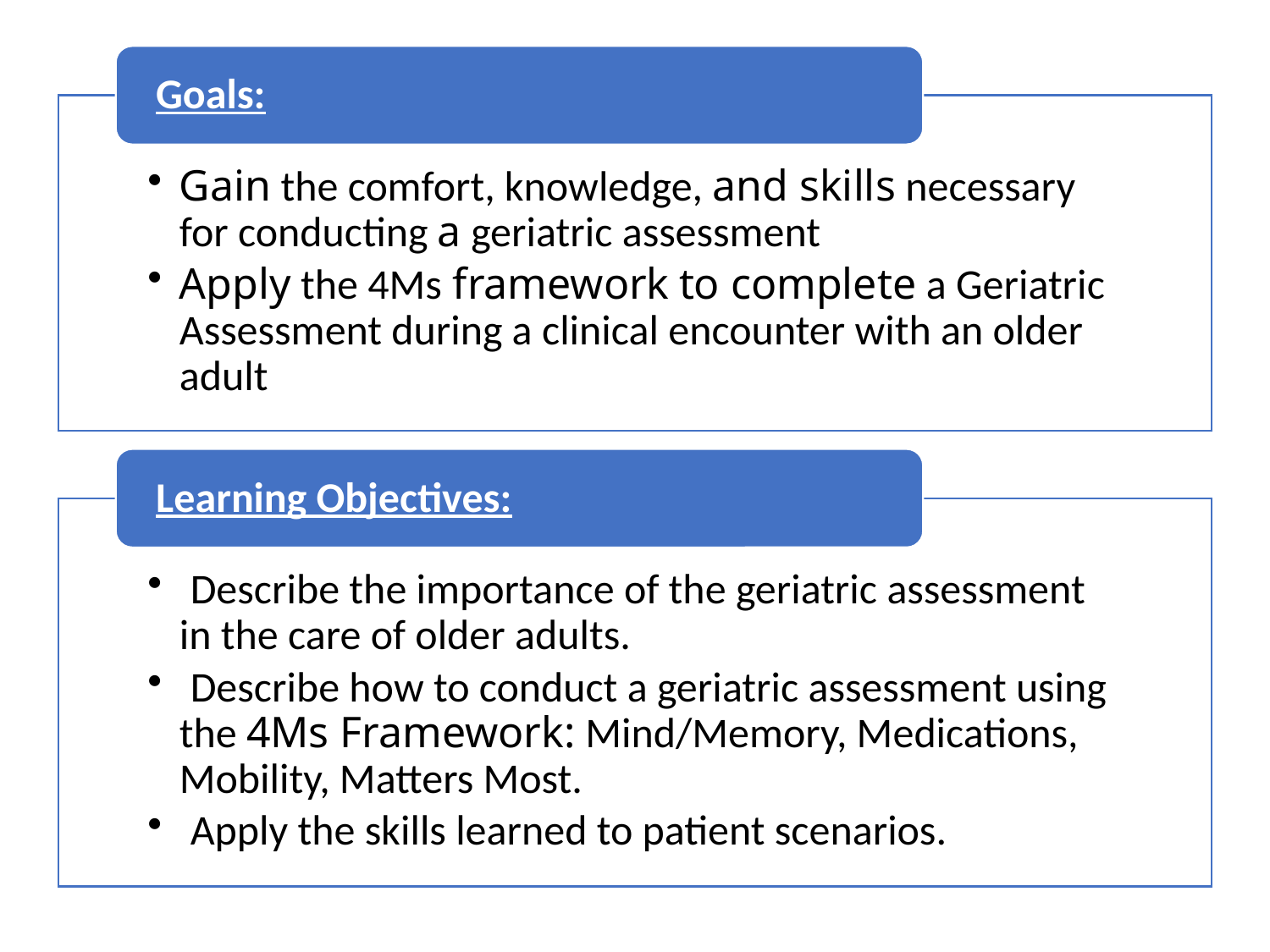

## Slide 4
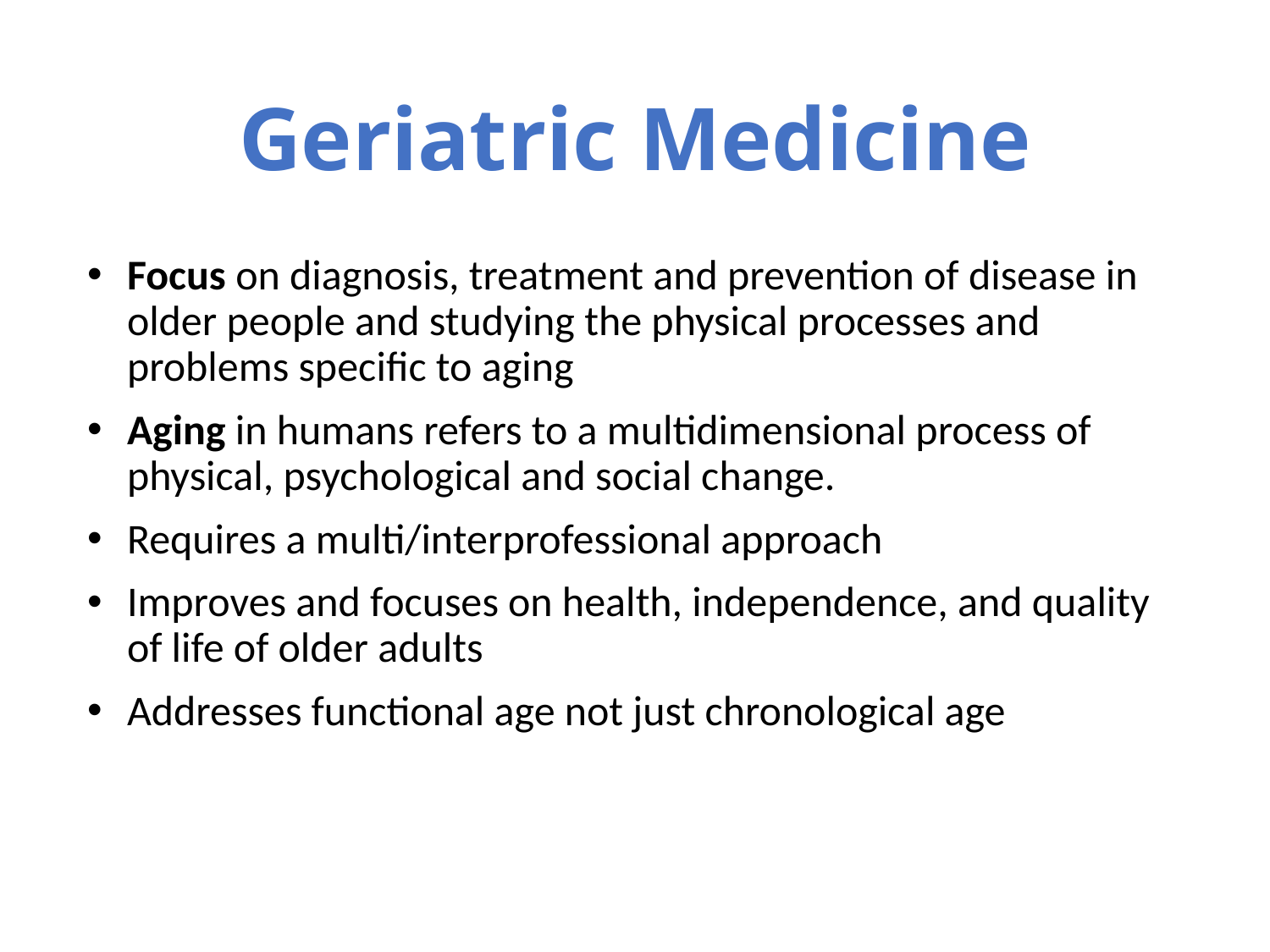

# Geriatric Medicine
Focus on diagnosis, treatment and prevention of disease in older people and studying the physical processes and problems specific to aging
Aging in humans refers to a multidimensional process of physical, psychological and social change.
Requires a multi/interprofessional approach
Improves and focuses on health, independence, and quality of life of older adults
Addresses functional age not just chronological age

## Slide 5
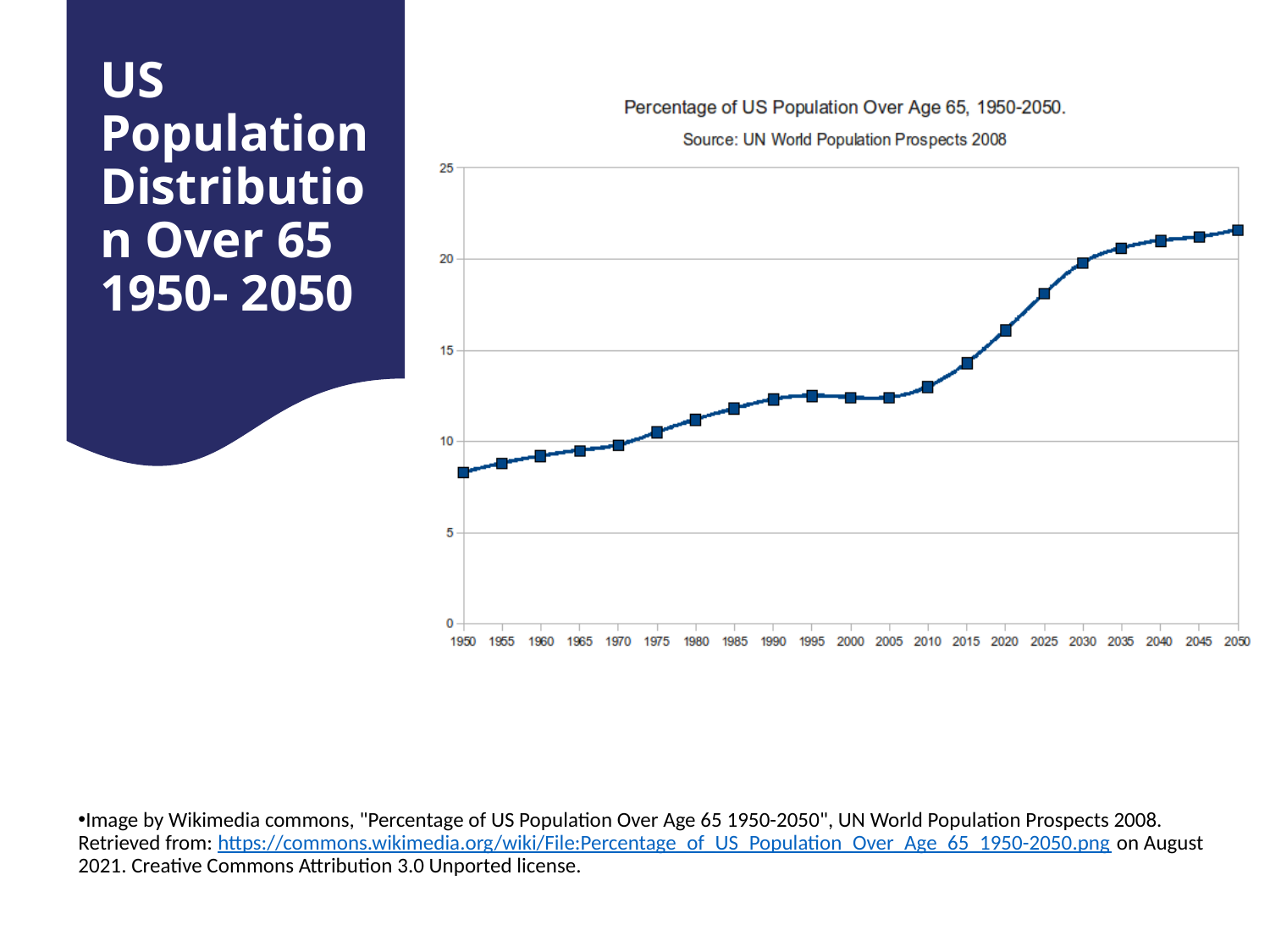

# US Population Distribution Over 65  1950- 2050
Image by Wikimedia commons, "Percentage of US Population Over Age 65 1950-2050", UN World Population Prospects 2008. Retrieved from: https://commons.wikimedia.org/wiki/File:Percentage_of_US_Population_Over_Age_65_1950-2050.png on August 2021. Creative Commons Attribution 3.0 Unported license.

## Slide 6
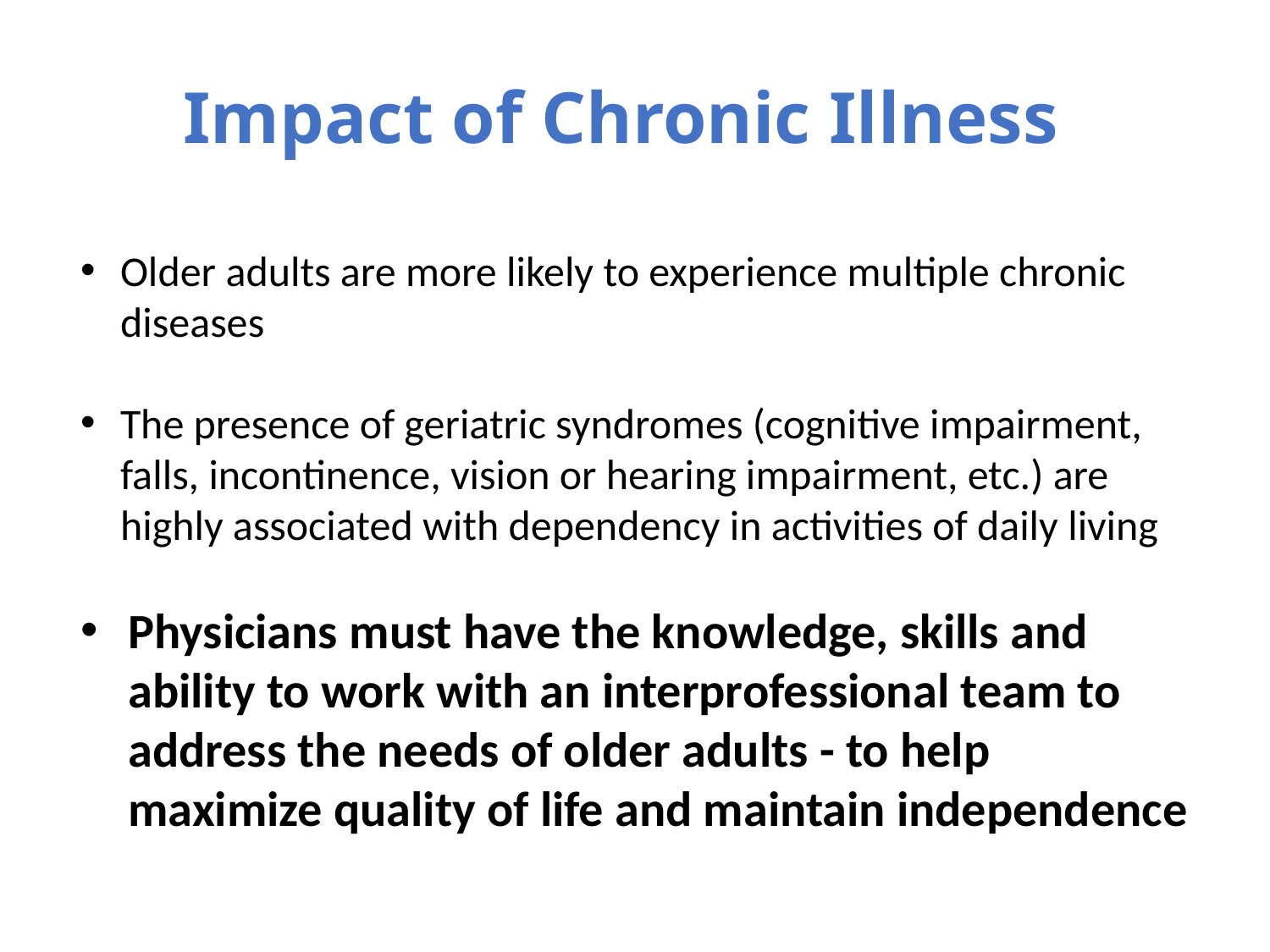

# Impact of Chronic Illness
Older adults are more likely to experience multiple chronic diseases
The presence of geriatric syndromes (cognitive impairment, falls, incontinence, vision or hearing impairment, etc.) are highly associated with dependency in activities of daily living
Physicians must have the knowledge, skills and ability to work with an interprofessional team to address the needs of older adults - to help maximize quality of life and maintain independence

## Slide 7
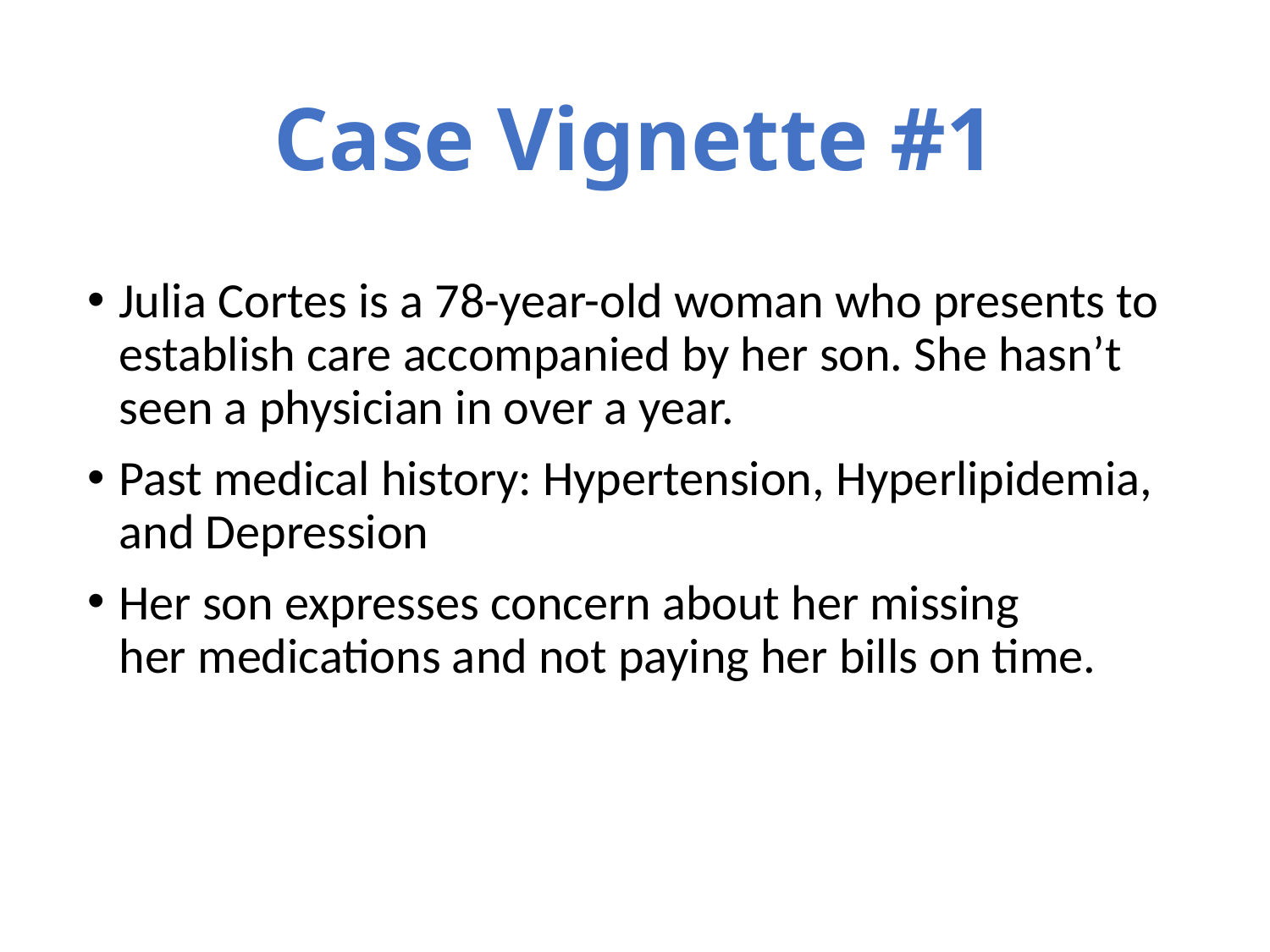

# Case Vignette #1
Julia Cortes is a 78-year-old woman who presents to establish care accompanied by her son. She hasn’t seen a physician in over a year.
Past medical history: Hypertension, Hyperlipidemia, and Depression
Her son expresses concern about her missing her medications and not paying her bills on time.

## Slide 8
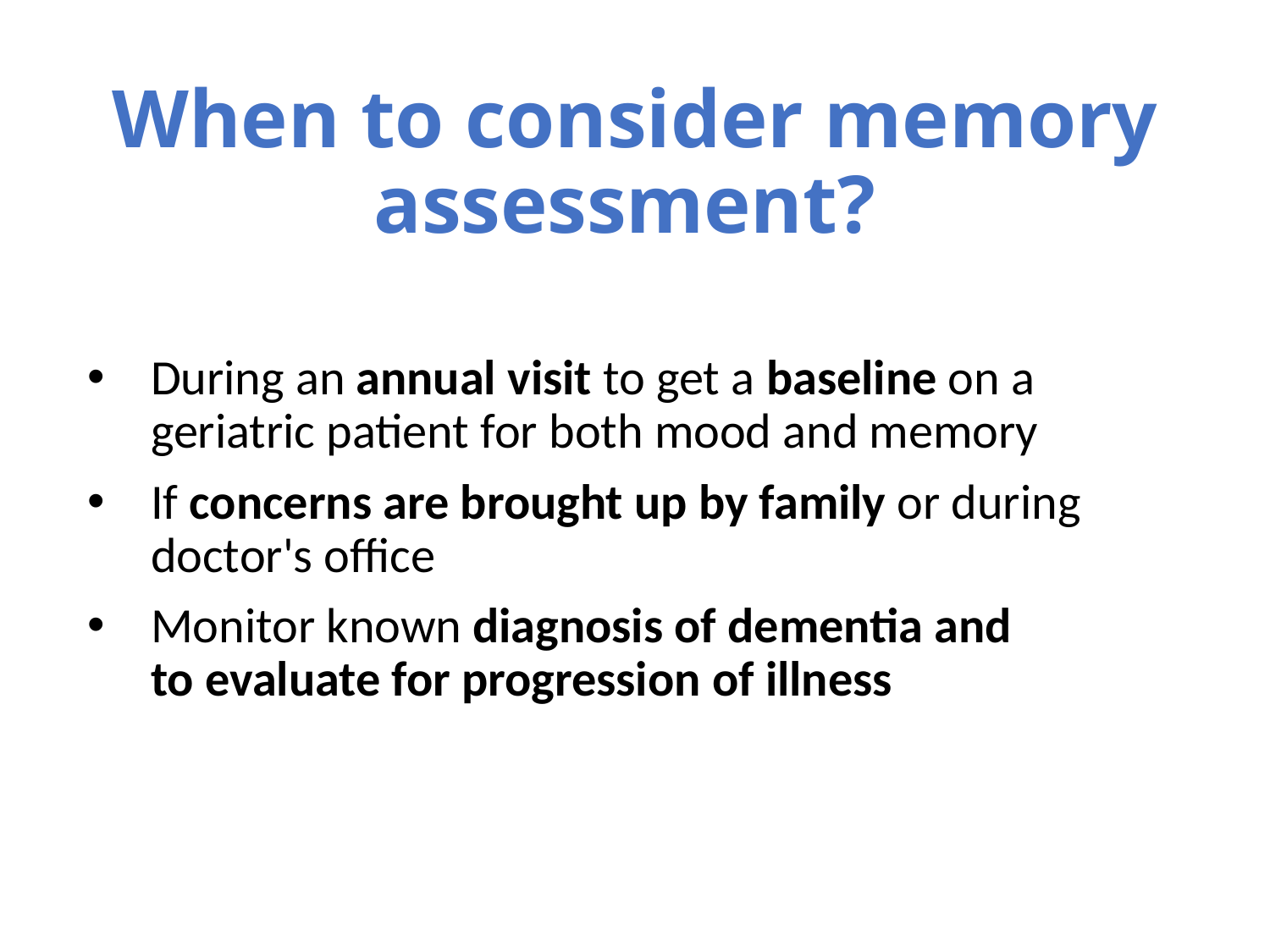

# When to consider memory assessment?
During an annual visit to get a baseline on a geriatric patient for both mood and memory
If concerns are brought up by family or during doctor's office
Monitor known diagnosis of dementia and to evaluate for progression of illness

## Slide 9
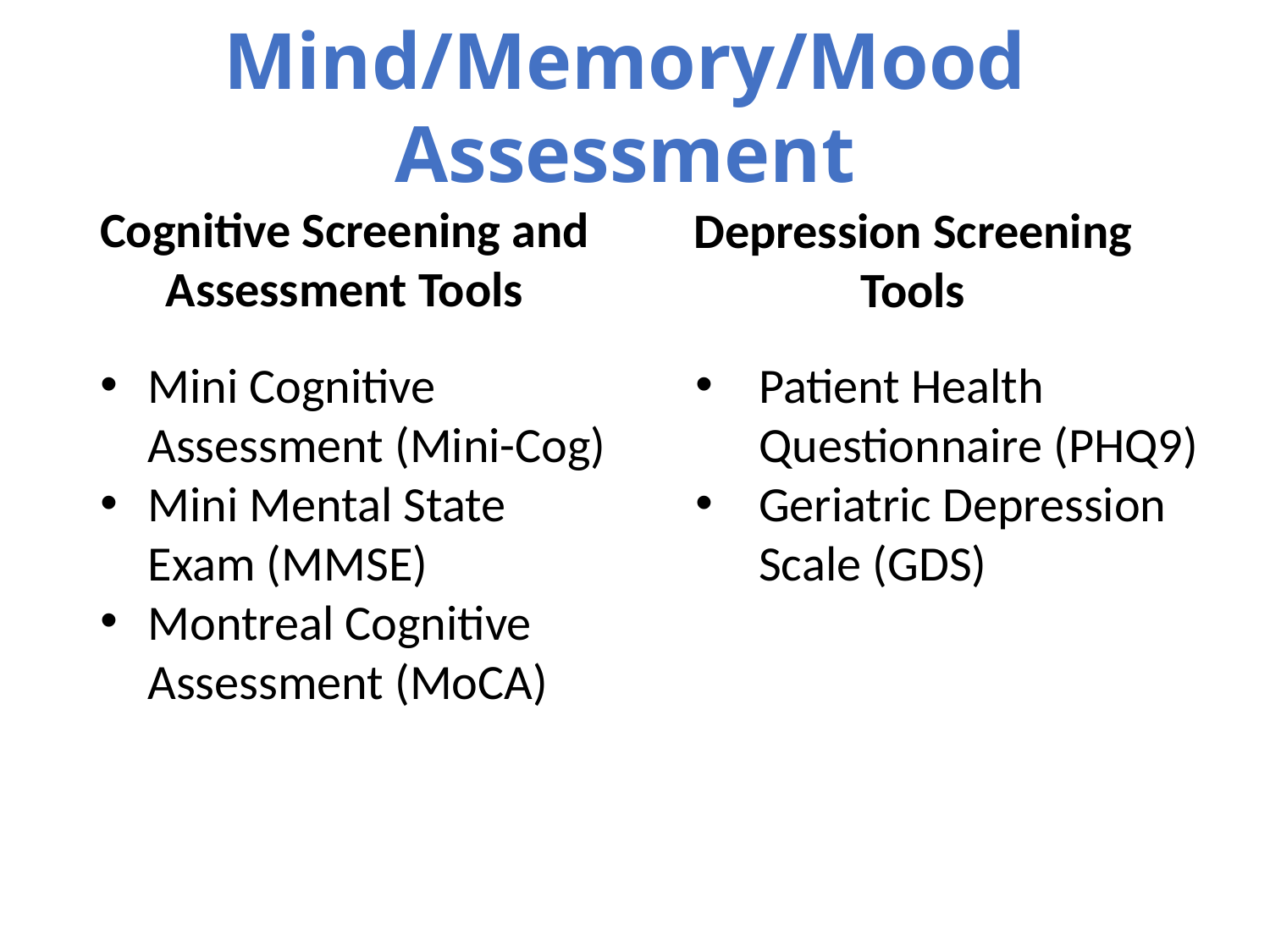

# Mind/Memory/Mood Assessment
Cognitive Screening and Assessment Tools
Depression Screening Tools
Mini Cognitive Assessment (Mini-Cog)
Mini Mental State Exam (MMSE)
Montreal Cognitive Assessment (MoCA)
Patient Health Questionnaire (PHQ9)
Geriatric Depression Scale (GDS)

## Slide 10
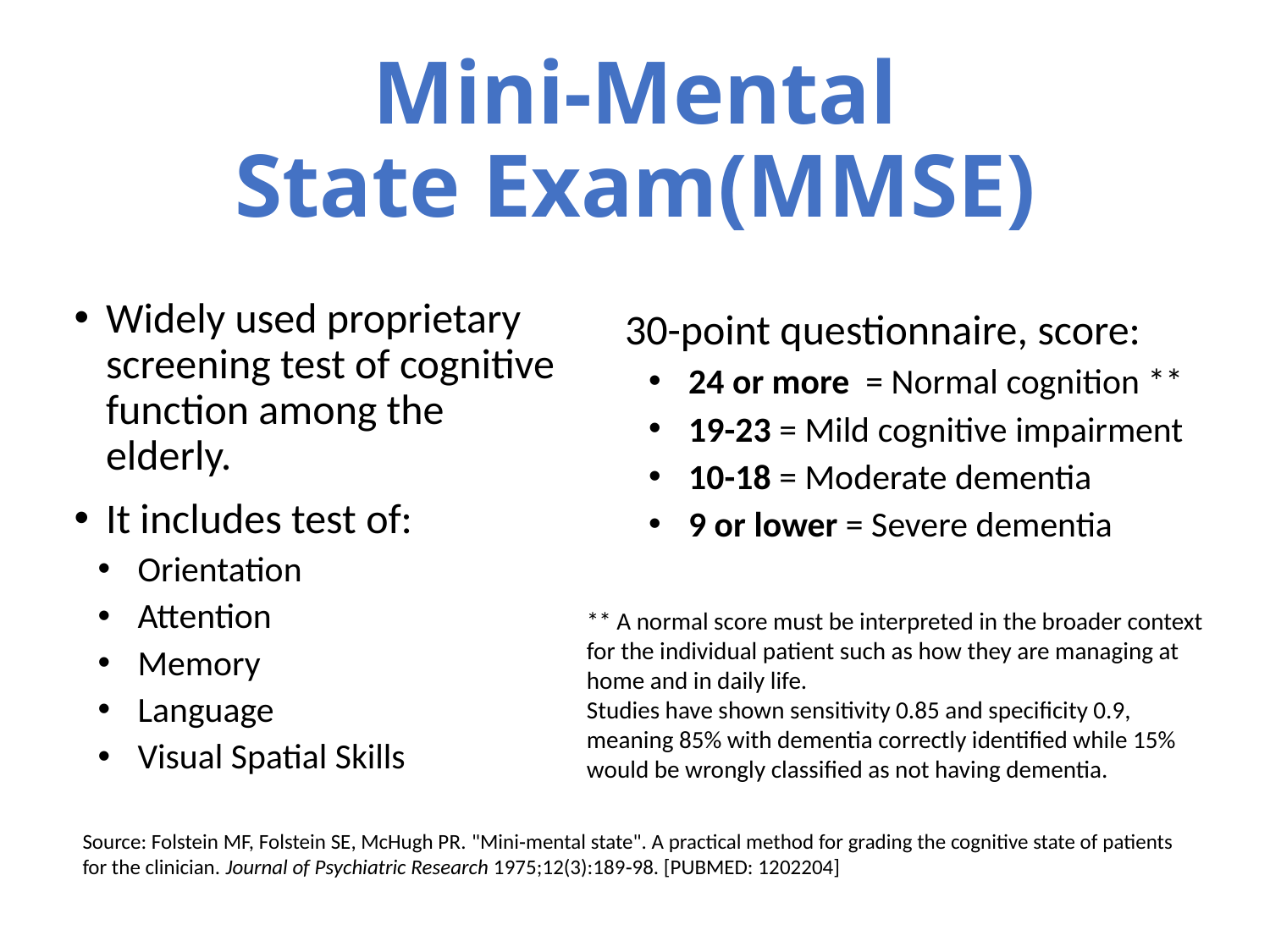

# Mini-Mental State Exam(MMSE)
Widely used proprietary screening test of cognitive function among the elderly.
It includes test of:
Orientation
Attention
Memory
Language
Visual Spatial Skills
30-point questionnaire, score:
24 or more  = Normal cognition **
19-23 = Mild cognitive impairment
10-18 = Moderate dementia
9 or lower = Severe dementia
** A normal score must be interpreted in the broader context for the individual patient such as how they are managing at home and in daily life.
Studies have shown sensitivity 0.85 and specificity 0.9, meaning 85% with dementia correctly identified while 15% would be wrongly classified as not having dementia.
Source: Folstein MF, Folstein SE, McHugh PR. "Mini‐mental state". A practical method for grading the cognitive state of patients for the clinician. Journal of Psychiatric Research 1975;12(3):189‐98. [PUBMED: 1202204]

## Slide 11
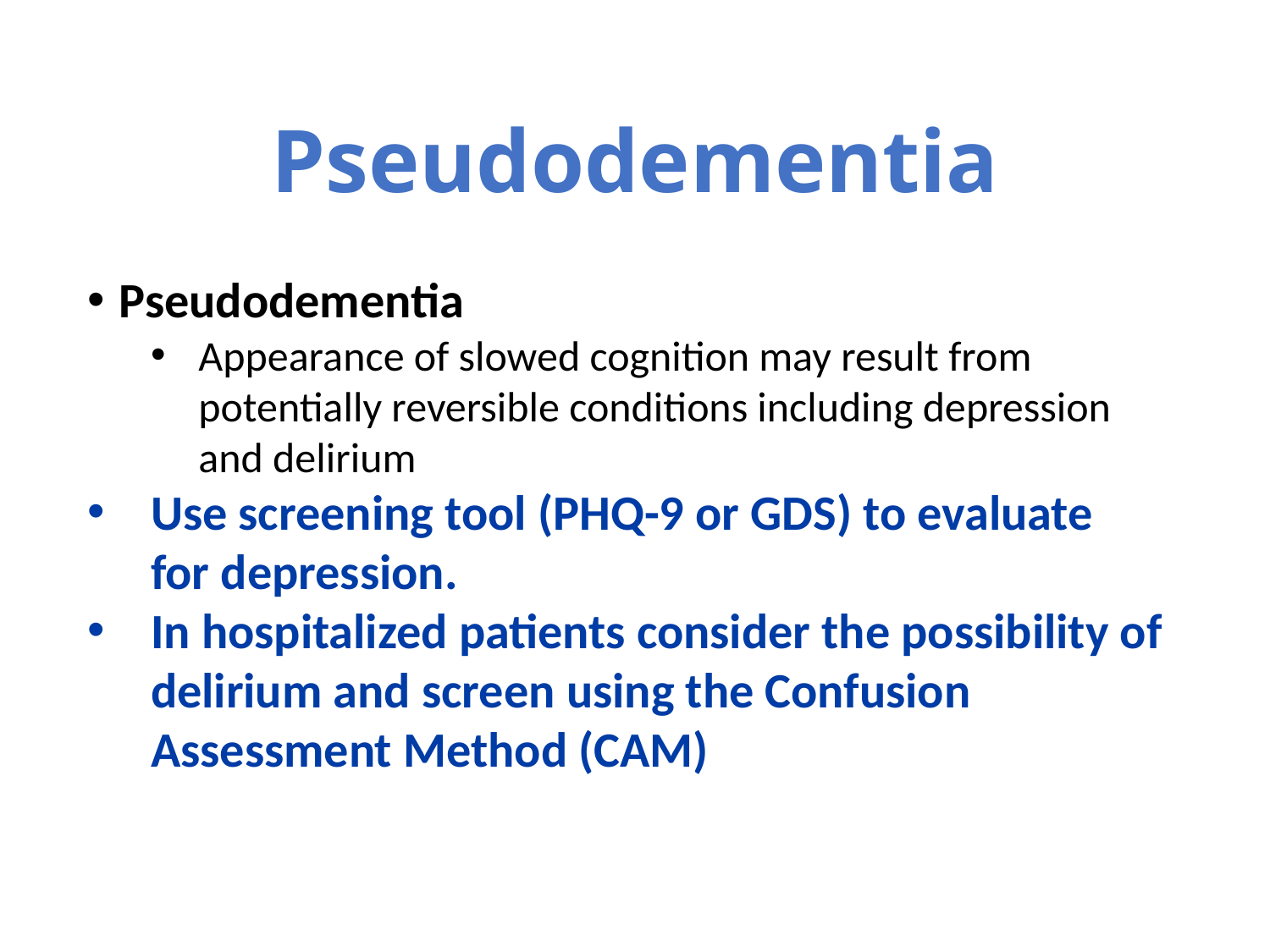

# Pseudodementia
Pseudodementia
Appearance of slowed cognition may result from potentially reversible conditions including depression and delirium
Use screening tool (PHQ-9 or GDS) to evaluate for depression.
In hospitalized patients consider the possibility of delirium and screen using the Confusion Assessment Method (CAM)

## Slide 12
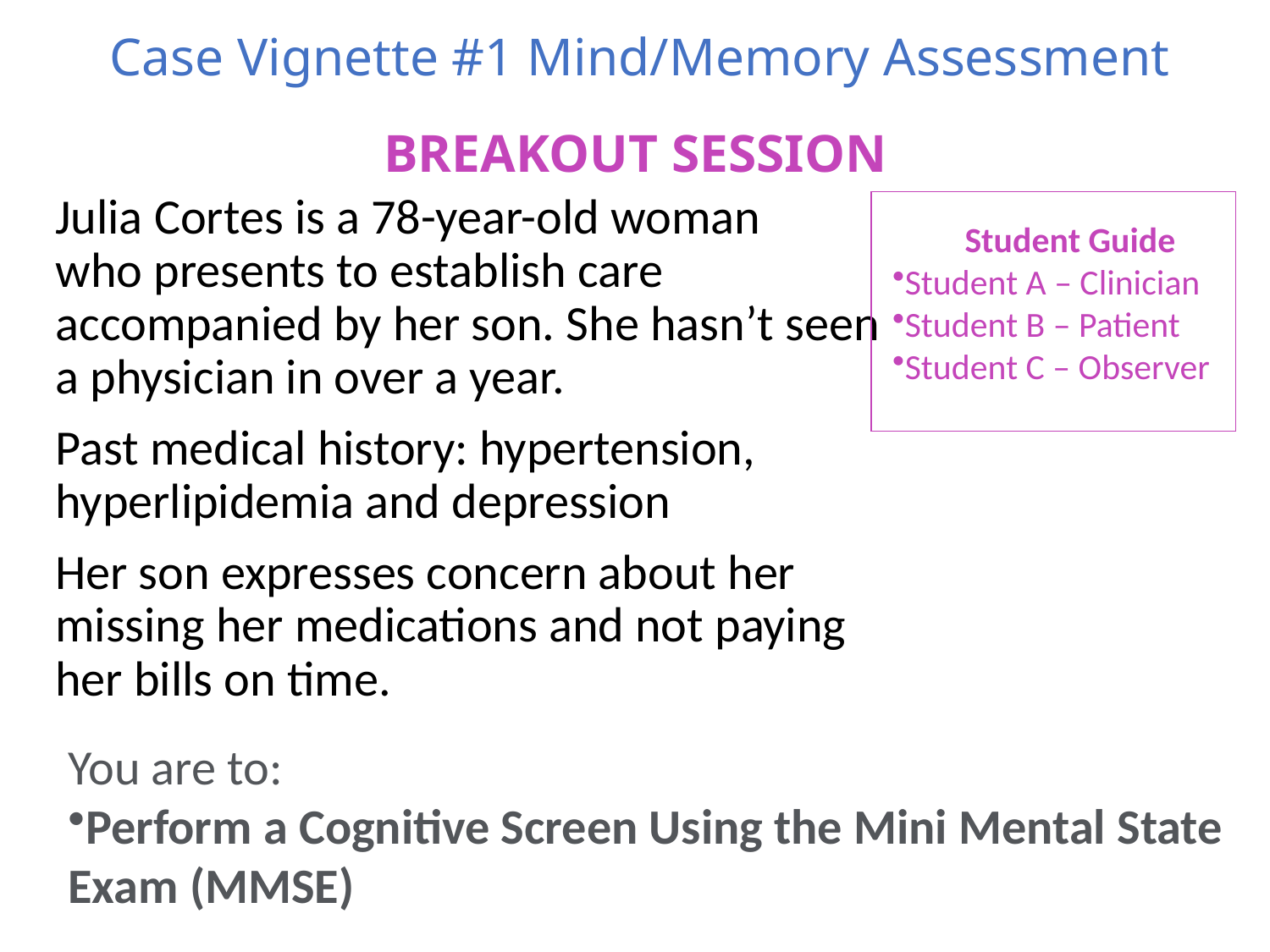

# Case Vignette #1 Mind/Memory Assessment  BREAKOUT SESSION
Julia Cortes is a 78-year-old woman who presents to establish care accompanied by her son. She hasn’t seen a physician in over a year.
Past medical history: hypertension, hyperlipidemia and depression
Her son expresses concern about her missing her medications and not paying her bills on time.
Student Guide​
Student A – Clinician​
Student B – Patient​
Student C – Observer​
​
You are to:​​
Perform a Cognitive Screen Using the Mini Mental State Exam (MMSE)

## Slide 13
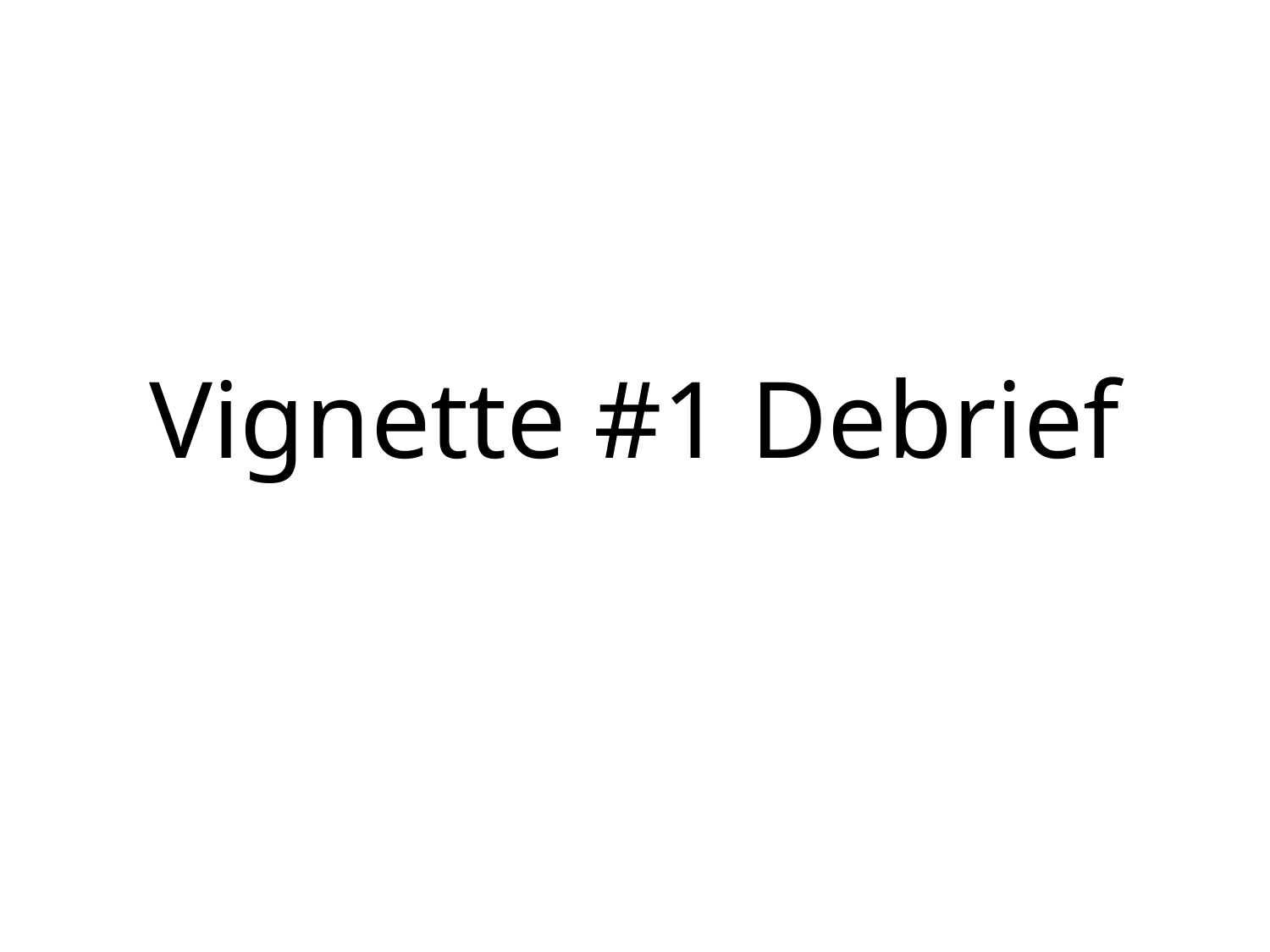

# Vignette #1 Debrief

## Slide 14
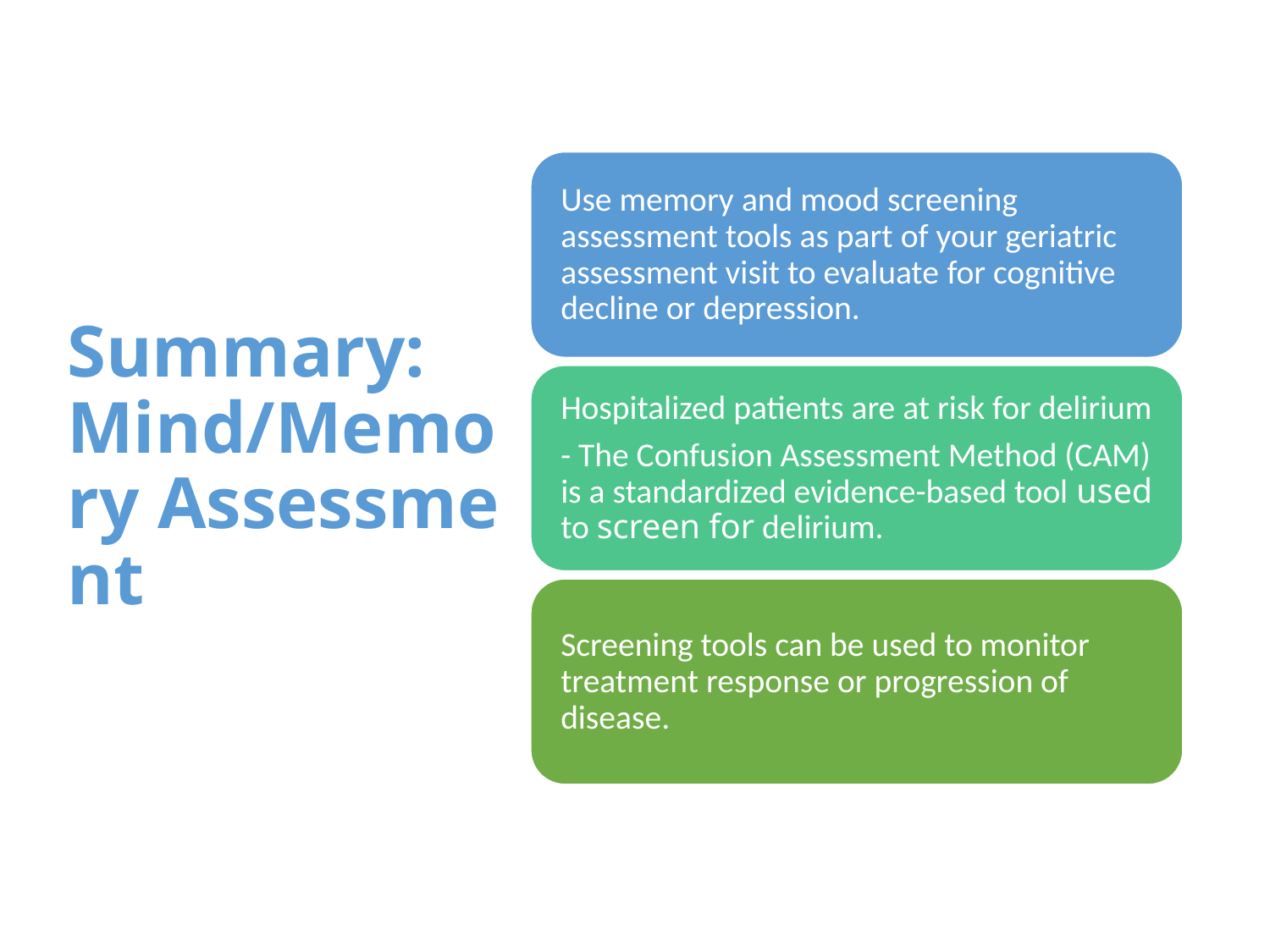

# Summary: Mind/Memory Assessment

## Slide 15
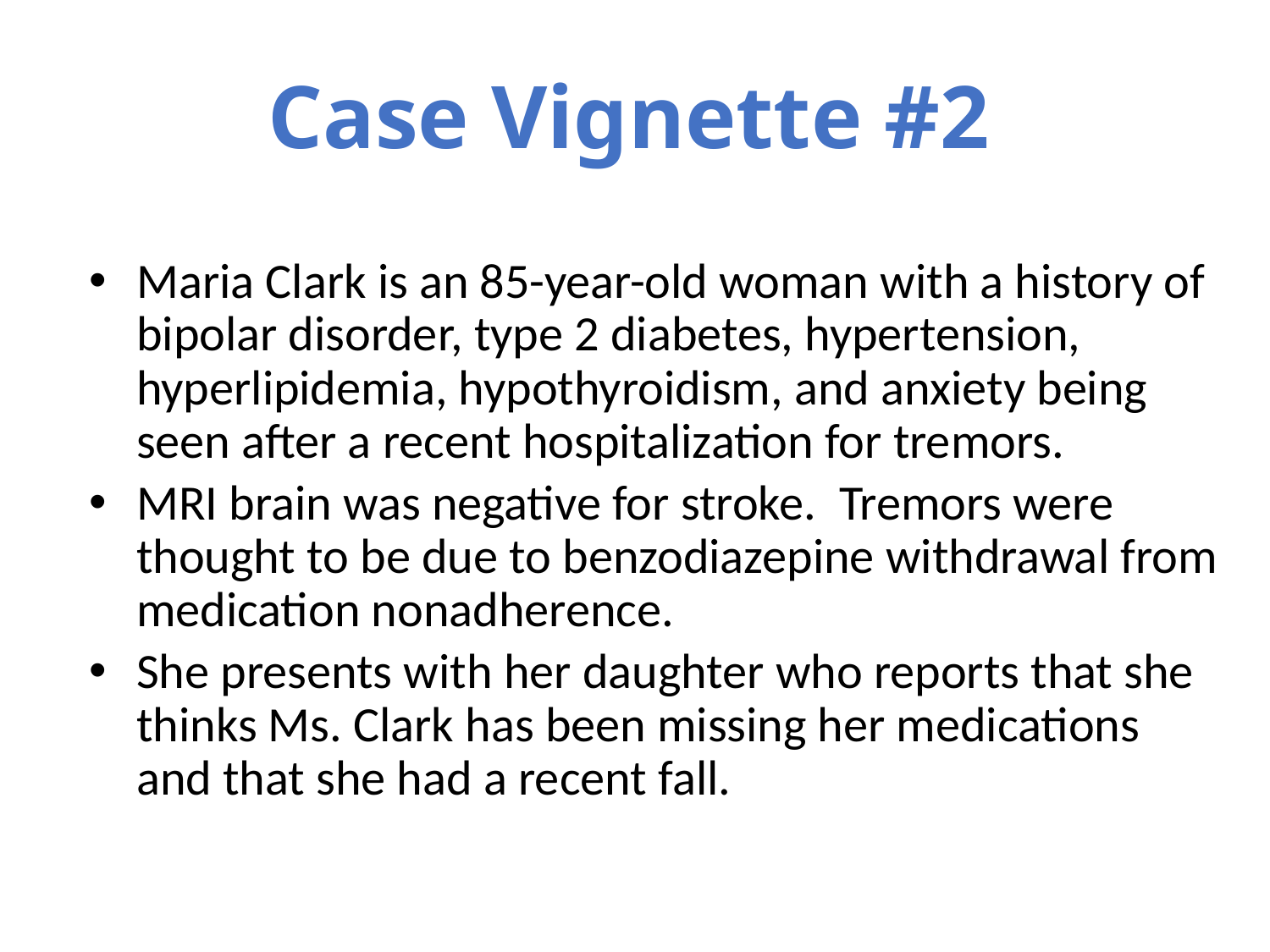

# Case Vignette #2
Maria Clark is an 85-year-old woman with a history of bipolar disorder, type 2 diabetes, hypertension, hyperlipidemia, hypothyroidism, and anxiety being seen after a recent hospitalization for tremors.
MRI brain was negative for stroke.  Tremors were thought to be due to benzodiazepine withdrawal from medication nonadherence.
She presents with her daughter who reports that she thinks Ms. Clark has been missing her medications and that she had a recent fall.

## Slide 16
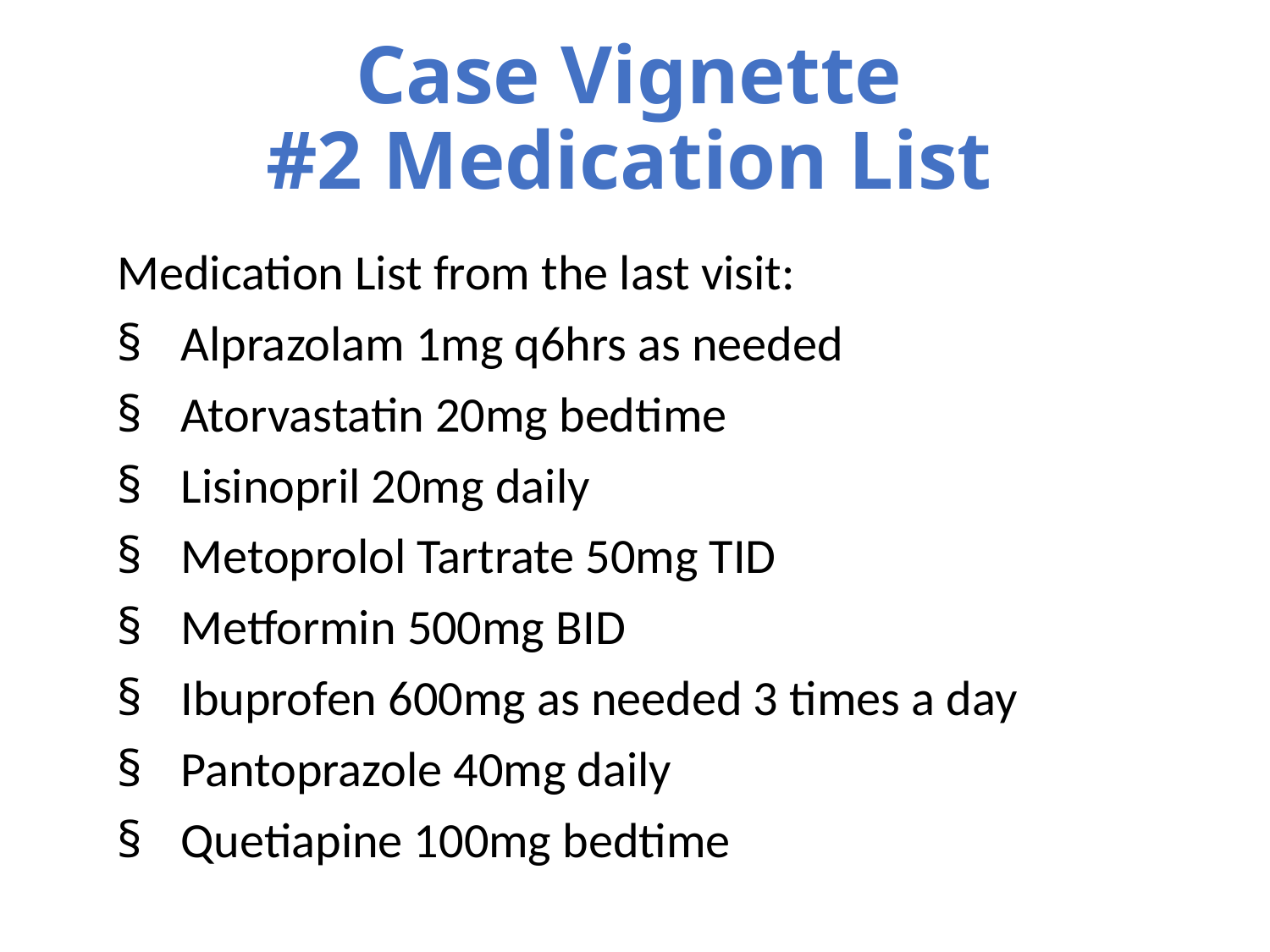

# Case Vignette #2 Medication List
Medication List from the last visit:
Alprazolam 1mg q6hrs as needed
Atorvastatin 20mg bedtime
Lisinopril 20mg daily
Metoprolol Tartrate 50mg TID
Metformin 500mg BID
Ibuprofen 600mg as needed 3 times a day
Pantoprazole 40mg daily
Quetiapine 100mg bedtime

## Slide 17
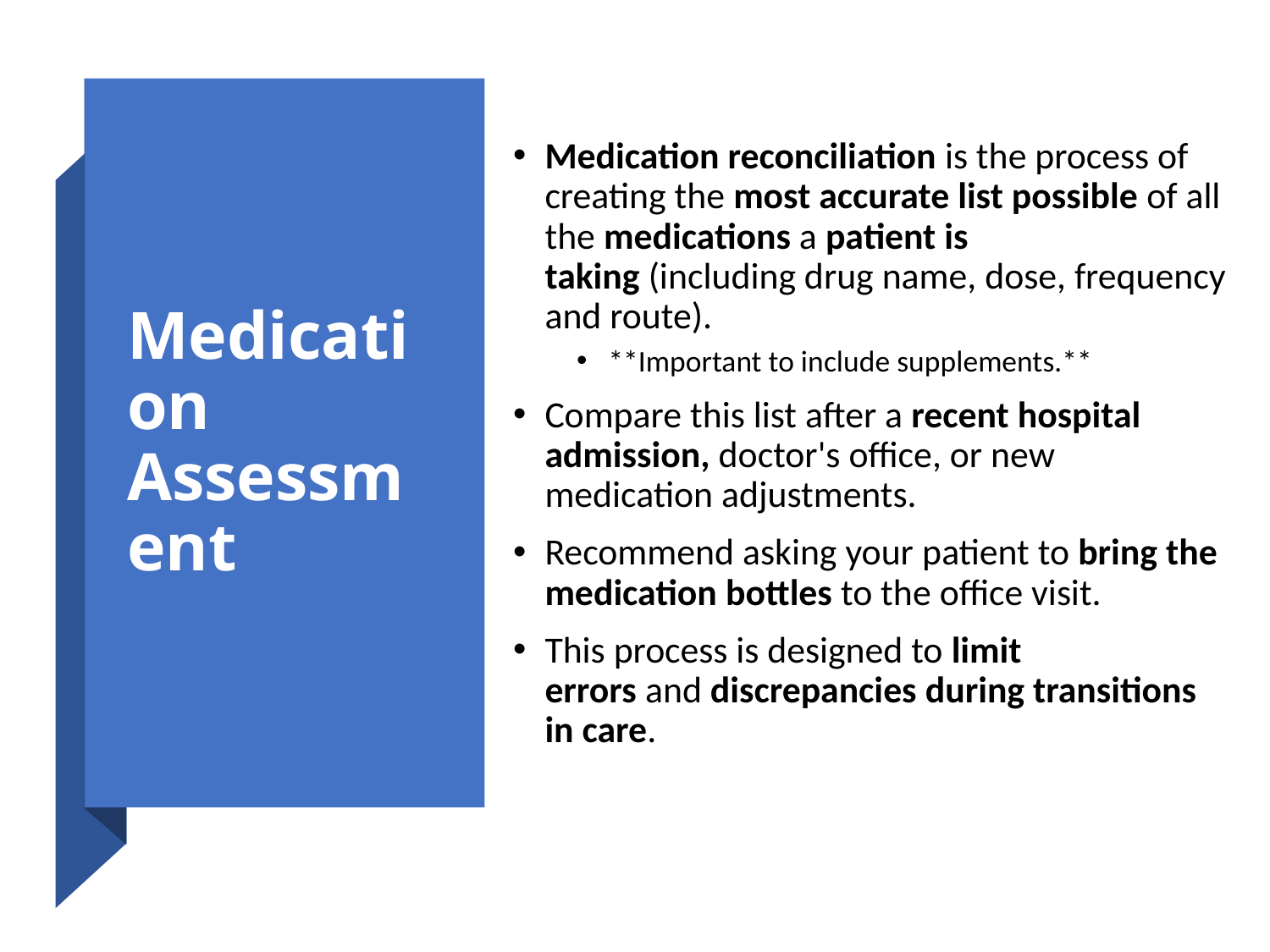

Medication reconciliation is the process of creating the most accurate list possible of all the medications a patient is taking (including drug name, dose, frequency and route).
**Important to include supplements.**
Compare this list after a recent hospital admission, doctor's office, or new medication adjustments.
Recommend asking your patient to bring the medication bottles to the office visit.
This process is designed to limit errors and discrepancies during transitions in care.
# Medication Assessment

## Slide 18
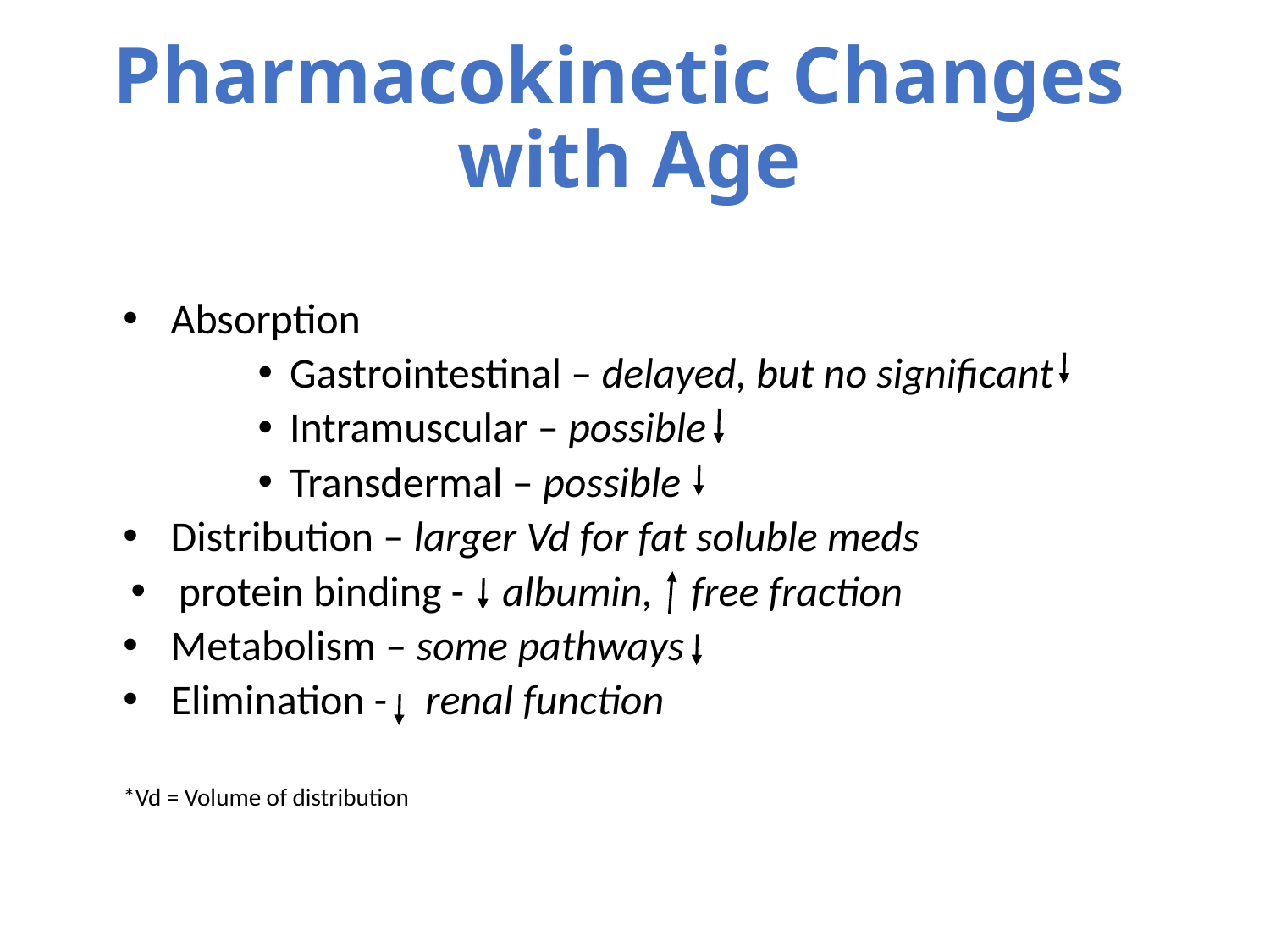

# Pharmacokinetic Changes  with Age
Absorption
Gastrointestinal – delayed, but no significant
Intramuscular – possible
Transdermal – possible
Distribution – larger Vd for fat soluble meds
protein binding -    albumin,    free fraction
Metabolism – some pathways
Elimination -    renal function
*Vd = Volume of distribution

## Slide 19
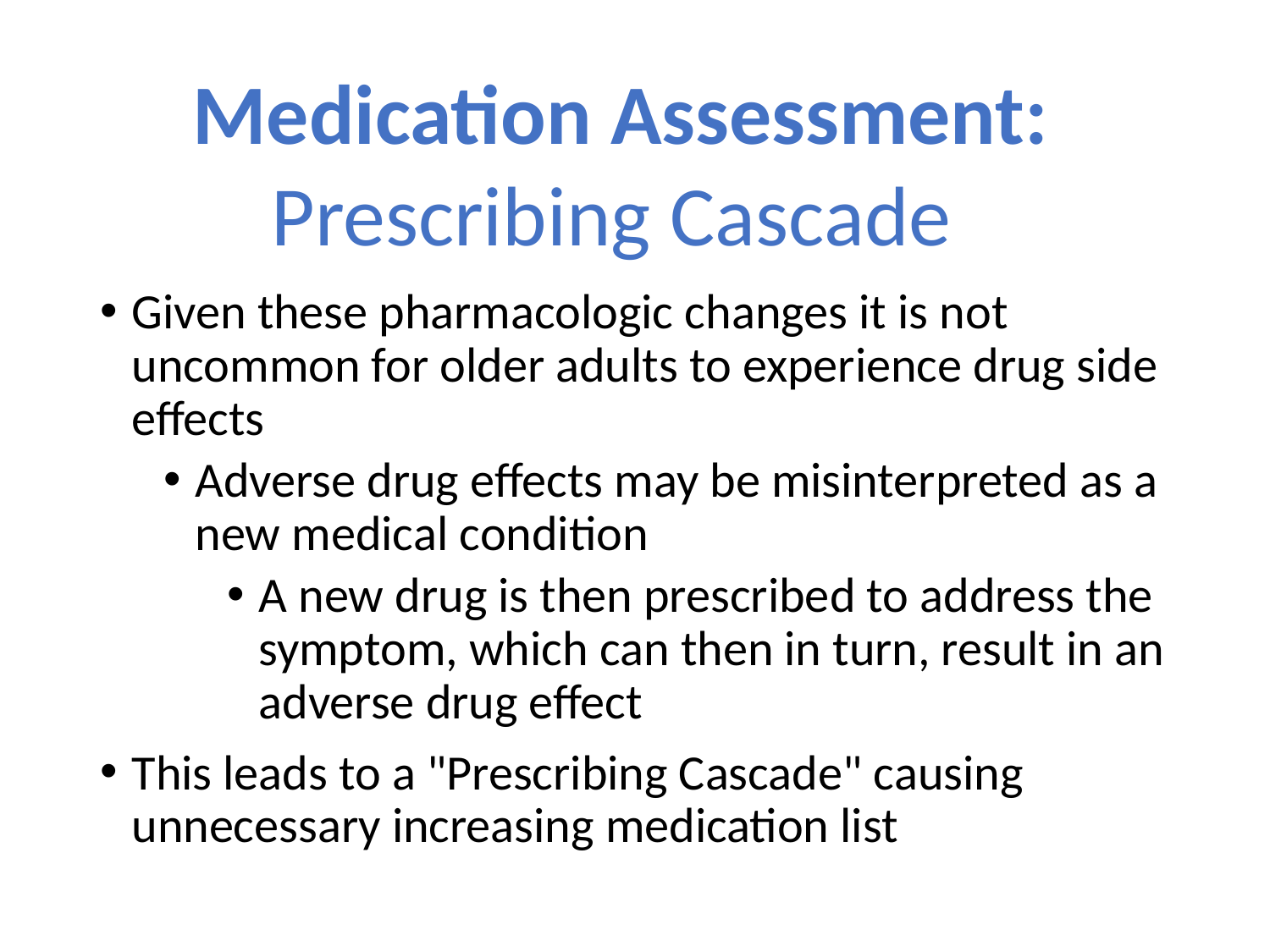

Medication Assessment:
Prescribing Cascade
Given these pharmacologic changes it is not uncommon for older adults to experience drug side effects
Adverse drug effects may be misinterpreted as a new medical condition
A new drug is then prescribed to address the symptom, which can then in turn, result in an adverse drug effect
This leads to a "Prescribing Cascade" causing unnecessary increasing medication list

## Slide 20
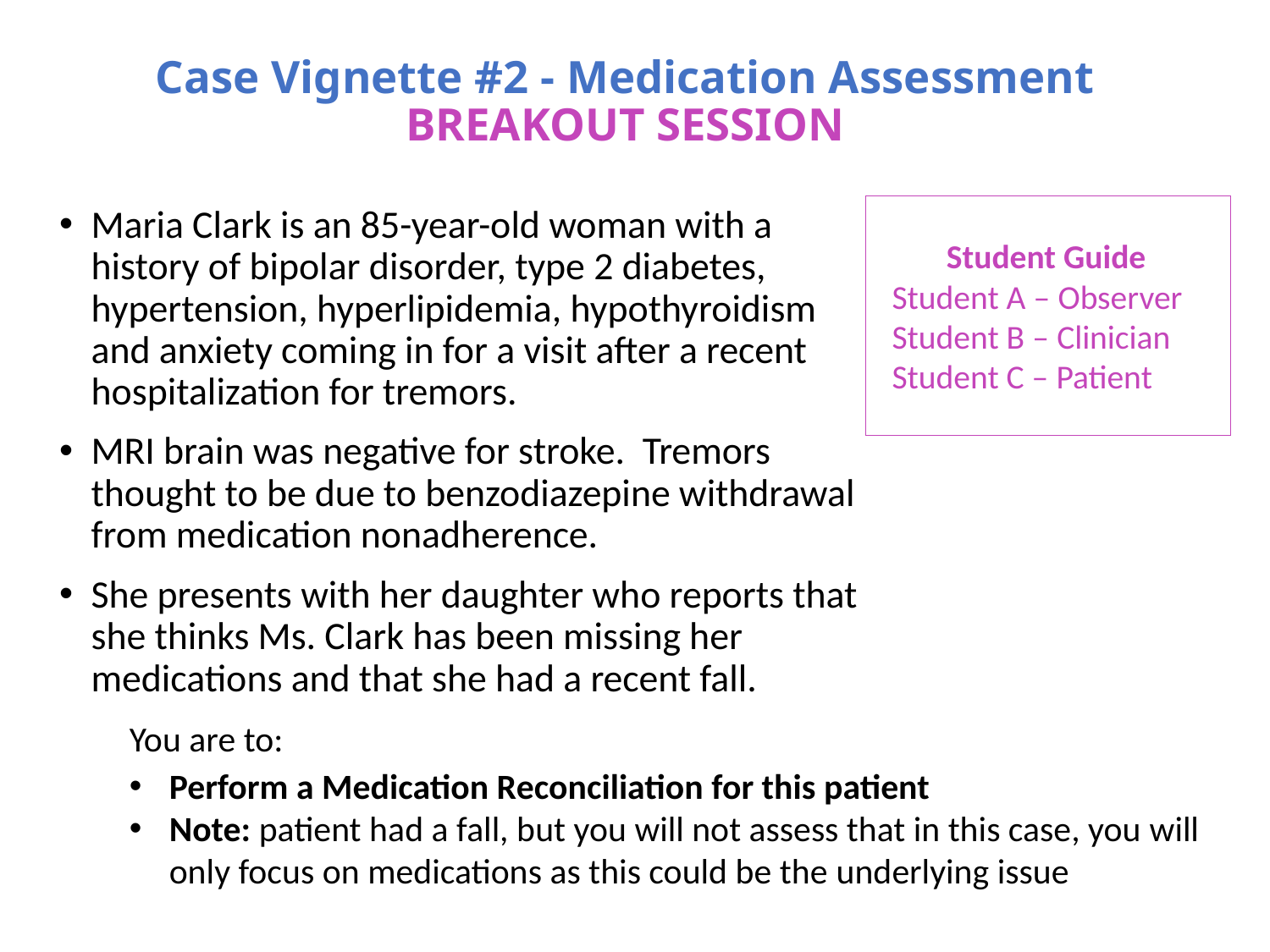

# Case Vignette #2 - Medication Assessment  BREAKOUT SESSION
Maria Clark is an 85-year-old woman with a history of bipolar disorder, type 2 diabetes, hypertension, hyperlipidemia, hypothyroidism and anxiety coming in for a visit after a recent hospitalization for tremors.
MRI brain was negative for stroke.  Tremors thought to be due to benzodiazepine withdrawal from medication nonadherence.
She presents with her daughter who reports that she thinks Ms. Clark has been missing her medications and that she had a recent fall.
Student Guide
Student A – Observer
Student B – Clinician
Student C – Patient
You are to:
Perform a Medication Reconciliation for this patient
Note: patient had a fall, but you will not assess that in this case, you will only focus on medications as this could be the underlying issue

## Slide 21
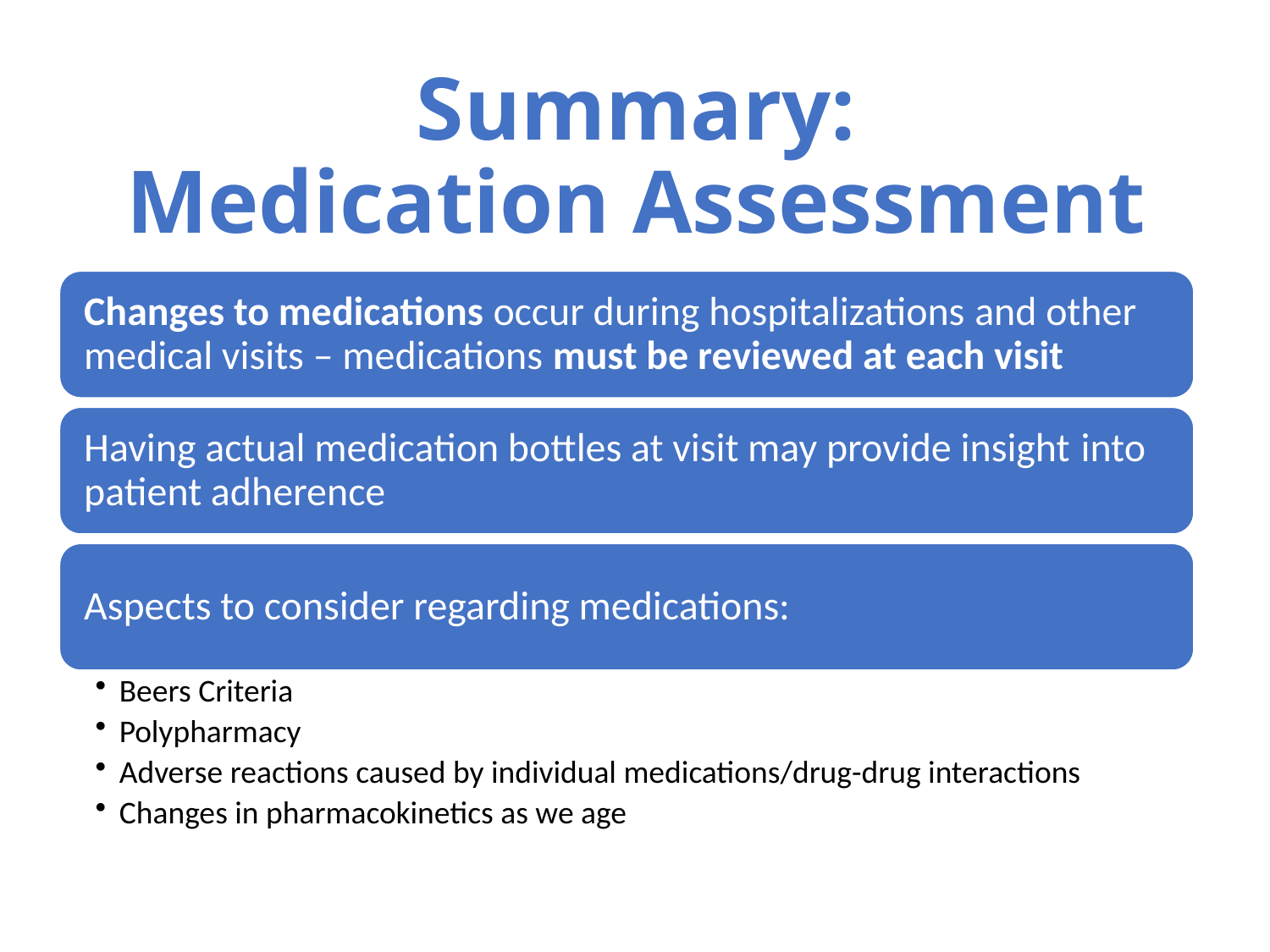

# Summary: Medication Assessment

## Slide 22
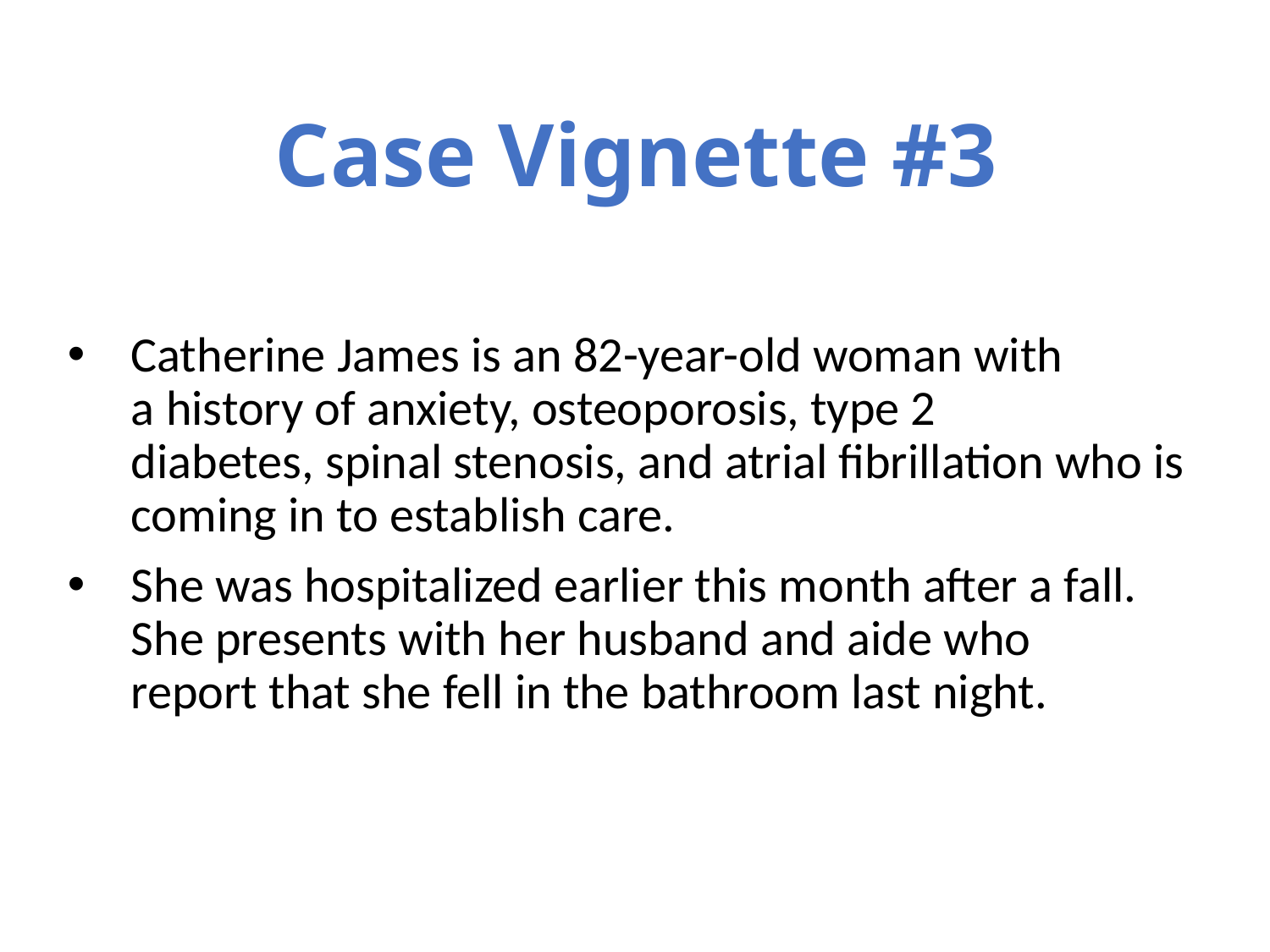

# Case Vignette #3
Catherine James is an 82-year-old woman with a history of anxiety, osteoporosis, type 2 diabetes, spinal stenosis, and atrial fibrillation who is coming in to establish care.
She was hospitalized earlier this month after a fall. She presents with her husband and aide who report that she fell in the bathroom last night.

## Slide 23
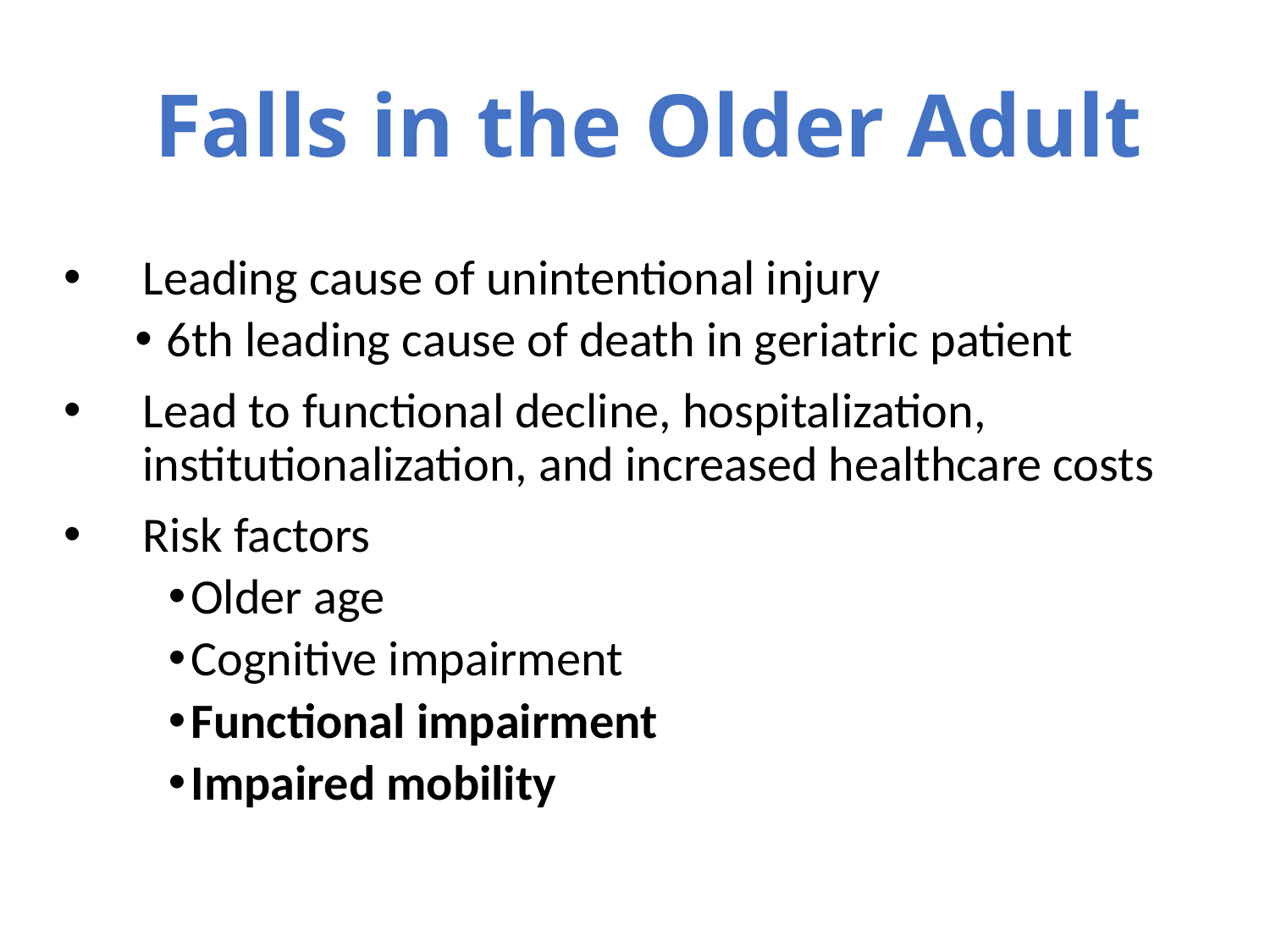

# Falls in the Older Adult
Leading cause of unintentional injury
6th leading cause of death in geriatric patient
Lead to functional decline, hospitalization, institutionalization, and increased healthcare costs
Risk factors
Older age
Cognitive impairment
Functional impairment
Impaired mobility

## Slide 24
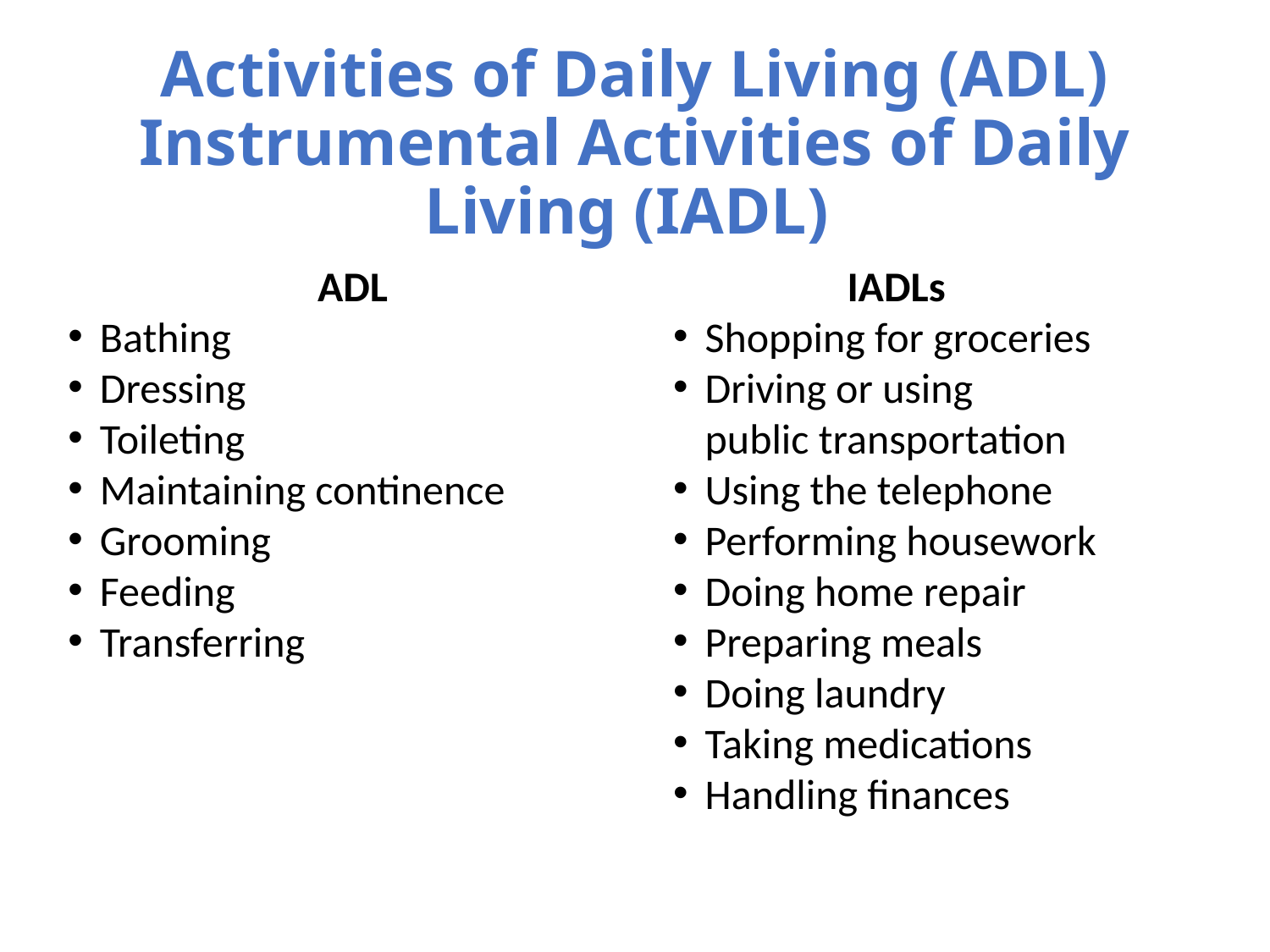

# Activities of Daily Living (ADL) Instrumental Activities of Daily Living (IADL)
ADL
Bathing
Dressing
Toileting
Maintaining continence
Grooming
Feeding
Transferring
IADLs
Shopping for groceries
Driving or using public transportation
Using the telephone
Performing housework
Doing home repair
Preparing meals
Doing laundry
Taking medications
Handling finances

## Slide 25
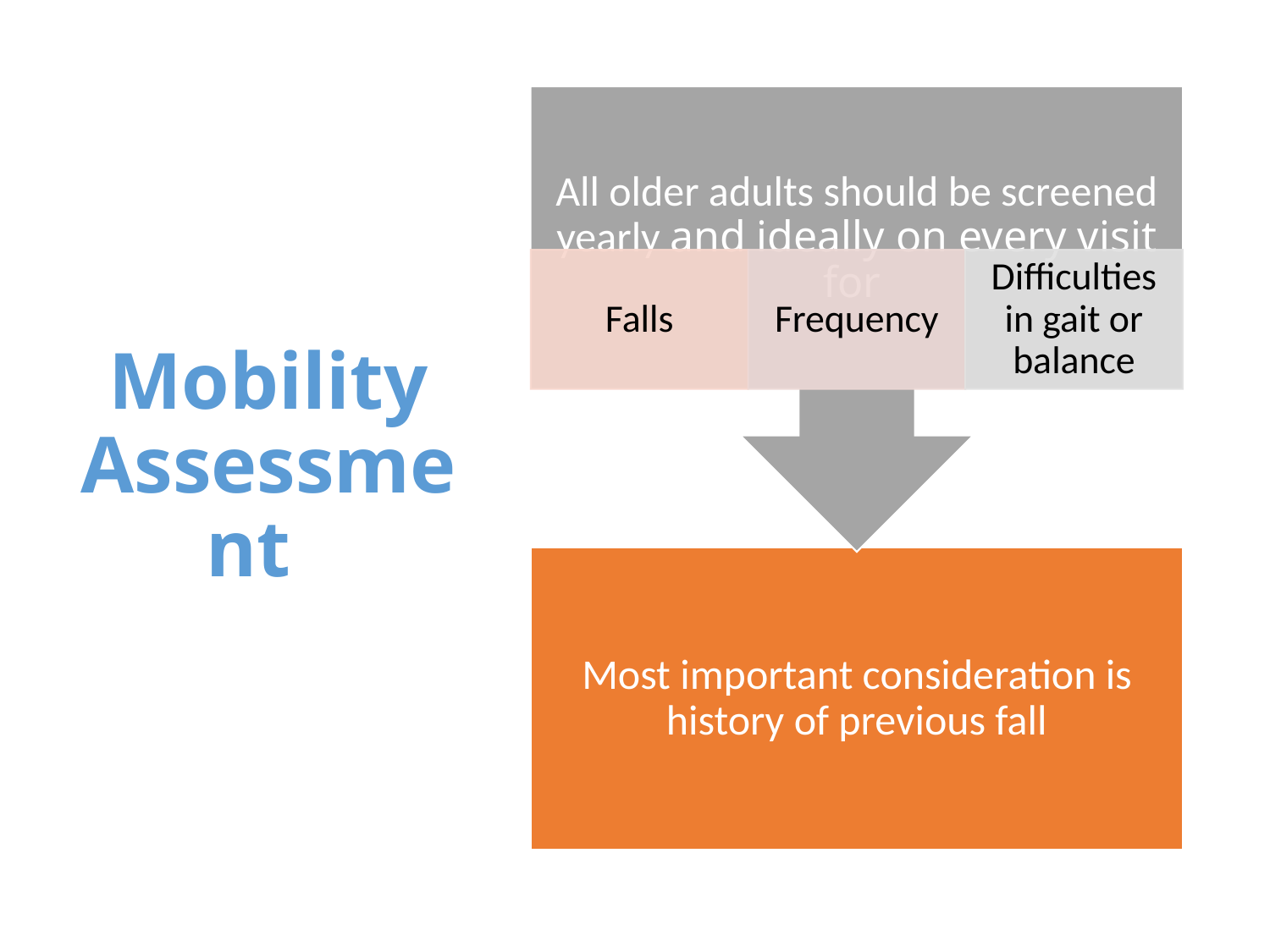

# Mobility Assessment

## Slide 26
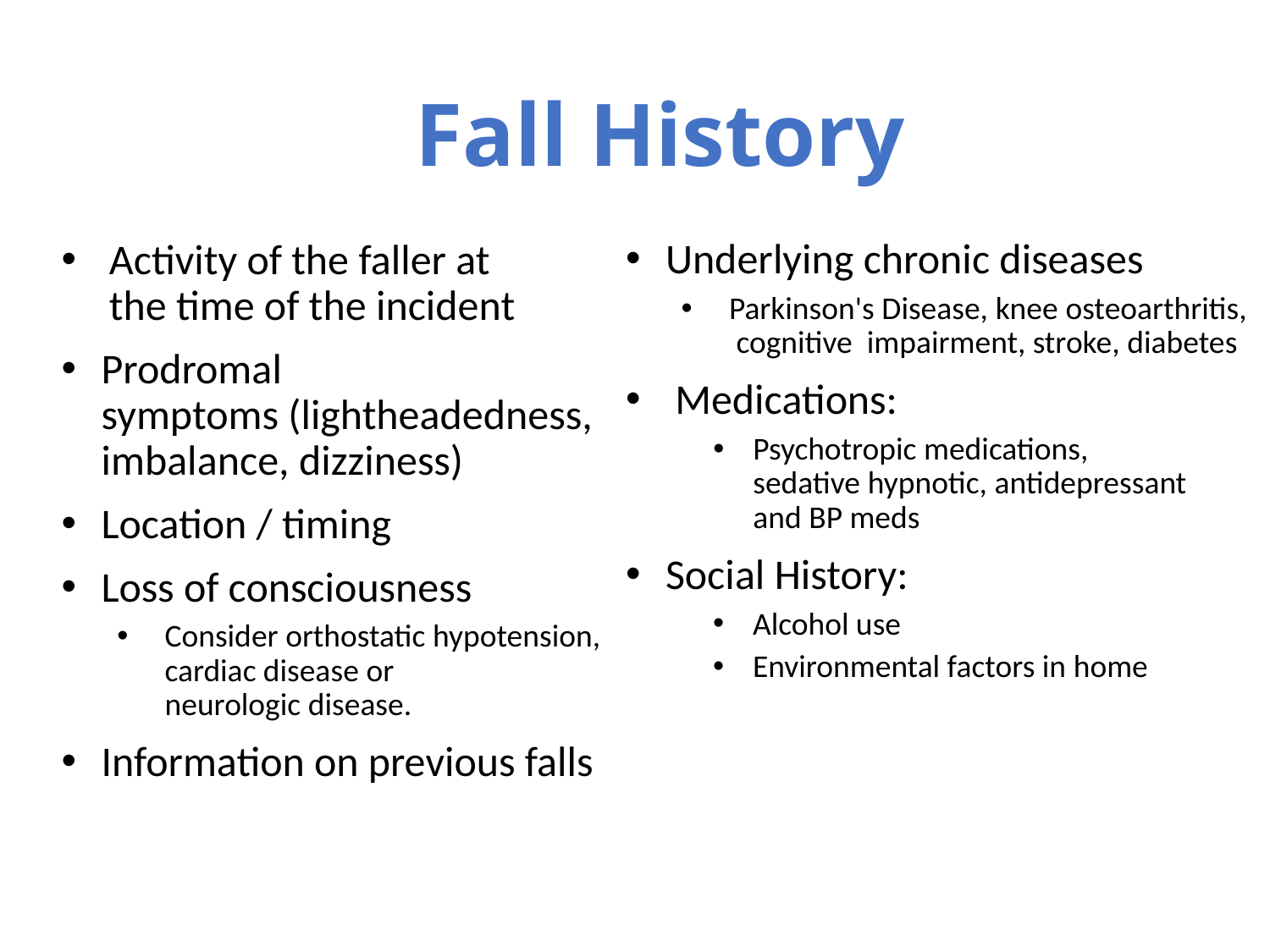

# Fall History
Underlying chronic diseases
Parkinson's Disease, knee osteoarthritis, cognitive  impairment, stroke, diabetes
 Medications:
Psychotropic medications, sedative hypnotic, antidepressant and BP meds
Social History:
Alcohol use
Environmental factors in home
Activity of the faller at the time of the incident
Prodromal symptoms (lightheadedness, imbalance, dizziness)
Location / timing
Loss of consciousness
Consider orthostatic hypotension, cardiac disease or neurologic disease.
Information on previous falls

## Slide 27
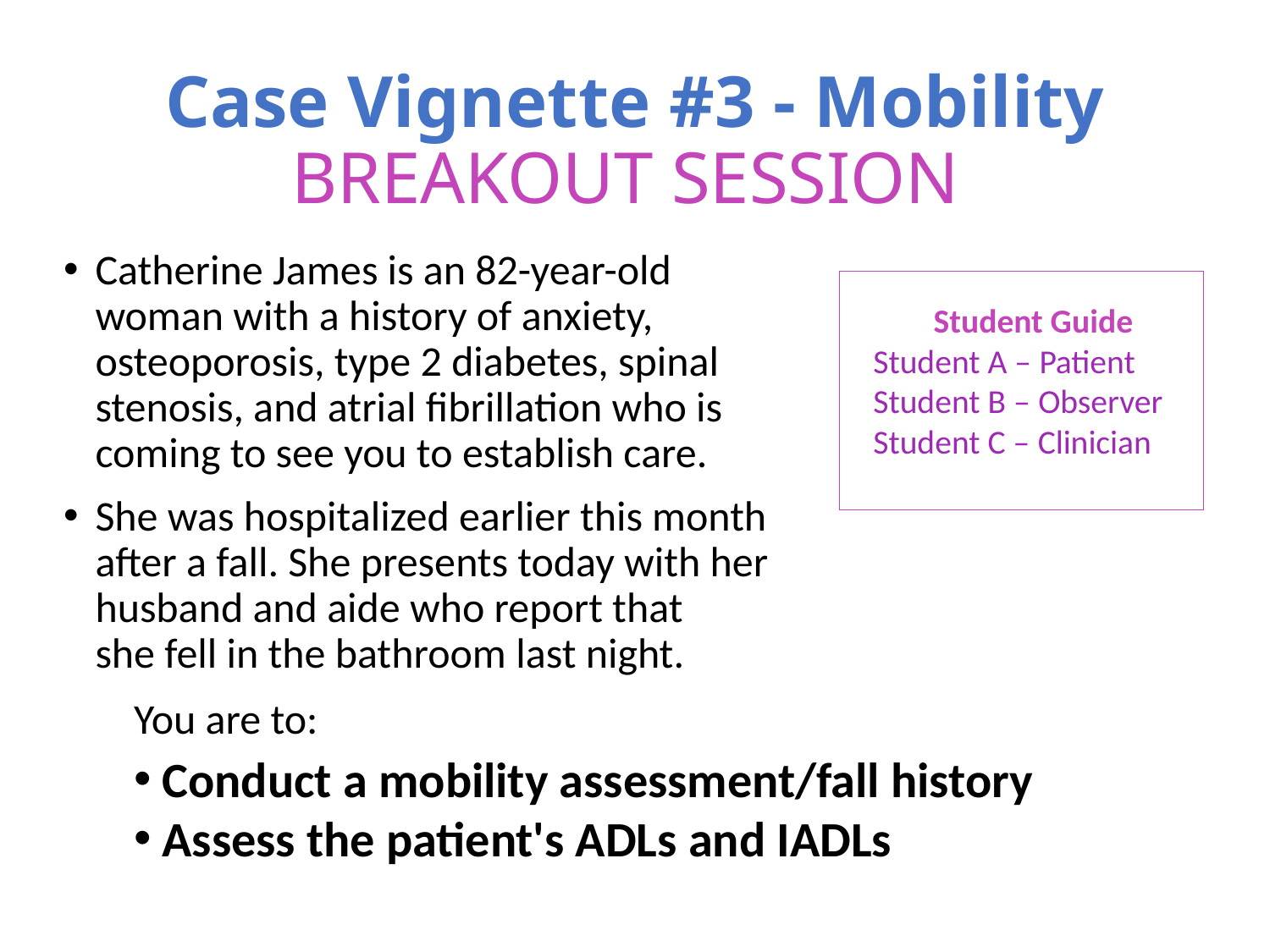

# Case Vignette #3 - Mobility BREAKOUT SESSION
Catherine James is an 82-year-old woman with a history of anxiety, osteoporosis, type 2 diabetes, spinal stenosis, and atrial fibrillation who is coming to see you to establish care.
She was hospitalized earlier this month after a fall. She presents today with her husband and aide who report that she fell in the bathroom last night.
Student Guide
Student A – Patient
Student B – Observer
Student C – Clinician
You are to:
 Conduct a mobility assessment/fall history
 Assess the patient's ADLs and IADLs

## Slide 28
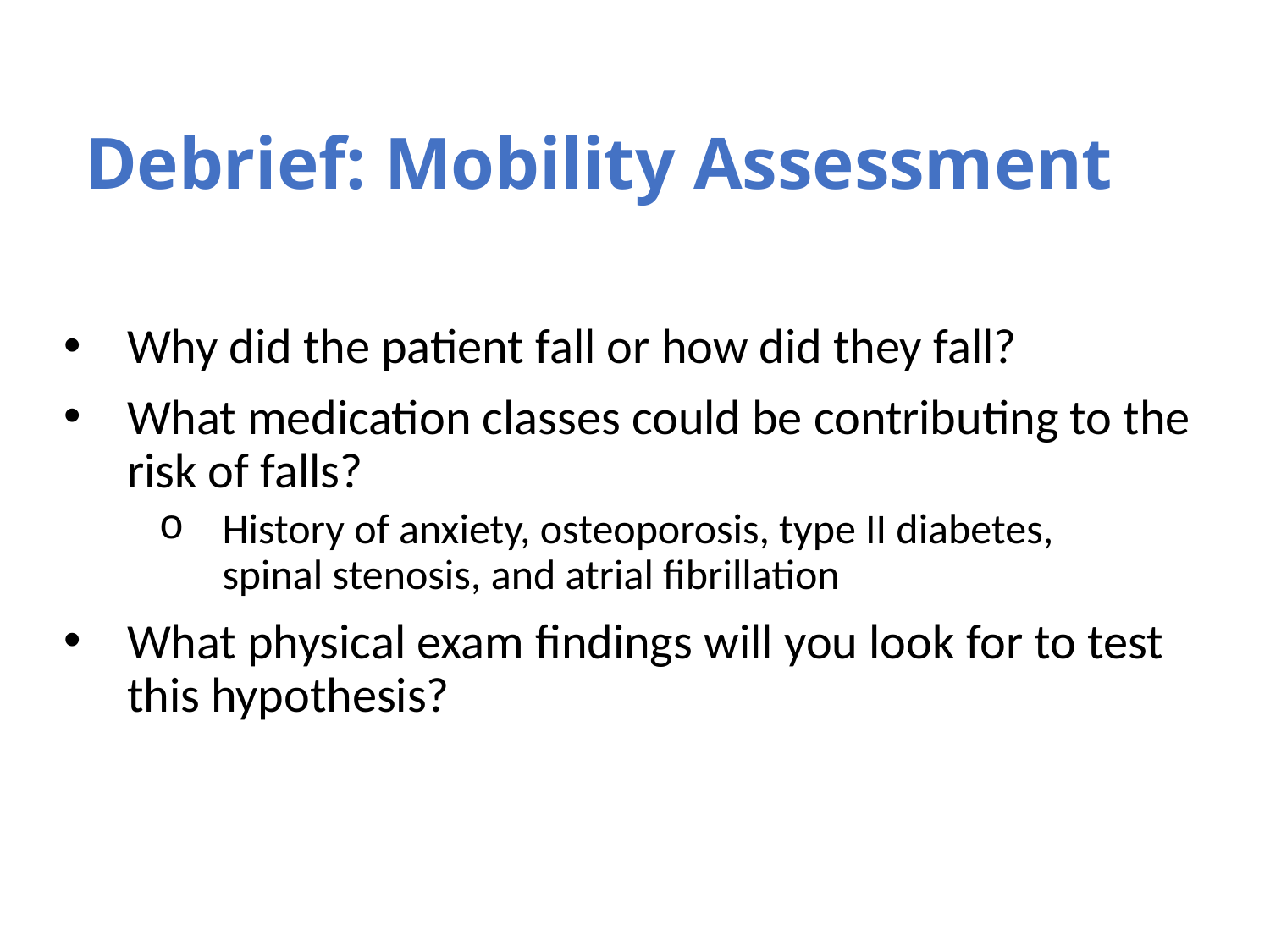

# Debrief: Mobility Assessment
Why did the patient fall or how did they fall?
What medication classes could be contributing to the risk of falls?
History of anxiety, osteoporosis, type II diabetes, spinal stenosis, and atrial fibrillation
What physical exam findings will you look for to test this hypothesis?

## Slide 29
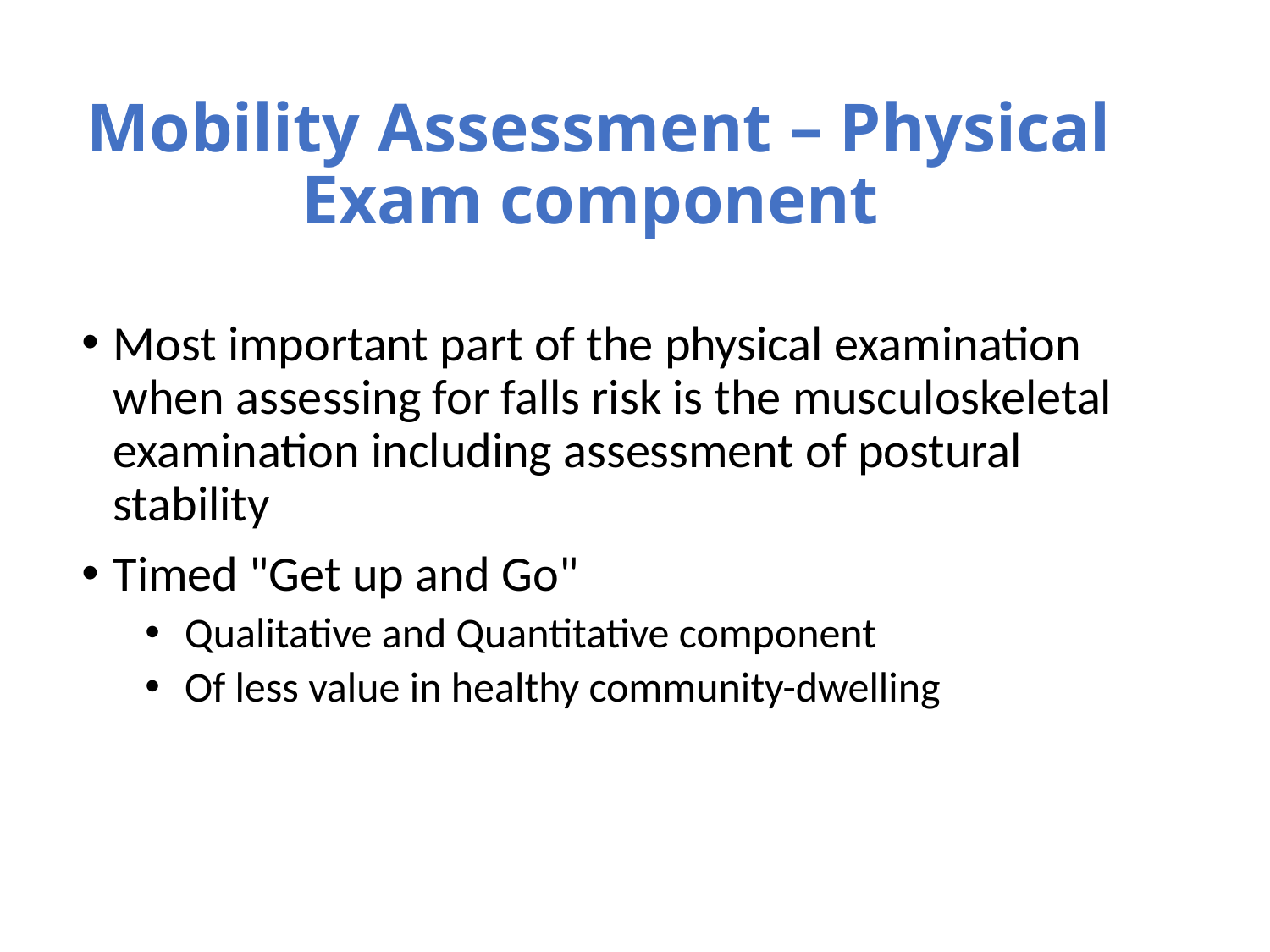

# Mobility Assessment – Physical Exam component
Most important part of the physical examination when assessing for falls risk is the musculoskeletal examination including assessment of postural stability
Timed "Get up and Go"
Qualitative and Quantitative component
Of less value in healthy community-dwelling

## Slide 30
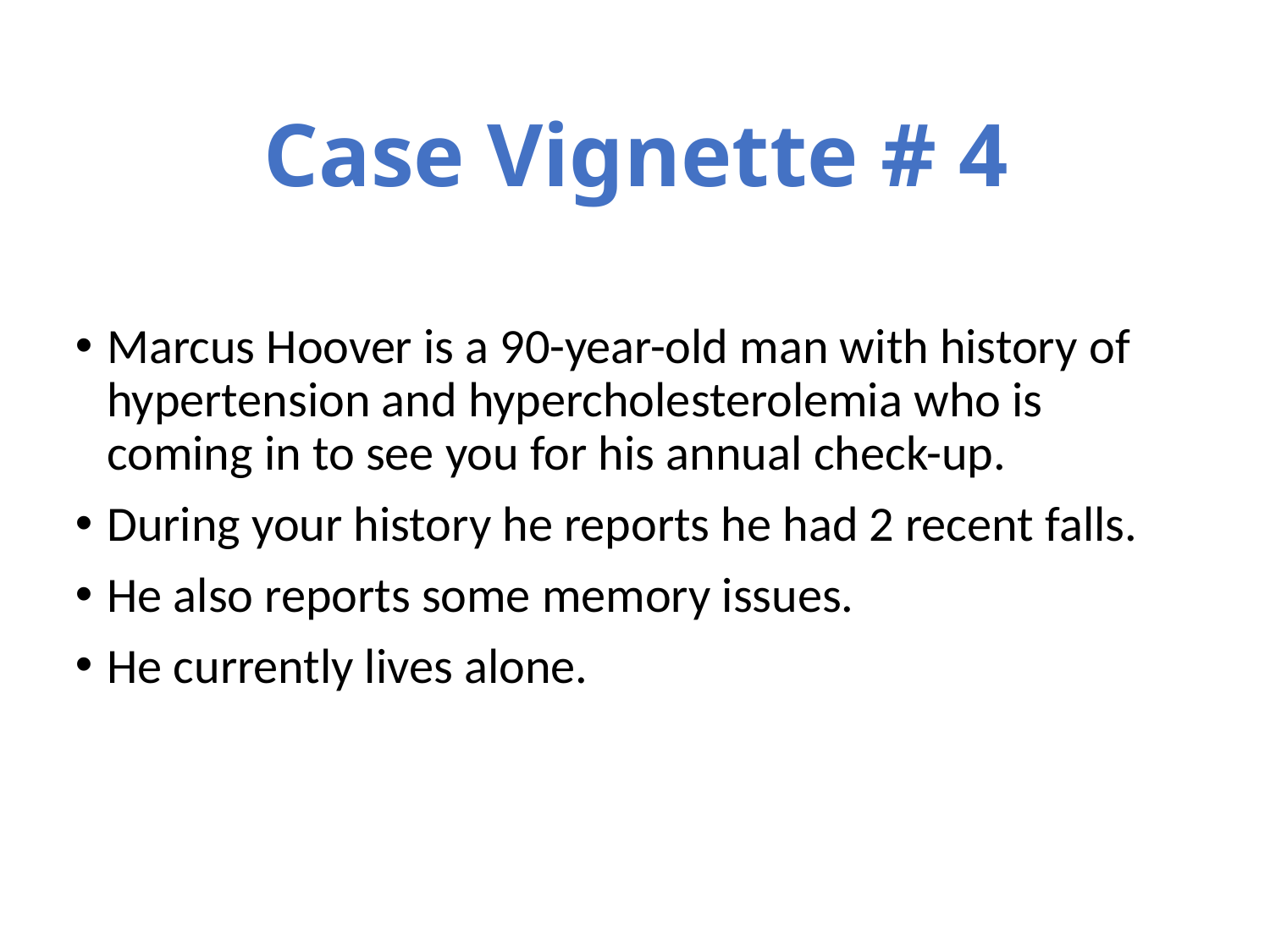

# Case Vignette # 4
Marcus Hoover is a 90-year-old man with history of hypertension and hypercholesterolemia who is coming in to see you for his annual check-up.
During your history he reports he had 2 recent falls.
He also reports some memory issues.
He currently lives alone.

## Slide 31
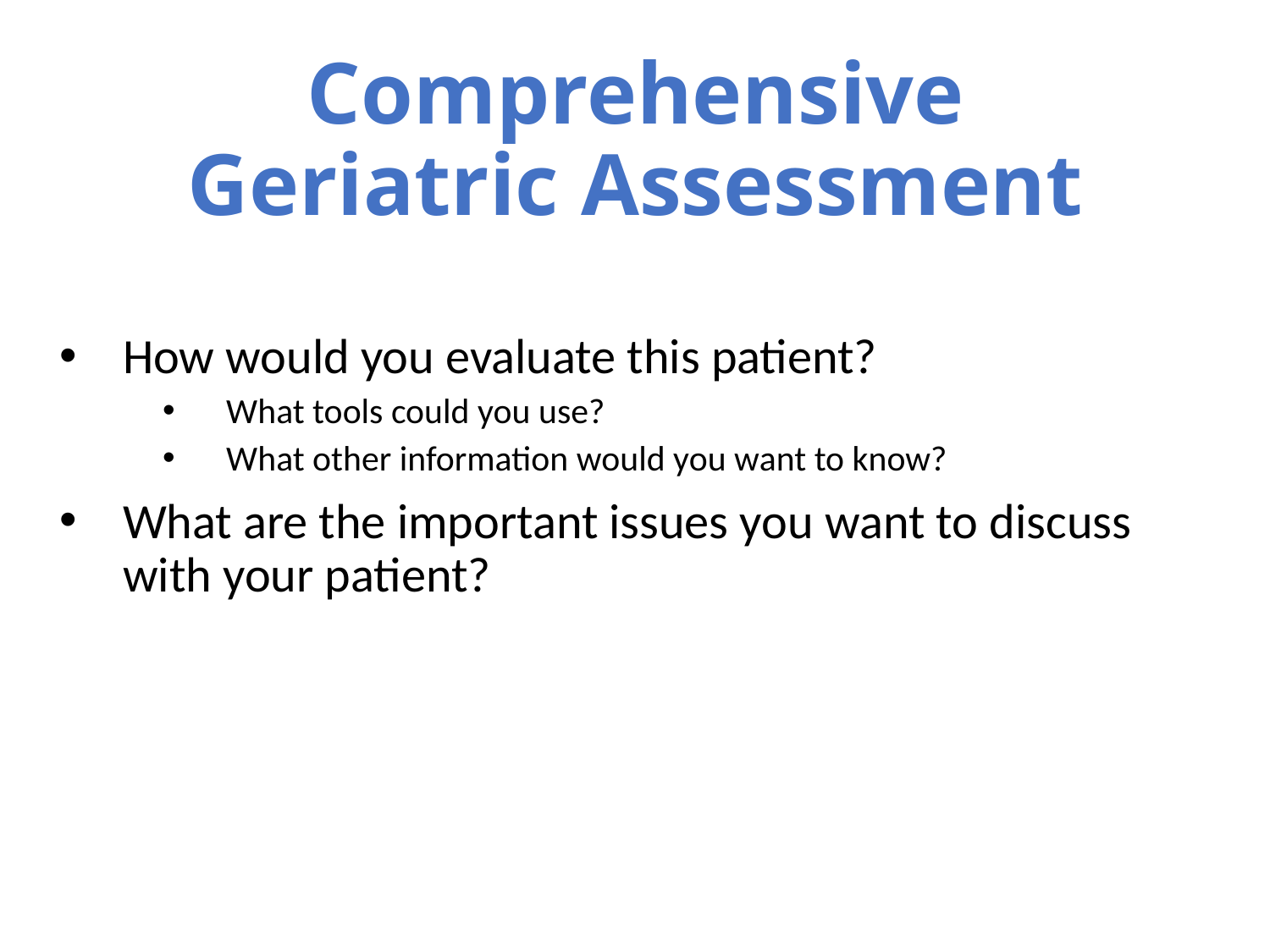

# Comprehensive Geriatric Assessment
How would you evaluate this patient?
What tools could you use?
What other information would you want to know?
What are the important issues you want to discuss with your patient?

## Slide 32
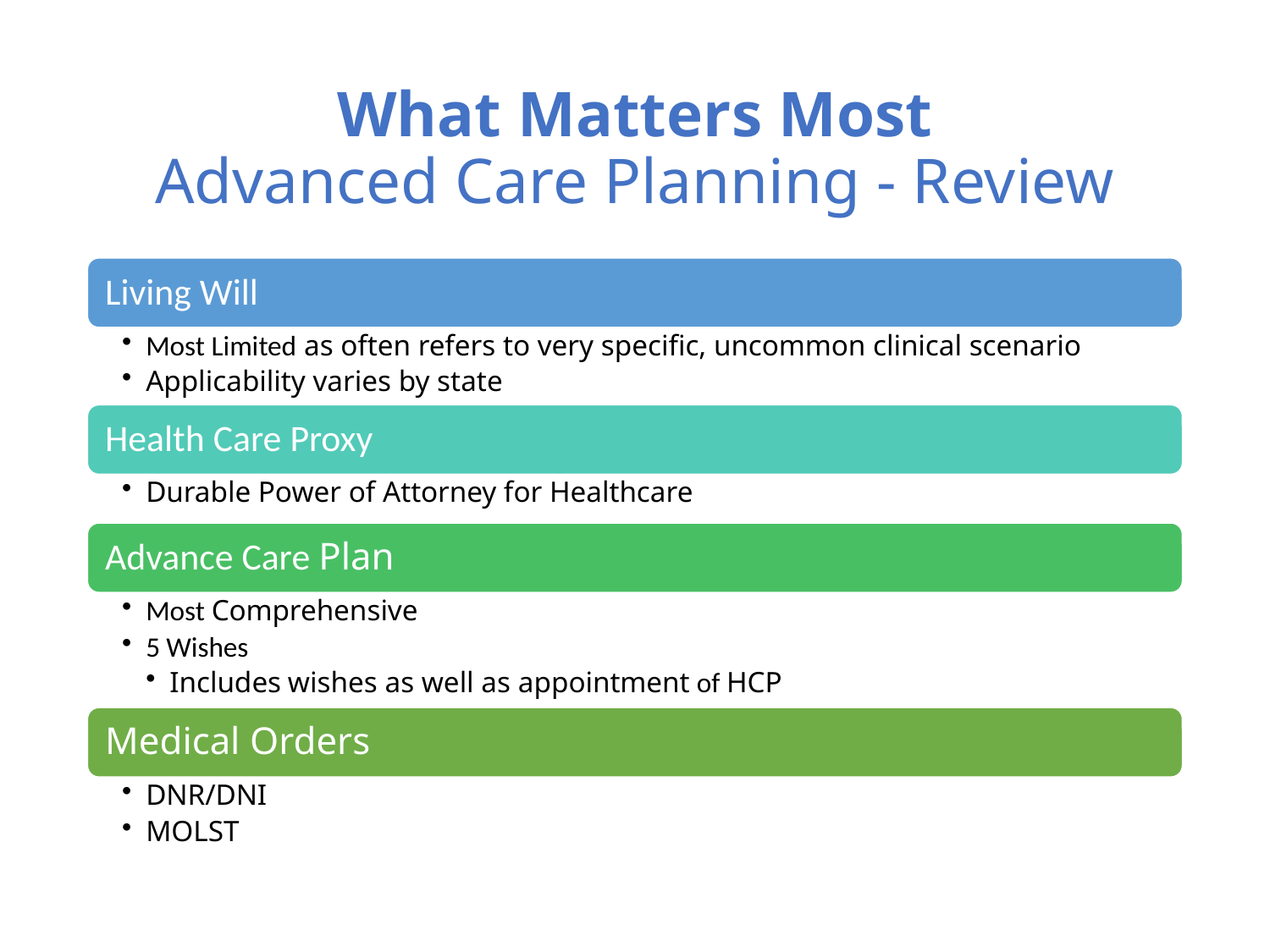

# What Matters Most
Advanced Care Planning - Review

## Slide 33
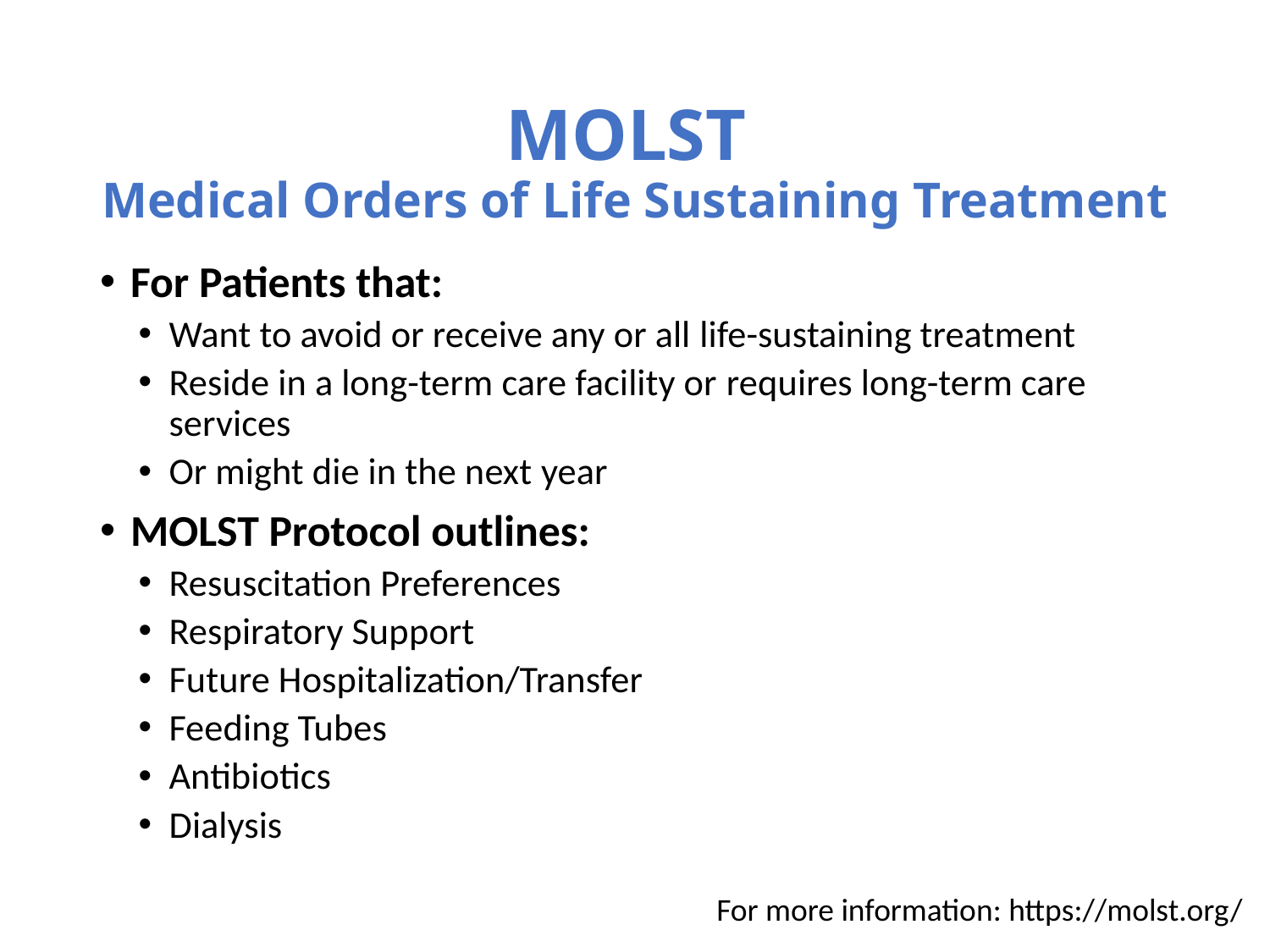

# MOLST Medical Orders of Life Sustaining Treatment
For Patients that:
Want to avoid or receive any or all life-sustaining treatment
Reside in a long-term care facility or requires long-term care services
Or might die in the next year
MOLST Protocol outlines:
Resuscitation Preferences
Respiratory Support
Future Hospitalization/Transfer
Feeding Tubes
Antibiotics
Dialysis
For more information: https://molst.org/

## Slide 34
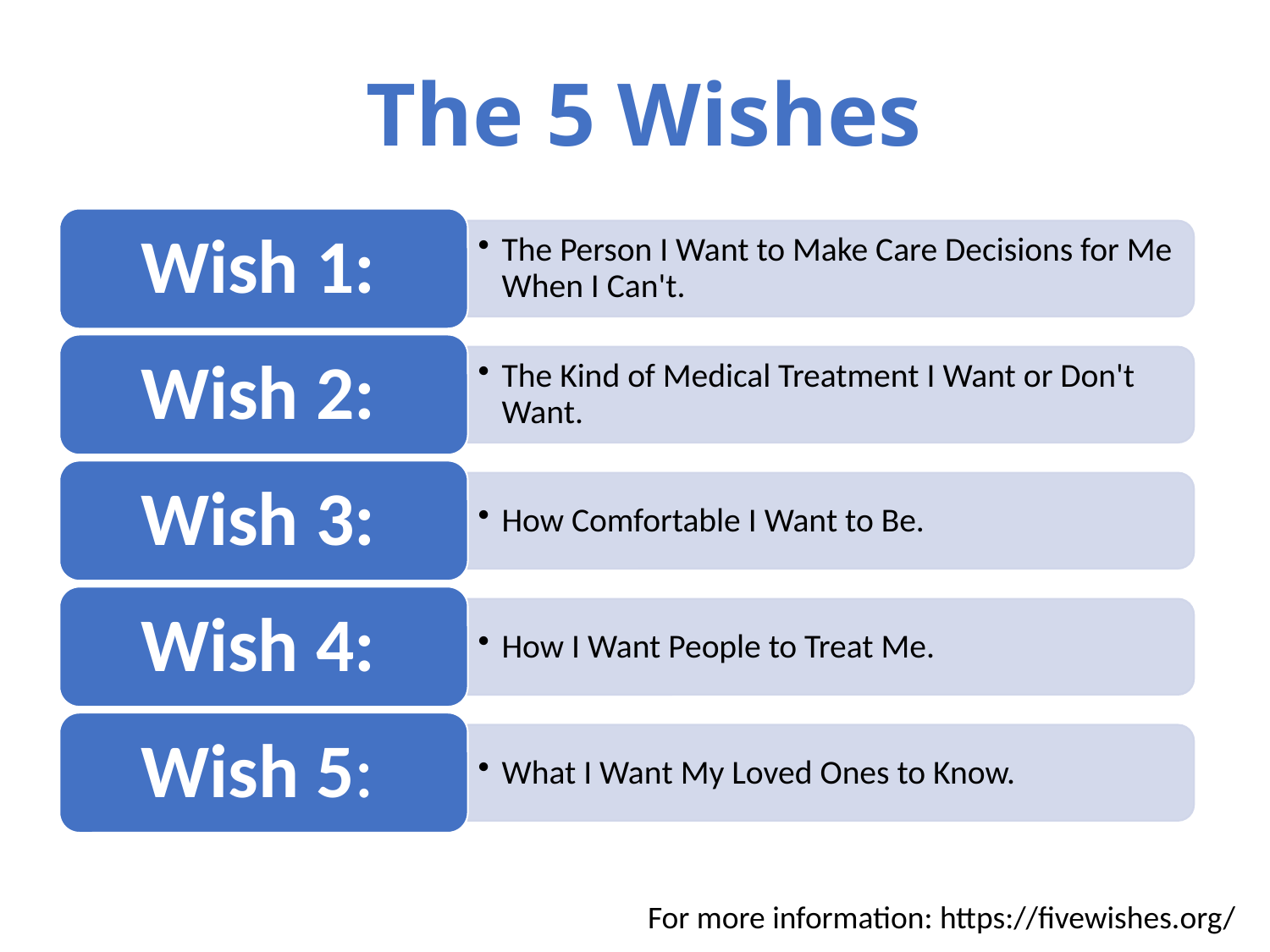

# The 5 Wishes
For more information: https://fivewishes.org/

## Slide 35
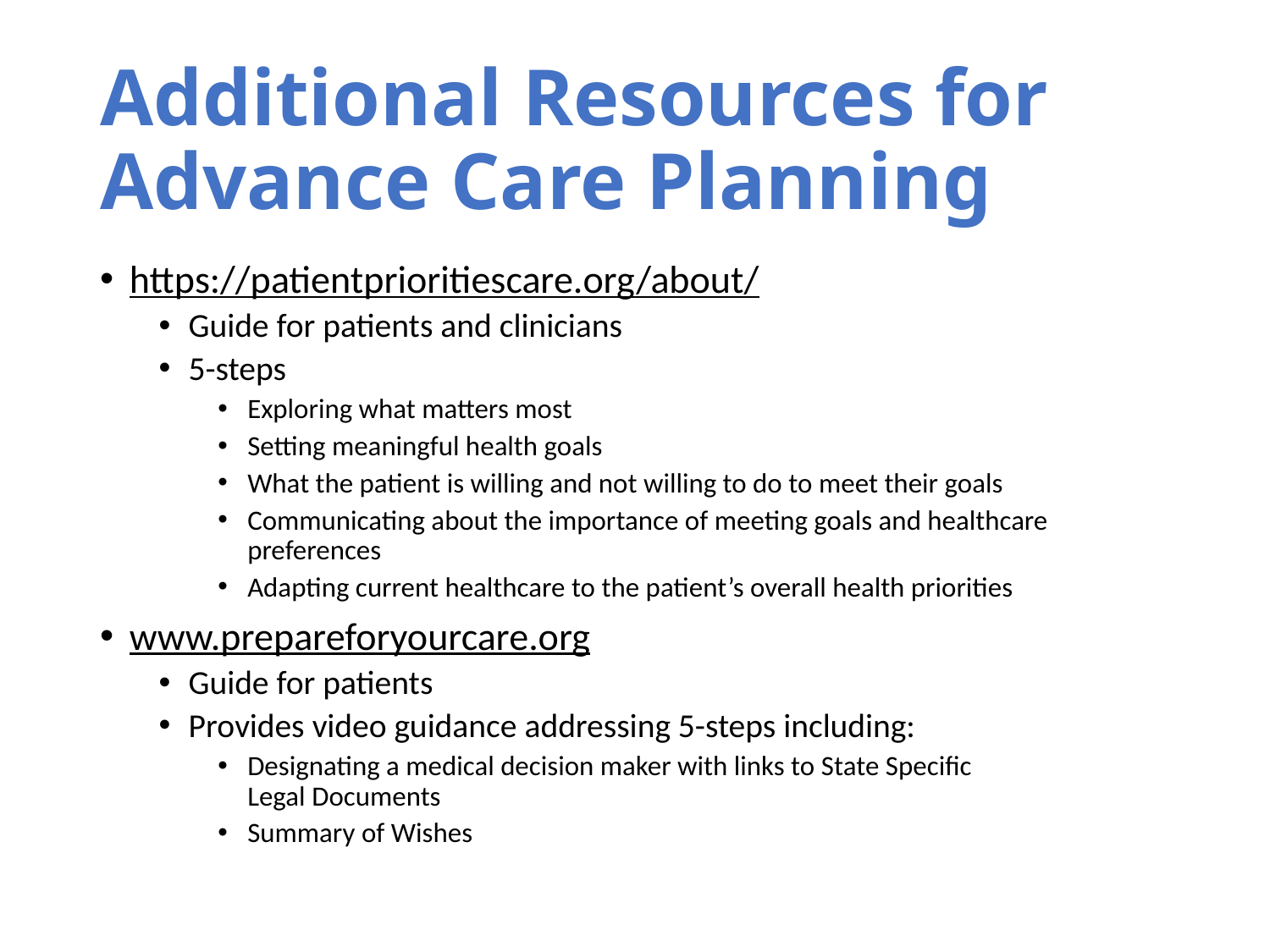

# Additional Resources for Advance Care Planning
https://patientprioritiescare.org/about/
Guide for patients and clinicians
5-steps
Exploring what matters most
Setting meaningful health goals
What the patient is willing and not willing to do to meet their goals
Communicating about the importance of meeting goals and healthcare preferences
Adapting current healthcare to the patient’s overall health priorities
www.prepareforyourcare.org
Guide for patients
Provides video guidance addressing 5-steps including:
Designating a medical decision maker with links to State Specific Legal Documents
Summary of Wishes

## Slide 36
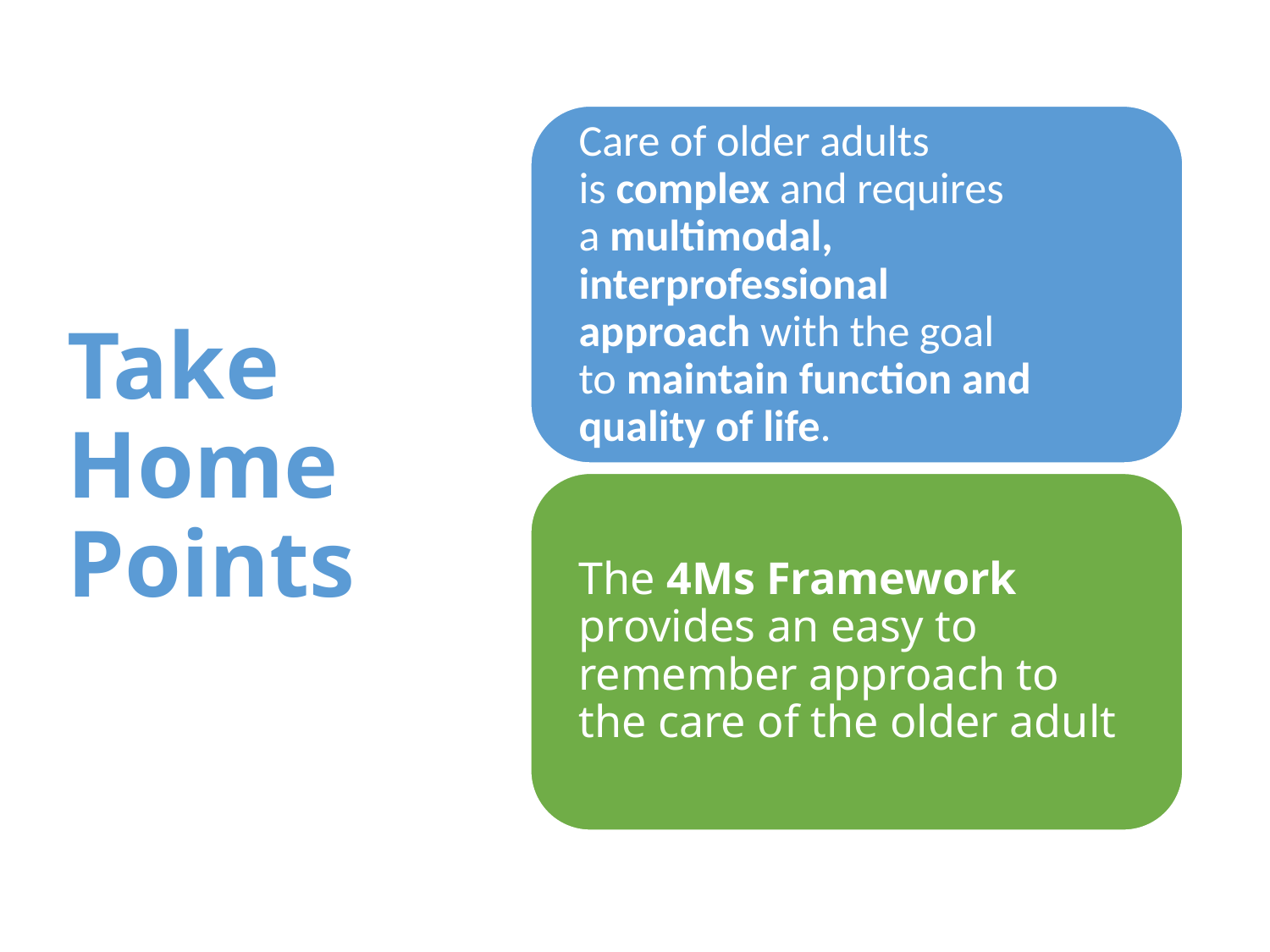

# Take Home Points

## Slide 37
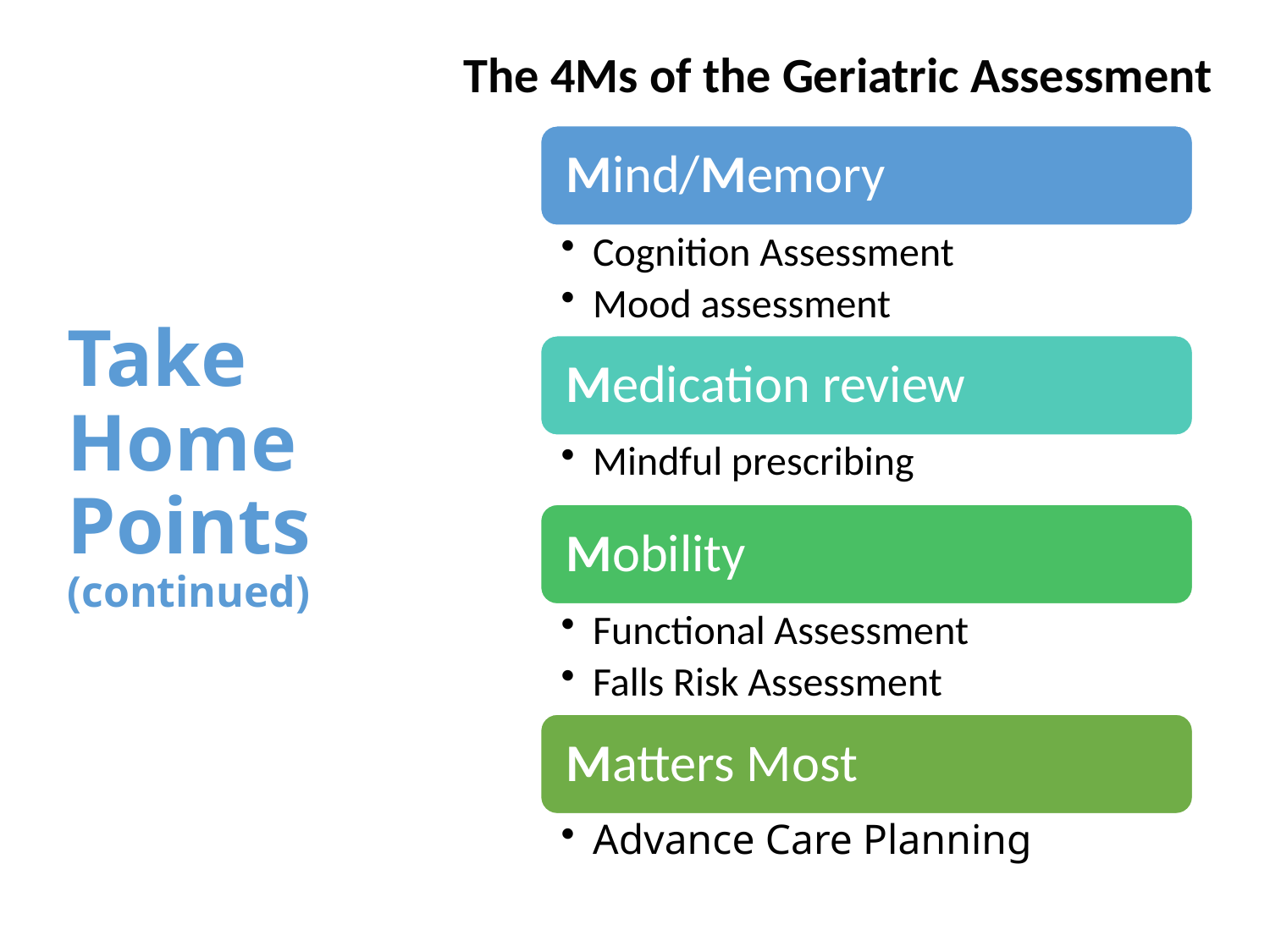

The 4Ms of the Geriatric Assessment
# Take Home Points (continued)
