# Supplementary material for: 4Ms for Early Learners: A Skills-Based Geriatrics Curriculum for Second-Year Medical Students
Source: MedEdPORTAL. 2022 Jun 28;18:11264. doi: 10.15766/mep_2374-8265.11264 (PMC9237204; doi:10.15766/mep_2374-8265.11264)
Supplement: Supplementary file 1 — The 4Ms Approach.pptxFaculty Guide.docxStudent A Handout.docxStudent B Handout.docxStudent C Handout.docxPre- and Postsession Student Surveys.docxLarge-Group Session Evaluation Form.docxGeriatrics SP Case.docxGeriatrics SP Checklist.docx [file mep_2374-8265.11264-s001.zip › B. Faculty Guide.docx]

**Faculty Guide for Facilitating Large Group: The 4Ms Approach to the Care of the Older Adult**

**Recommended pre-work for students to complete prior to session:**

Carlson C, Merel SE, Yukawa M. Geriatric syndromes and geriatric assessment for the generalist. *Med Clin North Am*. 2015;99(2):263-279. doi:10.1016/j.mcna.2014.11.003

- **Slide 1/2: Introduction to Session and Session Outline**
  - Background: This session is designed to provide learners with a context to understand the importance of the geriatric assessment given our growing population of older adults and to provide learners with the skillset necessary to conduct a basic geriatric assessment.
  - Faculty Talking Points
    - We have created an interactive didactic session which will provide students with didactics paired with real-time opportunities to apply the introduced skills.
    - There will be 3 practice opportunities during which you will be working with a group of 3 students. (N.B. If the session is being run virtually, students can be sent to individual “breakout rooms” in which they will practice the scenario).
    - Each student will be assigned the letter A, B, or C and will have access to the handouts labeled with their designated letter.
    - Students will each have the opportunity to play the roles of clinician, patient, and observer during the 3 scenarios, respectively.
    - The handouts will indicate the role the student is playing in each scenario.
    - When playing the role of observer, we encourage students to follow the debrief questions provided (Ask the clinician how did that go? What did you do effectively? What did you find challenging?)
      - These debrief questions will promote self-reflection and the provision of learner-centered feedback.
- **Slide 3: Goals and Objectives**
  - Background: The 4M approach is modeled after Dr. Tinetti’s “The Geriatric 5 M’s” (Tinetti M, Huang A, Molnar F. The Geriatric 5 M’s: A New Way of Communicating What We Do. J Am Geriatr Soc. 2017 Sep;65(9):2115). We have chosen not to introduce the concept of multi-morbidity for students who have not yet experienced caring for complex patients in an inpatient setting.
  - Faculty Talking Points: Review goals and learning objectives with the group, highlighting the 4M’s model as a means of creating an easy to remember framework for conducting a basic geriatric assessment
  - Goals
    - Gain the comfort, knowledge, and skills necessary for conducting a geriatric assessment
    - Apply the 4Ms framework to complete a Geriatric Assessment during a clinical encounter with an older adult
  - Learning Objectives

*By the end of the session the learner will be able to:*

- - - Describe the importance of the geriatric assessment in the care of older adults.
    - Describe how to conduct a geriatric assessment using the 4Ms Framework: Mind/Memory, Medications, Mobility, Matters Most.
    - Apply the skills learned to patient scenarios.
- **Slide 4: Geriatric Medicine**
  - Faculty Talking Points:
    - Questions for facilitation – What experiences have you had so far in your training caring for older adults? What specific challenges have you noted in older adults seeking medical care?
- **Slide 5: The Aging of the United States Population**
  - Faculty Talking Points:
    - With the aging of the “baby-boomer” generation, the population of Americans over the age of 65 is rising exponentially
    - With the aging of the United States population, we need physicians who have the skills to care for the unique challenges faced as we age
- **Slide 6: Impact of Chronic Illness**
  - Faculty Talking Points:
    - With Increasing age, comes diseases such as cardiovascular diseases, cancers, respiratory illness, etc. Older adults are more likely to experience multiple chronic diseases.
    - The presence of geriatric syndromes (cognitive impairment, falls, incontinence, vision, or hearing impairment, etc.) are highly associated with dependency in activities of daily living
    - Physicians must have the knowledge, skills, and ability to work with interprofessional teams to address the needs of older adults and work to help maintaining independence
    - The skills we are going to learn and practice today are applicable to patients across the age spectrum who are living with complex and chronic diseases.
- **Slide 7: Case Vignette #1**
  - Invite a student to read the clinical vignette aloud
  - Faculty Talking Points:
    - Questions for facilitation – What could be going on here? How will you approach the patient and her son?
    - Points for consideration – How will you introduce the idea of a memory/cognitive screen?
    - Suggested “manufactured gate” to introduce a cognitive screen, to normalize and to ask permission
      - *“As we age some people notice that they experience a change in their memory. As part of my standard practice with all my patients, I conduct a memory screen. Would that be ok with you?”*
- **Slide 8**: When to consider memory assessment
  - Faculty Talking Points
    - When to consider memory assessment
      - Use this screening during annual visit to get baseline on patients >65 for both mood and memory.
      - If concerns are brought up by family, or the physician is concerned (e.g., Repetitive comments or questions)
      - Also used to monitor known diagnosis and assess progression
- **Slide 9:** Mind/Memory/Mood Assessment Tools
  - Faculty Talking Points
    - Multiple screening tools available for both memory and mood
      - Choice of tool will vary with location of practice, time available and degree of concern for memory loss.
      - For Memory
        - The Mini-Cog is a shorter screening tool that must be followed up with an assessment tool
        - The Mini-Mental State Exam (MMSE) and Montreal Cognitive Assessment (MoCA) are both assessment tools
- **Slide 10:** Mini-Mental State Exam (faculty should insert cognitive screening tool most readily available in their health system or practice setting, with which they are most familiar, and for which they have permission to use)
  - Faculty Talking Points
    - For today’s practice, we have provided you with a copy of the MMSE [INSERT COGNITIVE SCREENING TOOL BEING USED]
    - The MMSE is a proprietary screening tool
- **Slide 11**: Pseudodementia
  - - Depression Screening should also be considered as Mood assessment is a part of the Mind/Memory evaluation
      - Pseudodementia – mood can impact cognitive functioning. Patients with severe, untreated depression may present as a cognitive impairment which will resolve with appropriate treatment of the underlying mood disorder.
      - Depression screen can be completed before or after cognitive screens; if a cognitive assessment score is normal or does not fully correlate with the clinical picture, but a mood assessment score is abnormal this should broaden differential to include pseudodementia.
      - If a patient is in the hospital, their mental status could be affected by comorbidities and acute illness, resulting in delirium, in which case the MMSE (Mini Mental Status Exam) would not be appropriate and the Confusion Assessment Method (CAM)
- **Slide 12:** Case Vignette #1 BREAKOUT SESSION
  - Faculty Talking Points –
    - Now we return to our case vignette
    - Students will be dividing up into groups of 3, each of you will assign yourself the letter A, B, or C.
    - There are specific handouts available for students A, B, and C.
    - For this breakout Student A will play the role of the Clinician, Student B will play the role of the Patient, and Student C will play the role of the observer.
    - Student A’s task is to “Perform a Cognitive Screen Using [INSERT PREFERRED COGNITIVE TOOL HERE].”
    - Student C should facilitate self-reflection and feedback.
  - Provide students with 15 minutes to conduct this role play, allowing time for debrief in the triad.
- **Slide 13:** Case Vignette #1 BREAKOUT SESSION DEBRIEF
  - This is an opportunity for the students to reflect on the experience of completing the cognitive screen.
  - Faculty Talking Points –
    - *How did that go for the students playing the role of the clinician?*
    - *How did that go for the students playing the role of the patient?*
    - *Was the perception of the patient elicited?*
    - *How was this handled during the encounter?*
    - *What did the observers see that their clinicians did particularly effectively?*
    - *What questions do you still have about conducting a cognitive screen in an older adult?*
- **Slide 14:** Summary Mind/Memory Assessment
  - Highlight key points on slide
- **Slide 15**: **Case Vignette #2**
  - Invite a student to read the clinical vignette aloud
  - Faculty Talking Points:
    - Questions for facilitation – *What could be going on here? What else would you like to know?*
- **Slide 16: Case Vignette #2 continued – Medication List**
  - Invite another student to read
  - Faculty Talking Points:
    - Questions for facilitation – Invite observations about this medication list
      - *What information is missing here? (adherence)*
      - *What has happened since she was last seen that may have impacted her medication list?*
      - *How can you go about gathering additional information about these hypotheses?*
- **Slide 17:** Medication Assessment
  - Faculty Talking Points
    - Review definition of medication reconciliation
    - Discuss importance of medication reconciliation at transitions of care
    - Discuss benefits of reviewing actual pill bottles at each visit
- **Slide 18:** Pharmacokinetic Changes with Aging
  - Faculty Talking Points
    - As we age there are numerous changes that impact the pharmacokinetics of medications that predispose older adults to medication side effects.
    - Due to these changes in how older adults process medications in their body, clinicians typically start at lower doses and increase slowly to monitor for such side effects.
- **Slide 19:** Medication Prescribing Cascade
  - Faculty Talking Points (text on this slide may be replaced with image from the following reference: Rochon PA, Gurwitz JH. Optimizing drug treatment for elderly people: the prescribing cascade. BMJ. 1997 Oct 25;315(7115):1096-9. – Figure 1)
    - When caring for patients, especially older patients, one must be careful to think about medications as a cause for symptoms experienced by a patient; when this is not considered, symptoms are often thought to be a new entity. In response, patients are given new medications to treat symptoms resulting in a medication prescribing cascade
    - Faculty can provide examples of prescribing cascade:
      - NSAID/aspirin used then proton pump inhibitors added for gastritis
      - Steroid given then when sugars rise, patients are started on diabetes medications that are not stopped when the steroids stop
      - Metformin causes nausea, patients are then prescribed antiemetics
      - Amlodipine started for hypertension, results in pedal edema for which furosemide is started. Furosemide causes urinary urgency and incontinence, for which oxybutynin is started. Oxybutynin causes constipation for which MiraLAX is started....
- **Slide 20:** Case Vignette #2 BREAKOUT SESSION
  - Faculty Talking Points –
    - Now we return to our breakout session for case vignette #2
    - For this breakout Student B will play the role of the Clinician, Student C will play the role of the Patient, and Student A will play the role of the observer
    - Student B’s task is to “Perform a Medication Reconciliation for this patient”
    - Student C should facilitate self-reflection and feedback:
  - Provide students with 10-12 minutes to conduct this role play, allowing time for debrief in the triad
  - Case Vignette #2 BREAKOUT SESSION DEBRIEF
    - This is an opportunity for the students to reflect on the experience of completing the medication assessment
  - Faculty Talking Points –
    - *How did that go for the students playing the role of the clinician?*
    - *How did that go for the students playing the role of the patient?*
    - *Was the perception of the patient elicited?*
    - *How was this handled during the encounter?*
    - *What did the observers see that their clinicians did particularly effectively?*
    - *What questions do you still have about conducting a medication assessment in an older adult?*
- **Slide 21:** Summary: Medication Assessment
  - Faculty Talking Points –
    - Importance of reviewing medications and potential changes at every encounter; including over the counter medications as these too could carry significant side effects/interact with prescription medications (In this case, the focus was on a patient after hospitalization, but ask students to consider how many specialists patients may see and how many changes in medications can occur with any visit).
    - If possible, have patients bring actual medication bottles to the visit to review in addition to having a list
    - Reminder to think about polypharmacy (not only number of meds but also dosing of meds-some meds may be multiple times per day and cause increased pill burden), medication prescribing cascade, changes in pharmacokinetics with age, as these all can help explain symptoms/issues patient is having
    - Discuss Beers criteria: this is a list of potentially inappropriate medications in the older adult, due to common adverse events, toxicity, pharmacodynamics (long-acting medications), CNS effects and fall risk, anticholinergic side effects --*does not mean you cannot use the medications on the list but use with caution.
    - Discuss “de-prescribing” --constant re-evaluation of medications and if still necessary. One key pearl: every medication should be linked to a problem, but not every problem needs medication. In addition, not all medications are intended for forever use.
- **Slide 22:** Case Vignette #3
  - Invite a student to read the clinical vignette aloud
  - Faculty Talking Points:
    - Questions for facilitation – *What do we know so far that could have contributed to the patient’s fall? What else would you like to know?*
- **Slide 23:** Falls in the Older Adult
  - Faculty Talking Points:
    - Falls are a leading cause of injury and death in older patients
    - Older adults are at increased risk of falls due to factors such as issues with mobility related to varying causes including osteoarthritis/neurologic diseases/sequelae from other chronic illnesses
- **Slide 24:** Activities of Daily Living and Instrumental Activities of Daily Living
  - Faculty Talking Points:
    - Assessment of ADLs (Activities of Daily Living) and IADLs (Instrumental Activities of Daily Living) help to assess what a patient can and cannot do on their own; how dependent a patient may be on others
    - Not its own “M” but it is an important assessment that affects all 4M’s within the geriatric assessment and can affect your plan—determines degree of assistance/help/services patient may need.
- **Slide 25:** Mobility Assessment
  - Faculty Talking Points:
    - Mobility and falls are topics that should be addressed at least annually (Centers for Medicare and Medicaid Services (CMS) requirement)
    - Patients may not offer this information if not asked so it should be a routine question during the geriatric assessment: Have you fallen in the past year? If yes, how many falls have you had? Have you sustained an injury with the fall?
    - The history of a prior fall is a risk factor for a future fall
    - It is also helpful to ask if a patient is afraid of falling
- **Slide 26:** Fall History
  - Faculty Talking Points:
    - The following history is useful for evaluation of a fall in any patient, independent of age.
    - Discussion that this history should be comprehensive including details about the fall but also thinking about the causes of the fall:
      - Patients should be asked about symptoms before fall
      - Did they lose consciousness with fall, or do they recall the entire event?
      - Did they take any medications prior to falling?
      - Do they drink alcohol or use other substances?
      - Ask about their environment: lighting/rugs/cords on floor
      - Adherence with assistive devices
- **Slide 27:** Case Vignette #3 BREAKOUT SESSION
  - Faculty Talking Points –
    - Now we return to our breakout session for case vignette #3
    - For this breakout Student C will play the role of the Clinician, Student A will play the role of the Patient, and Student B will play the role of the observer
    - Student C’s task is to “Conduct a mobility assessment/fall history and Assess the patient’s ADLs and IADLs”
    - Student B should facilitate self-reflection and feedback.
  - Provide students with 15 minutes to conduct this role play, allowing time for debrief in the triad
  - Case Vignette #2 BREAKOUT SESSION DEBRIEF
    - This is an opportunity for the students to reflect on the experience of completing the mobility assessment and assessing ADLs and IADLs
  - Faculty Talking Points –
    - *How did that go for the students playing the role of the clinician?*
    - *How did that go for the students playing the role of the patient?*
    - *Was the perception of the patient elicited?*
    - *How was this handled during the encounter?*
    - *What did the observer see that their clinicians did particularly effectively?*
    - *What questions do you still have about conducting a fall/mobility assessment in an older adult?*
- **Slide 28:** Debrief of Mobility Assessment
  - Faculty Talking Points – Key points to consider for our patient –
    - Mechanism of fall - why and how they fell, mechanical? Loss of consciousness? Preceding Symptoms?
    - Medication as a falls risk - think about high-risk medications the patient is on and in the setting of their chronic illnesses (e.g., in this case patient is on benzodiazepines for anxiety, insulin for diabetes, or a beta blocker for rate control for Atrial Fibrillation)
    - Consider how you will approach your history and physical exam to think about their chronic illnesses that could contribute
      - General Appearance; Vitals (Blood Pressure or Pulse derangements?)
      - HEENT/Neuro
        - Vision
        - Any seizure history?
      - Cardiac
        - Chest pain, palpitations, lightheadedness before event?
      - Neuro
        - Monofilament to Assess proprioception
        - Any tremor?
      - MSK
        - Mechanical, did patient recall tripping?
        - Location of bruising
        - Gait assessment, shuffling gait?
- **Slide 29:** Mobility Assessment – Physical Exam Component
  - Faculty Talking Points
    - Most important part of the physical examination when assessing for falls risk is the musculoskeletal examination including assessment of postural stability
    - Timed “Get up and Go”
      - A patient is observed getting up from a seated position, walking 10 feet (3 meters), turning around, and returning to the chair.
      - During this assessment, the patient is timed. If it takes 12 or more seconds to complete, the patient is at higher risk of falling.
      - In addition, one can observe for proximal weakness as indicated by difficulty getting up from chair without use of arms, one can observe gait abnormalities such as shuffling gait.
      - Not as useful in healthy community-dwelling person
    - Can discuss next steps/recommendations for falls: physical therapy, fall prevention classes, Yoga, Tai Chi, home safety evaluations and recommendations to reduce cords/loose rugs, proper shoe wear, recommendations for assistive device use such as cane and walkers.
- **Slide 30:** Case Vignette #4
  - Invite a student to read the clinical vignette aloud
  - Faculty Talking Points:
    - Questions for facilitation – *What are some observations you might make about this case? Any concerns?*
      - Patient presenting alone, lives alone
      - 2 Recent falls
      - Memory concerns
- **Slide 31:** Comprehensive Geriatric Assessment
  - Faculty Talking Points
    - Opportunity to recap “3Ms” that have been covered so far
      - Memory/Mood
      - Medications
        - What medications is he on for his hypertension and hypercholesterolemia?
        - How might these medications be contributing to his falls or memory concern?
      - Mobility
    - If there are no acute concerns, we must also consider the 4^th^ M – Matters Most
      - Has the patient named a health care proxy?
        - Important for ALL patients, increasingly important in that this patient is demonstrating increasing frailty
      - Have there been discussions with the patient about his goals, wishes, and concerns about his health and his independence

**[N.B. As laws regarding advance care planning vary by state, slides 32-34 should be edited to conform with state and local laws]**

- **Slide 32:** What Matters Most/ Advance Care Planning
  - Faculty Talking Points – Opportunity to define terms that are often confused: living will, health care proxy, Advance Care Plan, and differentiate advance car planning from medical orders
- **Slide 33:** Medical Orders for Life Sustaining Treatment (MOLST)
  - Faculty Talking Points –
    - MOLST form developed for patients in the last year of life, particularly those who live in long term care facilities or are frequently changing sites of care e.g., home to hospital to long term care facility, etc.
    - Provides information re: patient wishes on 6 specific medical interventions
- **Slide 34:** The 5 Wishes
  - Faculty Talking Points –
    - Comprehensive Advance Care plan which includes designation of surrogate decision maker and overall wishes and hopes pertaining to end of life treatment, both medical and social
    - Meets legal requirements in 44 states
    - Additional resources for advance care planning
- **Slide 35:** Additional Resources for Advance Care Planning
  - Faculty Talking Points-
    - Patient Priorities care includes support for both patients and clinicians to explore what matters most to patients to best align treatment planning with goals
    - Prepare for your care is a user-friendly patient-facing resource which includes direct links to the legal documentation required in all U.S. states
- **Slide 36:** Take Home Points
  - Faculty Talking Points – Reiterate the importance of all clinicians gaining comfort in the care of older adults
- **Slide 37:** Take Home Points
  - Faculty Talking Points – opportunity to review 4 M’s as an easy to remember framework for conducting the Geriatric Assessment
