# Supplementary material for: 4Ms for Early Learners: A Skills-Based Geriatrics Curriculum for Second-Year Medical Students
Source: MedEdPORTAL. 2022 Jun 28;18:11264. doi: 10.15766/mep_2374-8265.11264 (PMC9237204; doi:10.15766/mep_2374-8265.11264)
Supplement: Supplementary file 1 — The 4Ms Approach.pptxFaculty Guide.docxStudent A Handout.docxStudent B Handout.docxStudent C Handout.docxPre- and Postsession Student Surveys.docxLarge-Group Session Evaluation Form.docxGeriatrics SP Case.docxGeriatrics SP Checklist.docx [file mep_2374-8265.11264-s001.zip › C. Student A Handout.docx]

**The 4Ms Approach to the Aging Patient Session – Handout for Student A**

**We will be working in small groups for skills-based practice throughout this session. You will be working in groups of 3, each student should be assigned a letter, A, B or C. You will be reviewing each case when instructed to do so by the faculty.**

**Student Roles for each case:**

**Case 1: (page 2-3)**

**Student A – Clinician**

Student B – Patient

Student C – Observer

**Case 2: (page 4)**

**Student A – Observer**

Student B – Clinician

Student C – Patient

**Case 3: (page 5)**

**Student A – Patient**

Student B – Observer

Student C – Clinician

**Case 1**

**Student A – Clinician**

Julia Cortes is a 78-year-old woman presenting to establish care accompanied by her son; she has not seen a physician in over a year.

Past medical history: Hypertension, Hyperlipidemia, and Depression.

Son expresses concern about her missing her medications and not paying her bills on time.

**You are to:**

- **Perform a Cognitive Screen Using a Screening Tool**

**INSERT COGNITIVE SCREENING TOOL HERE**

**Case 2**

**Student A – Observer**

Debrief Questions:

- After the conclusion of the role play, ask the clinician:
  - How did that go?
  - What did you do effectively?
  - What did you find challenging?

Potential questions for discussion:

- Did the clinician ask the patient to share how she has been taking her medications? (Starting with an open-ended question)
- Did the clinician review the patient’s medication list from the Discharge Summary and compare to the last visit list?
- Did the clinician review the list of what was brought to the visit to assess for discrepancies from the intended discharge list?

**Case 3**

**Student A – Patient**

You are Catherine James, an 82-year-old woman with history of anxiety, osteoporosis, type 2 diabetes mellitus, spinal stenosis, and atrial fibrillation coming in to establish care.

You were hospitalized earlier this month after a fall. You present with your husband and aide who report that you had a fall last night in the bathroom.

Since discharge home you have been doing fairly well. You live with your husband who is 85 and healthy. You have a home health aide (HHA) who comes in 4 hours per day and helps you with dressing in the morning, housework, and meal prep.

Fall History:

You remember the fall; you did not lose consciousness. You were not using your walker and the lights were off. You believe that this is what contributed to your fall. You do not have throw rugs or cords in your path to the bathroom from your room. You remember feeling dizzy. You did not have any chest pain. You do not check your blood pressure and heart rate at home regularly (but you do have a blood pressure machine).

ADL (Activities of Daily Living)

1. Bathing and showering – Your HHA assists you with showering, you use a shower chair
2. Bowel and bladder management- You are independent
3. Dressing-HHA assists you
4. Eating- You are independent
5. Feeding- You are independent
6. Functional mobility-you have a walker after the last hospitalization for fall but were NOT using it last night
7. Personal device care – you do not have any “devices” other than the walker
8. Personal hygiene and grooming-HHA assists you
9. Sexual activity-you are sexually active with your husband only
10. Sleep and rest-you sleep 6 hours each night
11. Toilet hygiene- You are independent

I-ADL (Instrumental Activities of Daily Living)

1. Care of others-live with husband, he helps care for you and you help him if needed
2. Care of pets-no pets
3. Child rearing-children are grown
4. Communication device use-you have a cell phone and a landline; you do not have a medic alert
5. Community mobility – your husband does the driving, but he does not like to drive at night
6. Financial management-your husband does the bills with oversight from your adult daughter
7. Health management and maintenance
8. Home establishment and management – your husband or son in law takes care of house maintenance
9. Meal preparation and cleanup-HHA helps with meals when there, you and your husband do together when no HHA present
10. Safety procedures – you do not have any alert devices in the home
