# Supplementary material for: 4Ms for Early Learners: A Skills-Based Geriatrics Curriculum for Second-Year Medical Students
Source: MedEdPORTAL. 2022 Jun 28;18:11264. doi: 10.15766/mep_2374-8265.11264 (PMC9237204; doi:10.15766/mep_2374-8265.11264)
Supplement: Supplementary file 1 — The 4Ms Approach.pptxFaculty Guide.docxStudent A Handout.docxStudent B Handout.docxStudent C Handout.docxPre- and Postsession Student Surveys.docxLarge-Group Session Evaluation Form.docxGeriatrics SP Case.docxGeriatrics SP Checklist.docx [file mep_2374-8265.11264-s001.zip › D. Student B Handout.docx]

**The 4Ms Approach to the Care of the Older Adult - Handout for Student B**

**We will be working in small groups for skills-based practice throughout this session. You will be working in groups of 3, each student should be assigned a letter, A, B or C. You will be reviewing each case when instructed to do so by the faculty.**

**Student Roles for each case:**

**Case 1: (pages 2-3)**

Student A – Clinician

**Student B – Patient**

Student C – Observer

**Case 2: (page 4)**

Student A – Observer

**Student B – Clinician**

Student C – Patient

**Case 3: (page 5)**

Student A – Patient

**Student B – Observer**

Student C – Clinician

**Case 1**

**Student B- Patient**

You are Julia Cortes, a 78-year-old woman who presents to establish care accompanied by your son; you have not seen a physician in over a year.

Past medical history: Hypertension, Hyperlipidemia, and Depression

Your son expresses concern about you missing your medications and not paying your bills on time. You are aware that you have been forgetting things occasionally, you are hesitant to admit this as you feel embarrassed and are afraid of what this could mean for your life.

***You will answer the questions to the MMSE (Mini Mental Status Exam)**

**MMSE:**

**Today’s Date: (insert incorrect date, correct year)**

**Where are we: Doctors office, 1^st^ floor, Hempstead, NY Queens County**

**3 objects: repeat all 3 without problem**

**Spell WORLD backwards: you will say: “D-L-R-W”**

**3 object recall: you cannot recall any**

**Naming: name the two objects without a problem (watch, pen)**

**Repeat phrase without problem**

**Follow the 3-step command of taking paper in right hand, folding it in half, and placing it on the floor without problem**

**Read “Close your eyes” and then close your eyes**

**Give the following sentence when asked to write a sentence: “I am having a good day.”**

**Copy the intersecting pentagons without problem**

**INSERT COGNITIVE SCREENING TOOL HERE**

**Case 2:**

**Student B – Clinician**

Maria Clark, an 85-year-old woman with history of bipolar disorder, type 2 diabetes, hypertension, hypercholesterolemia, hypothyroidism, and anxiety being seen today after a recent hospitalization for tremors.

MRI brain was negative for CVA. Tremors were thought to be due to benzodiazepine withdrawal from medication nonadherence.

She presents with her daughter who reports that she thinks she has been missing her medications and that she has had a recent fall.

**You are to:**

- Perform a Medication Reconciliation (note: patient had a fall, but you will not assess that in this case, you will only focus on medications as this could be the underlying issue)

**Medication List from the patient’s last visit:**

Alprazolam 1mg q6hrs as needed
Atorvastatin 20mg bedtime
Lisinopril 20mg daily
Metoprolol Tartrate 50mg TID
Metformin 500mg BID
Ibuprofen 600mg as needed 3 times a day
Pantoprazole 40mg daily

Quetiapine 100mg bedtime

**Medication List from the patient’s Hospital Discharge Summary:**

Simvastatin 20mg bedtime
Lisinopril 20mg daily
Metoprolol Succinate 100mg daily
Metformin 500mg BID
Ibuprofen 600mg as needed 3 times a day
Pantoprazole 40mg daily

Quetiapine 100mg bedtime

**Case 3:**

**Student B – Observer**

Debrief Questions:

- After the conclusion of the role play, ask the clinician:
  - How did that go?
  - What did you do effectively?
  - What did you find challenging?

Potential questions for discussion:

**Mobility Assessment/Fall Checklist**

Did the clinician ask about:

- Dizziness?
- Chest pain?
- Vision issues?
- Does the patient remember the fall?
- Any loss of consciousness?
- Assistive device use?
- Lighting?
- Throw rugs/cords? Other tripping hazards?

**ADL (Activities of Daily Living)**

- Bathing and showering
- Bowel and bladder management
- Dressing
- Eating
- Feeding
- Functional mobility
- Personal device care
- Personal hygiene and grooming
- Sexual activity
- Sleep and rest
- Toilet hygiene

**I-ADL (Instrumental Activities of Daily Living)**

- Care of others (including selecting and supervising caregivers)
- Care of pets
- Child rearing
- Communication device use
- Community mobility
- Financial management
- Health management and maintenance
- Home establishment and management
- Meal preparation and cleanup
- Safety procedures
