# Supplementary material for: 4Ms for Early Learners: A Skills-Based Geriatrics Curriculum for Second-Year Medical Students
Source: MedEdPORTAL. 2022 Jun 28;18:11264. doi: 10.15766/mep_2374-8265.11264 (PMC9237204; doi:10.15766/mep_2374-8265.11264)
Supplement: Supplementary file 1 — The 4Ms Approach.pptxFaculty Guide.docxStudent A Handout.docxStudent B Handout.docxStudent C Handout.docxPre- and Postsession Student Surveys.docxLarge-Group Session Evaluation Form.docxGeriatrics SP Case.docxGeriatrics SP Checklist.docx [file mep_2374-8265.11264-s001.zip › E. Student C Handout.docx]

**The 4Ms Approach to the Care of the Older Adult - Handout for Student C**

**We will be working in small groups for skills-based practice throughout this session. You will be working in groups of 3, each student should be assigned a letter, A, B or C. You will be reviewing each case when instructed to do so by the faculty.**

**Student Roles for each case:**

**Case 1:** **(pages 2-3)**

Student A – Clinician

Student B – Patient

**Student C – Observer**

**Case 2: (page 4)**

Student A – Observer

Student B – Clinician

**Student C – Patient**

**Case 3: (page 5)**

Student A – Patient

Student B – Observer

**Student C – Clinician**

**Case 1**

**Student C – Observer**

Debrief Questions:

- After the conclusion of the role play, ask the clinician:
  - How did that go?
  - What did you do effectively?
  - What did you find challenging?

Potential questions for discussion:

- How did the clinician establish rapport with the patient?
- Did the clinician introduce the cognitive screening tool with a normalizing statement?

**INSERT COGNITIVE SCREENING TOOL HERE**

**Case 2:**

**Student C – Patient**

You are Maria Clark, an 85-year-old woman with history of bipolar disorder, type 2 diabetes mellitus, hypertension, hyperlipidemia, hypothyroidism, and anxiety seen for a visit after a recent hospitalization for tremors.

In the hospital, they did an MRI of your head which was negative for stroke. The tremors were thought to be due to inconsistently taking your medication, specifically your alprazolam (benzodiazepine withdrawal).

You are coming in because your daughter reports that she thinks you have been missing medications and you had a recent fall. You have been doing your best to take your medication as prescribed by the doctors. You only take the alprazolam when you are feeling very anxious, and you feel like “the doctors seem to be changing the medications all of the time.”

**Medication List as you are currently taking at home:**

Alprazolam 1mg q6hrs prn—you are using two per day when asked
Atorvastatin 20mg bedtime

Simvastatin 20mg bedtime
Lisinopril 20mg daily
Metoprolol Tartrate 50mg TID

Metoprolol succinate 100mg daily
Metformin 500mg BID
Ibuprofen 600mg as needed 3 times a day
Pantoprazole 40mg daily

Quetiapine 100mg—taken every morning

**Case 3:**

**Student C – Clinician**

Catherine James is an 82-year-old woman with history of anxiety, osteoporosis, type 2 diabetes mellitus, spinal stenosis, and atrial fibrillation who is being seen to establish care.

She was recently hospitalized earlier this month after a fall. She presents with her husband and aide who report the patient had a fall last night in the bathroom.

**You are to:**

- Conduct a mobility assessment/fall history
- Assess the patient’s Activities of Daily Living (ADLs) and Instrumental Activities of Daily Living (IADLs)
