# Supplementary material for: 4Ms for Early Learners: A Skills-Based Geriatrics Curriculum for Second-Year Medical Students
Source: MedEdPORTAL. 2022 Jun 28;18:11264. doi: 10.15766/mep_2374-8265.11264 (PMC9237204; doi:10.15766/mep_2374-8265.11264)
Supplement: Supplementary file 1 — The 4Ms Approach.pptxFaculty Guide.docxStudent A Handout.docxStudent B Handout.docxStudent C Handout.docxPre- and Postsession Student Surveys.docxLarge-Group Session Evaluation Form.docxGeriatrics SP Case.docxGeriatrics SP Checklist.docx [file mep_2374-8265.11264-s001.zip › F. Pre- and Postsession Student Surveys.docx]

**The 4Ms Approach to the Care of the Older Adult Pre-Session Survey:**

Please answer the following questions regarding the 4Ms Approach to the Care of the Older Adult. The results will provide feedback to the School of Medicine for updating its curriculum and may be used in medical education research. By filling out this section you consent to the use of this data for medical educational purposes and publications. Responses gathered will **not** be linked to personal information from individual participants.

1. I feel comfortable participating in the care of an older adult
   - Strongly Agree
   - Agree
   - Neither Agree nor Disagree
   - Disagree
   - Strongly Disagree
2. I have sufficient knowledge to be able to perform a Geriatric Assessment with an older adult.
   - Strongly Agree
   - Agree
   - Neither Agree nor Disagree
   - Disagree
   - Strongly Disagree
3. I am interested in learning more about the care of an older adult
   - Strongly Agree
   - Agree
   - Neither Agree nor Disagree
   - Disagree
   - Strongly Disagree
4. My level of interest in pursuing a career in Geriatrics:
   - Very interested
   - Somewhat interested
   - Slightly Interested
   - Not at all interested

6. Please rate your **level of confidence** for each knowledge area and skill:*

| **Area** | **Skill** | **Level of Confidence** | | | | |
| --- | --- | --- | --- | --- | --- | --- |
| 1. Not at all confident 2. Minimally confident 3. Moderately confident 4. Very confident 5. Extremely confident | | | | | | |
| **Mobility** | Identify an older person at higher risk for falls | 1 | 2 | 3 | 4 | 5 |
|  | Assess gait and balance | 1 | 2 | 3 | 4 | 5 |
|  | Assess for risk of falls | 1 | 2 | 3 | 4 | 5 |
| **Medications** | Use BEERS criteria to identify potentially inappropriate medications for older patients | 1 | 2 | 3 | 4 | 5 |
|  | Perform a medication reconciliation | 1 | 2 | 3 | 4 | 5 |
|  | Identify changes in pharmacokinetics in older adults | 1 | 2 | 3 | 4 | 5 |
| **Mind** | Screen an older adult for depression | 1 | 2 | 3 | 4 | 5 |
|  | Screen an older adult for cognitive impairment | 1 | 2 | 3 | 4 | 5 |
| **Matters Most** | Initiate a conversation about Advance Care Planning | 1 | 2 | 3 | 4 | 5 |
|  | Complete a Health Care Proxy | 1 | 2 | 3 | 4 | 5 |
| **Activities of Daily Living** | Assess an older person for Activities of Daily Living | 1 | 2 | 3 | 4 | 5 |
|  | Assess an older person for Instrumental Activities of Daily Living | 1 | 2 | 3 | 4 | 5 |

*Confidence survey adapted from “Preworkshop Evaluation for Learners”. Survey Phillips SC, Hawley CE, Triantafylidis LK, Schwartz AW. Geriatrics 5Ms for Primary Care Workshop. MedEdPORTAL. 2019 Mar 15;15:10814.

**The 4Ms Approach to the Care of the Older Adult Post-Session Survey:**

Please answer the following questions regarding the 4Ms Approach to the Care of the Older Adult. The results will provide feedback to the School of Medicine for updating its curriculum and may be used in medical education research. By filling out this section you consent to the use of this data for medical educational purposes and publications. Responses gathered will **not** be linked to personal information from individual participants.

1. I feel comfortable participating in the care of an older adult.
   - Strongly Agree
   - Agree
   - Neither Agree nor Disagree
   - Disagree
   - Strongly Disagree
2. I have sufficient knowledge to be able to perform a Geriatric Assessment with an older adult.
   - Strongly Agree
   - Agree
   - Neither Agree nor Disagree
   - Disagree
   - Strongly Disagree
3. I am interested in learning more about the care of the older adult.
   - Strongly Agree
   - Agree
   - Neither Agree nor Disagree
   - Disagree
   - Strongly Disagree
4. My level of interest in pursuing a career in Geriatrics:
   - Very interested
   - Somewhat interested
   - Slightly Interested
   - Not at all interested

5. Please rate your **level of confidence** for each knowledge area and skill:*

| **Area** | **Skill** | **Level of Confidence** | | | | |
| --- | --- | --- | --- | --- | --- | --- |
| 1. Not at all confident 2. Minimally confident 3. Moderately confident 4. Very confident 5. Extremely confident | | | | | | |
| **Mobility** | Identify an older person at higher risk for falls | 1 | 2 | 3 | 4 | 5 |
|  | Assess gait and balance | 1 | 2 | 3 | 4 | 5 |
|  | Assess for risk of falls | 1 | 2 | 3 | 4 | 5 |
| **Medications** | Use BEERS criteria to identify potentially inappropriate medications for older patients | 1 | 2 | 3 | 4 | 5 |
|  | Perform a medication reconciliation | 1 | 2 | 3 | 4 | 5 |
|  | Identify changes in pharmacokinetics in older adults | 1 | 2 | 3 | 4 | 5 |
| **Mind** | Screen an older adult for depression | 1 | 2 | 3 | 4 | 5 |
|  | Screen an older adult for cognitive impairment | 1 | 2 | 3 | 4 | 5 |
| **Matters Most** | Initiate a conversation about Advance Care Planning | 1 | 2 | 3 | 4 | 5 |
|  | Complete a Health Care Proxy | 1 | 2 | 3 | 4 | 5 |
| **Activities of Daily Living** | Assess an older person for Activities of Daily Living | 1 | 2 | 3 | 4 | 5 |
|  | Assess an older person for Instrumental Activities of Daily Living | 1 | 2 | 3 | 4 | 5 |

*Confidence survey adapted from “Preworkshop Evaluation for Learners” Survey. Phillips SC, Hawley CE, Triantafylidis LK, Schwartz AW. Geriatrics 5Ms for Primary Care Workshop. MedEdPORTAL. 2019 Mar 15;15:10814.

6. One new thing I learned during today’s session that I didn’t know before:

_____________________________________________________________________________
