# Supplementary material for: 4Ms for Early Learners: A Skills-Based Geriatrics Curriculum for Second-Year Medical Students
Source: MedEdPORTAL. 2022 Jun 28;18:11264. doi: 10.15766/mep_2374-8265.11264 (PMC9237204; doi:10.15766/mep_2374-8265.11264)
Supplement: Supplementary file 1 — The 4Ms Approach.pptxFaculty Guide.docxStudent A Handout.docxStudent B Handout.docxStudent C Handout.docxPre- and Postsession Student Surveys.docxLarge-Group Session Evaluation Form.docxGeriatrics SP Case.docxGeriatrics SP Checklist.docx [file mep_2374-8265.11264-s001.zip › G. Large-Group Session Evaluation Form.docx]

* indicates a mandatory response

Session Evaluation: Large Group Session

|  | n/a (Did not attend) | Strongly Disagree | Disagree | Neither Agree nor Disagree | Agree | Strongly Agree |
| --- | --- | --- | --- | --- | --- | --- |
| *1. The learning objectives and pre-work prepared me for the  session. | 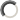 | 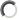 | 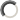 | 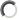 | 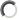 | 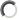 |
| *2. The faculty member fostered active learning (i.e., did not  simply lecture, but asked questions and interacted with students). | 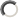 | 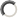 | 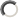 | 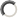 | 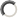 | 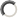 |
| *3. The session appropriately balanced individual facts and  conceptual knowledge as related to the goals of the session. | 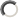 | 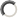 | 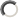 | 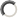 | 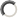 | 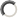 |
| *4. The instructor communicated material at a level that was  appropriate for the learners. | 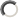 | 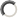 | 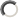 | 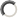 | 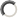 | 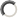 |
| *5. The instructor was well organized, delivering content in a logical progression (e.g., slides support appropriate pacing and  transitions between subjects). | 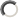 | 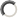 | 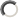 | 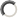 | 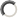 | 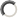 |
| *6. The tone or atmosphere of the session enabled learners to comfortably identify and address their concerns and/or  limitations. | 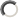 | 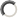 | 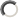 | 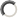 | 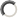 | 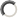 |
| *7. The session appropriately complemented/enhanced my  learning relative to this week's theme. | 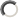 | 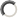 | 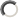 | 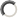 | 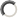 | 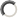 |

*Describe the aspects of this session that worked well and what could be improved. Be constructive and specific.

Page 1
