# Supplementary material for: 4Ms for Early Learners: A Skills-Based Geriatrics Curriculum for Second-Year Medical Students
Source: MedEdPORTAL. 2022 Jun 28;18:11264. doi: 10.15766/mep_2374-8265.11264 (PMC9237204; doi:10.15766/mep_2374-8265.11264)
Supplement: Supplementary file 1 — The 4Ms Approach.pptxFaculty Guide.docxStudent A Handout.docxStudent B Handout.docxStudent C Handout.docxPre- and Postsession Student Surveys.docxLarge-Group Session Evaluation Form.docxGeriatrics SP Case.docxGeriatrics SP Checklist.docx [file mep_2374-8265.11264-s001.zip › H. Geriatrics SP Case.docx]

**Appendix H: Geriatrics Standardized Patient Case Training Notes**

Date: March 19, 2021

Primary Case Author: Gabrielle R. Goldberg, MD

Secondary Case Author: Janice T. John, DO, MS, MPH

Standardized Patient Educator: Theresa Wannamaker, MSEd

Name of Case: The Geriatric Assessment

Name of educational and/or assessment activity: Geriatric Assessment

Patient Name: Julianna/Julian Heras

Chief Concern: “I was in the ER a couple of weeks ago.  They gave me the names of a few geriatricians for follow-up.”

Domains: Check all that apply

1. Professionalism x
2. Communication and Interpersonal skills x
3. Medical History x
4. Physical exam
5. Shared Decision Making
6. Patient Education
7. Clinical Reasoning
8. Documentation
9. Handoff
10. Presentation
11. Other:

Type and level of learner: Medical Student (developed for 2^nd^ year student, can be used for 3^rd^ and 4^th^ years as well)

Case Objectives: please list specific objectives for each of the domains you have checked above:

The medical student should be able to:

1. Gather a medical history
2. Demonstrate core communication skills, including empathy
3. Complete a geriatric assessment using the 4M’s framework: Mind/Memory, Medications, Mobility, Matters Most.
4. Introduce screening tools used with normalizing statements

Additional Tools Needed: Cognitive Screening Tool (e.g., Mini-Mental Status Examination), Depression screening Tool (e.g., PHQ-9)

| SETTING: | Outpatient Geriatric Practice |
| --- | --- |
| PATIENT PROFILE: | |
| Age range | > 65 (SP should be able to portray an older adult patient) |
| Religious/spiritual background | All may be used |
| Sex (e.g., male, female, intersex, transwoman, transman) | All may be used; case is written as a female but can be easily adjusted |
| Sexual Orientation (e.g., heterosexual, lesbian, gay, bisexual, pansexual, queer, asexual) | All may be used |
| Gender expression (e.g., man, woman, gender queer) | All may be used |
| Race/ethnicity: | All may be used |
| Physical description (e.g., BMI, height range) | All may be used |
| Physical limitations | Fully independent with Activities of Daily Living |
| Patient appearance (e.g., disheveled, hospital gown, business casual, casual) | Business Casual |
| Moulage + location (e.g., none, bruises, scars, body piercing, tattoos) | n/a |
| Affect (e.g., pleasant, cooperative) | Pleasant and cooperative |
| Family group (e.g., who is family, who they live with) | Jack, your spouse of 38 years died 6 years ago in a car accident, now you live alone and are proud of your independence. You have been dating a man named Philip for the last year and a half, it has been nice to have companionship and passion reintroduced in your life.  Two adult children – son Gabe is 42 lives nearby with his wife and their 2 children ages 15 and 13, daughter Rose is 45, is not married, has no children and lives across the country. |
| Education | Completed a Bachelor of Nursing Degree |
| Level of health literacy | As a nurse, proficient or higher level of health literacy |
| Employment, if any - present and past, noting any current stresses | You worked as an inpatient pediatric nurse for many years, more recently you have been working as a school nurse (last several years).   You feel pressured to retire though you enjoy the job thoroughly. You love working with kids, having evenings and weekends off. You start to feel ill when you think of how the school district is trying to force you to retire. |
| Home/homeless - type of dwelling, number of stories, owned or rented | Live in a small ranch house with no stairs which you bought soon after your spouse died. |
| Financial situation- any current stresses | No current financial stressors, you have retirement savings from your prior job as well as money from her spouse’s life insurance. |
| Insurance Status (e.g., un/under/insured, public/private, HMO/PPO) | Recently enrolled in Medicare |
| Habits (i.e., diet, exercise, caffeine, smoking, alcohol, drugs) | Diet: No restrictions, though you rarely eat red meat  Exercise: your exercise regimen includes walking 3-4 miles a few times a week with friends when the weather isn’t too cold.  You recently started attending a yoga class through the local community center, though your exercise was put on hold after the fall because of the soreness and bruising on your bottom.  Caffeine: green tea 1-2 cups a day.  Smoking: never  Alcohol: occasionally 1-2 glasses of wine when you need to relax, maximum of 1-2 times a week  Recreational Drugs: none, smoked marijuana in college and occasionally in her early 20s, no drug use since |
| Activities (i.e., hobbies, sports, clubs, friends) | Sleep: Lately you wake up several times over the night, but able to fall back to sleep without too much trouble, not related to any symptoms of trouble breathing or needing to urinate. Go to bed around 10ish and wake up at 5:30-6.  If asked, you think this is something that just happens as one ages.  Religion/Spirituality: Am learning about meditation and yoga which you have found helpful managing stress, you do not identify with any specific faith communities or religions. |
| Typical day - what is the usual daily routine | Typically wake up around 5:30-6AM  Will often read in bed for 30 minutes-hour  Shower and eat breakfast  Report to work 7:45AM-3:30PM  3-4x per week will meet friends for a walk at the local park or track  6PM – Dinner  Watch TV or read book in the evening  9:30 –10PM Go to sleep |

| CASE INFORMATION | |
| --- | --- |
| Chief Concern: | “I was in the ER a couple of weeks ago.  They gave me the names of a few geriatricians for follow-up.” |
| Additional Concerns: Other, if any, concerns the patient has today (i.e., symptoms, requests, expectations, etc.) that will become part of set agenda. | No additional agenda items |
|  | |
| THE PATIENT STORY: | “Well, it’s embarrassing, I went out to pick up a couple of groceries at the local market that I couldn’t order online. I went later in the evening to avoid crowds. It was dark so I didn’t see a patch of ice and ended up falling pretty hard.  I actually bumped into a friend of mine at the store who saw me fall, as did another person in the parking lot. My friend and the people who saw it happen insisted that I go into the ED to be evaluated.  Luckily, everything checked out and I didn’t have to stay there too long.”    If asked a follow up question as to why a geriatrician: “The ER doctor recommend I follow up with my doctor. My primary care doctor for the last 35 years recently retired, and I figured that if I had to restart a relationship with an MD it should be someone who can see me through as I age.”    Personal Context: You are concerned that if pushed out of your job, you will lose your vibrancy and become “old” further worried by the fact that you have not fallen like that “since you were a kid” and falls seem like an “old person problem” You really want to continue working. You had considered retirement with the pandemic but have felt the district was making safe choices throughout and feel even more comfortable now that you are vaccinated.  Emotional Context: You are upset about the embarrassment of the whole fall and the situation at work which is causing some stress but coping in a healthy way. When work comes up it is clear that you get a bit heated and show your frustration, get a bit choked up if work comes up.  If learner responds with empathy, you will share “There was a recent issue at work when you misplaced an epi-pen for one of the kids with a severe peanut allergy that came into the office complaining of a scratchy throat during lunch.  Thankfully, you found it after only a brief (few minutes) delay, but the child got very nervous, and the parents and the administration made a ‘big deal about it.’” |
| HISTORY OF PRESENT ILLNESS:  You are coming in today for an initial visit with a new doctor. You were recently seen in the emergency room after a fall, and they recommended that you follow up with your primary care doctor. The primary care doctor you had been seeing for many years recently retired. You have chosen to schedule an appointment with a geriatrician as you just enrolled in Medicare are planning of the future. Three weeks ago, you were out grabbing a couple of items at your local grocery store. It was late and dark, and you slipped on some ice. Luckily, you were just bumped and bruised on your right hip, right lower ribs, and right arm thankfully you did not break anything. You did not hit your head and did not lose consciousness. You were sore for several days and took over a week to feel better.  You had to put your exercise routine on hold after the fall because of the soreness and bruising on your bottom but feel like you are almost back to yourself.  You have otherwise been feeling well and have no specific complaints or concerns. | |
| Onset (when; gradual or sudden) | The fall you experienced was completely unexpected and occurred without warning or any preceding symptom. |
| Setting (what was going on or where was patient when symptoms first noticed?) | The fall occurred at a local market |
| Duration (how long) | Fall was about 3 weeks ago |
| Time relationships (frequency, constant or intermittent) | You have not had any falls since that event. |
| Location | You fell forward and on to your right side. |
| Radiation | n/a |
| Quality | n/a |
| Amount | n/a |
| Aggravated by what | n/a |
| Relieved by what | n/a |
| Associated with what | n/a |
| Attitude (what does the patient think is the problem, and how does he/she feel about it) | You think the fall was simply a “freak accident” but feel embarrassed that it happened and concerned that people will perceive you as “old” |
| Overall course | After being seen in the Emergency Room you were sent home and told to follow-up with your primary doctor. As he recently retired, you thought it might make sense to establish care with a geriatrician and the emergency room provided the contact information for this office. |
| Falls Risk Assessment | You have not had any other falls “since you were a kid.”  You do not have any stairs or rugs at home. You live in a small 2-bedroom ranch.  You have hardwood floors throughout the house with carpets in the bedroom, no rugs. You wear your glasses and see your optometrist yearly. If asked, you don’t feel like you have had any issues with balance, dizziness, coordination.  You’ve never used assistive devices (e.g., cane or walker) |
| REVIEW OF SYSTEMS: Significant positives and negatives | |
| No dizziness prior to fall | No change in vision |
| No history of fall | No headaches |
| No loss of consciousness | No weakness |
| No head trauma with fall | Occasional constipation |
| No chest pain prior to fall |  |
| Past medical history |  |
| Medication allergies (Name and reaction) | No known drug allergies |
| Environmental allergies (Name and reaction) | No environmental allergies |
| Illnesses |  |
| Vaccinations | COVID vaccine received 2 doses + booster of Pfizer vaccine |
| Surgeries | None |
| Accidents/ injuries/ trauma | No history of accident/injuries |
| Hospitalization | Only for childbirth – last was 40 years ago – both uncomplicated normal vaginal deliveries |
|  | |
| Inclusive sexual and reproductive history | |
| Sexual practices  Sexual partners  Protection: Use of safer sex practices  Use of birth control if appropriate  Risk of intimate partner violence | Sexually active currently with one partner, vaginal intercourse, if asked: has experienced some vaginal dryness, managing with lubricant for intercourse.  Preference: male  Number of partners, past and present: 1 now. Lifetime - 6 - 5 in early adulthood, husband, and now current partner  Currently active: yes  STD protection: condoms  STI Testing: not anytime recently  No history of intimate partner violence |
| Ob/GYN HISTORY | Age of onset of menses: 14  Age of menopause: 54  Number of pregnancies: 2  Number of live births: 2  Number of miscarriages: 0  Number of abortions: 1 |
| Medications | Prescription/dose/reason  Lisinopril 20 mg daily for high blood pressure x 3 years  Atorvastatin 20 mg daily for high cholesterol x 5 years  If asked: “I am pretty good at remembering these as I leave the bottles by the kitchen windowsill and taking them with breakfast”  Over the counter/dose/reason:  Active Women Multivitamin  Vitamin C  Vitamin D  Glucosamine Chondroitin once a day - for joint health  MiraLAX as needed for constipation  Herbs/supplements/dose/reason: n/a  Other: n/a |
| Immunizations | 1. Tetanus - last 4 years ago 2. Flu - October 3. Hepatitis - immune at last screen 4. Pneumovax - at welcome to Medicare visit 5. HPV - n/a 6. Other - COVID received Pfizer vaccine x 2 |
| Tobacco products:   1. Cigarettes 2. Cigar 3. Pipe 4. Chew 5. E-cigarettes | Never |
| Alcohol   1. Beer 2. Wine 3. Liquor 4. Other | 1-2 glasses of wine “occasionally” when you need to relax, maximum of 1-2 times a week, last drink was 3-4 week ago |
| Drugs   1. Weed 2. Cocaine 3. Heroin 4. Meth 5. Other 6. IV 7. Inhalants 8. Other | Smoked Marijuana in college and intermittently in young adult life, no use x decades |
| Diet (describe) | No restrictions, though only eats red meat occasionally  24h Diet Recall:  Breakfast: bowl of oatmeal with berries  Lunch: Turkey sandwich and apple  Dinner: Grilled Salmon and salad  Snack: small cup of frozen yogurt  Drinks: cup of green tea in the morning, 5-6 glasses of water throughout the day |
| Exercise (describe) | Your usual exercise regimen includes walking 3-4 miles a few times a week (occasionally with friends) when the weather is not too cold. You recently started attending a virtual yoga class through the community center that they are running via Zoom. |
| List any other important social history or information important to this case |  |
| Family history  Mother, Father, Siblings, Grandparents, and other significant findings. | Parents: Father died in his 50s suicide, medication overdose  Mother- died 2 years ago at age 88, secondary to complications after getting pneumonia a short time after she had a stroke.  Siblings: Brother died of colon cancer at age 67. Younger sister is well, just a little high blood pressure. |
| Memory Screening | These are many of the questions students may ask:  (Answers should all be provided as accurately as possible)  What is the year/date/season/month?   Provide correct answers  Where are we? A doctor’s office in *provide correct location*  The student will name 3 objects then ask you to repeat the objects.  The student will ask you to count backward from 100 by sevens OR will ask you to spell WORLD backwards.  The student will ask you to name the 3 things that you were asked to name earlier YOU SHOULD REMEMBER 2 OUT OF THE 3 ITEMS you may feel a little flustered but quickly apologize and explain you are just a little nervous about /distracted by meeting the new doctor.  The student will show you 2 simple objects (e.g., wristwatch, pencil) and ask you to name them.  The student will ask you to Repeat the following: “No ifs, ands or buts”  Follow a 3-stage command such as: Take this paper, fold it in half and give it back to me”  The student will ask you to read and do the following: CLOSE YOUR EYES  The student will ask you to write a sentence  The student will ask you to copy a picture |
| Depression Screening | The student will ask “Over the last 2 weeks, how often have you been bothered by the following problems:   1. Little interest or pleasure in doing things. Your answer: Not at all 2. Feeling down, depressed, or hopeless. Your answer: Not at all   (Students should stop asking questions at this point but if they proceed, the answers should all be “Not at all.”) |
| Activities of Daily Living/Instrumental Activities of Daily Living | You are completely independent and do everything by yourself -- Self-care, household chores, finance, transportation, communication, etc.  However, after your husband died 6 years ago, it did take some getting used to doing the bills, thinking of finances and as this was his domain. |
| Advanced Care Planning | Your spouse was your HCP (Health Care Proxy). Soon after their death, you assigned your son Juan to be your HCP (as he lives closer) with daughter Jasmine as back up. They each have a copy. You forgot to bring it with you today but can mail in a copy or bring it to the next visit. You do not have a living will, but you have had conversations with your kids, and they know your wishes and values. If asked, you value your independence and would never want your life prolonged by artificial means if you could not return to an independent life. |
| PROFESSIONALISM ISSUES OR CHALLENGES: | Empathic Opportunity –  If student responds to comment re: “Starting to feel ill when she thinks of how the school district is trying to force her to retire” with empathy, the SP will share: “There was a recent issue at work when you misplaced an epi pen for one of the kids with severe peanut allergy that came into the office complaining of a scratchy throat during lunch.  Thankfully, you found it after only a brief (few minutes) delay, but the child got very nervous, and the parents and the administration made a ‘big deal about it.’” |

SP Door Chart:

PATIENT NAME: JULIANNA HERAS

DOB: February 4, (update as needed for SP age > 65)

Julianna Heras made an appointment a couple of weeks ago to initiate care in the Geriatrics practice. Dr. James asks you to see the patient and gather her history and conduct a geriatric assessment.

You are to:

1. Complete a thorough geriatric assessment based on what you have learned.
2. Elicit all relevant history
3. Do NOT complete a physical exam
