# Supplementary material for: 4Ms for Early Learners: A Skills-Based Geriatrics Curriculum for Second-Year Medical Students
Source: MedEdPORTAL. 2022 Jun 28;18:11264. doi: 10.15766/mep_2374-8265.11264 (PMC9237204; doi:10.15766/mep_2374-8265.11264)
Supplement: Supplementary file 1 — The 4Ms Approach.pptxFaculty Guide.docxStudent A Handout.docxStudent B Handout.docxStudent C Handout.docxPre- and Postsession Student Surveys.docxLarge-Group Session Evaluation Form.docxGeriatrics SP Case.docxGeriatrics SP Checklist.docx [file mep_2374-8265.11264-s001.zip › I. Geriatrics SP Checklist.docx]

| Standardized Patient Checklist  Patient: Heras  4M’s Geriatrics Assessment Questions  *The following checklist items should be used along with your institution’s standard communication checklist.* |
| --- |
| 1. (MEDICATIONS) Did the student ask about your medications and how you are taking them?   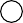 0-No  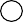 1-The student asked about my medications but did not ask about how I take them.  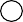 2-Yes |
| 1. (MOBILITY) Did the student ask any question to assess your fall risk (vision, rugs, stairs, prior fall history, use of assistive devices, balance, or coordination)?   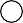 0-No  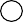 2-Yes |
| 1. (MOBILITY/ADLs) Did the student elicit your ability to perform activities of daily living?   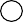 0-No  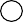 2-Yes |
| 1. (MIND/MEMORY/MOOD) Did the student transition comfortably to the depression screening?   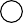 0-No  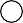 2-Yes |
| 1. (MIND/MEMORY) Did the student use a cognitive screening tool to assess your cognitive function?   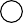 0-No  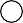 2-Yes |
| 1. (MATTERS MOST) Did the student ask you whether you ever completed a health care proxy or any other questions pertaining to advanced care planning?   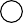 0-No  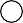 2-Yes |
