# Supplementary material for: 4Ms for Early Learners: A Skills-Based Geriatrics Curriculum for Second-Year Medical Students
Source: MedEdPORTAL. 2022 Jun 28;18:11264. doi: 10.15766/mep_2374-8265.11264 (PMC9237204; doi:10.15766/mep_2374-8265.11264)
Supplement: Supplementary file 1 — The 4Ms Approach.pptxFaculty Guide.docxStudent A Handout.docxStudent B Handout.docxStudent C Handout.docxPre- and Postsession Student Surveys.docxLarge-Group Session Evaluation Form.docxGeriatrics SP Case.docxGeriatrics SP Checklist.docx [file mep_2374-8265.11264-s001.zip › Educational_Summary_Report_11264.pdf]

MedEdPORTAL® The AAMC Journal of Teaching and Learning Resources

Original Publication Open Access

4Ms for Early Learners: A Skills-Based Geriatrics Curriculum for Second-Year Medical Students

Gabrielle R. Goldberg, MD*, Gabriela Solis, MD, Janice T. John, DO, MS, MPH, Doreen M. Olvet, PhD, Kimberly A. Kranz, MD

*Corresponding Author: gabrielle.goldberg@hofstra.edu

Abstract

Introduction: Given the growing population of older adults, it is of utmost importance for all future physicians to be trained in the core skills of conducting geriatric assessment. **Methods:** We designed an interactive, skills-based session introducing core competencies for geriatric assessment for second-year medical students (MS2s). We organized our curriculum for early learners based on the 4Ms framework: mind/memory, medications, mobility, and matters most. The session consisted of brief didactics with integration of real-time skills-based practice. Students completed pre- and postsession surveys to assess their confidence in their knowledge and skills. All students completed a geriatric assessment during a clinical skills encounter as part of a multistation, end-of-course, summative clinical skills examination (CSE). The session was conducted virtually over 2 academic years, and the CSE was conducted virtually in 2020 and in person in 2021. **Results:** One hundred ninety-nine MS2s participated in the session (100 in 2020, 99 in 2021). All students surveyed (33%) reported improved confidence in geriatric knowledge and skills by the end of the session ($ps < .001$). Students were more likely to use a cognitive screening tool, ask about advance care planning, and assess medication adherence on the CSE in 2021 compared to 2020 ($ps < .001$). **Discussion:** We provide an interactive curriculum for MS2s to develop geriatric assessment skills. The curriculum and assessment tools are versatile, can be easily integrated into any medical school curriculum, and can be effectively delivered in person or on a virtual platform.

Keywords
Medication Reconciliation, Falls, Mobility, Case-Based Learning, Clinical Skills Assessment/OSCEs, Geriatrics, Standardized Patient

Educational Objectives

By the end of this activity, learners will be able to:

1. Describe the importance of the geriatric assessment in the care of older adults.
2. Describe how to conduct a geriatric assessment using the 4 Ms framework: mind/memory, medications, mobility, and matters most.
3. Apply the skills learned to patient scenarios.

Introduction

It is predicted that by 2030, all baby boomers will be older than 65[1] and, by 2050, 22% of the United States population will be older than 65.[2] With advanced age, people face more medical challenges, and care becomes increasingly complex. Despite awareness of the growing population of older adults, training of physicians in the principles and practice of geriatrics is not keeping pace.[3] All physicians, regardless of medical specialty, need to be prepared to perform a basic assessment of the unique challenges facing older adults. Introducing geriatrics earlier in medical training has the potential to provide students with greater comfort and knowledge in conducting basic geriatric assessments. Additionally, early exposure may inspire more learners to pursue a career in geriatrics.[4,5]

Citation:
Goldberg GR, Solis G, John JT, Olvet DM, Kranz KA. 4Ms for early learners: a skills-based geriatrics curriculum for second-year medical students. *MedEdPORTAL*. 2022;18:11264.
https://doi.org/10.15766/mep_2374-8265.11264

In response to the initial publication of minimum geriatric competencies for medical students over a decade ago,[6] medical schools developed curricula to prepare students for these competencies. Published curricula for medical students have included case-based discussions,[7-10] standardized patient (SP) cases for use with third-year students,[11-14] and team-based learning activities.[15] The literature on geriatric curricula designed specifically for students in the initial years of medical school is limited. Case-based discussion curricula for second-year medical students (MS2s)[7-10] have been well received by faculty and have

Copyright © 2022 Goldberg et al. This is an open-access publication distributed under the terms of the Creative Commons Attribution license.

demonstrated improvement in student content knowledge but have not been designed to assess psychomotor skills.

In 2017, Tinetti and colleagues introduced the 5Ms—mobility, medications, mind, multicomplexity, and what matters most—an easy-to-remember framework to enhance training efforts in geriatrics.[16] In 2021, the Minimum Competencies in Geriatrics for Medical Students were updated and organized using the 5Ms framework.[17] Geriatric educators have called for the use of this framework to improve the integration of geriatrics content into medical school education.[18]

We developed an interactive curricular session for MS2s to introduce the specialty of geriatrics and the geriatric assessment. We developed our curriculum using the framework of 4Ms: mobility, medications, mind, and matters most. Tinetti's fifth M, multicomplexity, is a more challenging concept better understood by learners with experience practicing in a high-acuity, inpatient setting. The 4Ms model is the framework for the Age-Friendly Health System initiative promoted by the John A. Hartford Foundation and the Institute for Healthcare Improvement.[19]

Integration of skills-based practice with didactic content effectively leads to sustained skills over time.[20] We therefore designed our session using large-group didactics integrated with small-group breakout sessions for case-based role-playing with peer feedback. The didactic segments were designed to be brief and interactive, making use of cognitive learning theory, engaging learners through Socratic questioning, prompting them to share their thought processes, and giving students opportunities to ask questions.[21] We integrated the case-based practice of skills with peer feedback in consideration of experiential learning theory, particularly social constructivist theory, in that we created a social context in which students had the opportunity to apply content as they were learning.[22]

Published geriatrics curricula for medical students have not integrated real-time practice of skills, and there is limited availability of curricula paired with clinical skills assessment. We hypothesized that MS2s who participated in our skills-based geriatrics curriculum would gain confidence in their knowledge and skills, as well as the ability to apply these skills. We therefore designed an SP case for the assessment of students' acquired skills with checklist items aligned with each of the 4Ms. We developed a novel teaching tool using the 4M framework to provide MS2s with an interactive, skills-based curriculum inclusive of skills assessment. The pandemic has proven the need for curricula that are easily transferrable to virtual platforms. Our curriculum and associated SP assessment case can be delivered both in person and virtually.

Methods

Curricular Context

We designed "The 4Ms Approach to the Care of the Older Adult" as a 2-hour session for MS2s at the Donald and Barbara Zucker School of Medicine at Hofstra/Northwell (ZSOM). The session was scheduled during our integrated case-based/problem-based curriculum for neurology and psychiatry. During this course, students learned the basic science content pertaining to pathophysiologic conditions commonly seen in older adults, including dementia, delirium, and stroke; it was therefore deemed to be the appropriate curricular home.

Curricular Design

The goal of the curriculum was for students to gain the comfort, knowledge, and skills necessary for conducting a geriatric assessment. We assigned a prereading to provide students with background and context for the content to be addressed and applied during the session.[23] We developed a PowerPoint (Appendix A) and associated faculty facilitator guide (Appendix B) to guide delivery of the session. Students were provided with access to the PowerPoint as a reference both during and after the session.

We designed the curriculum as a large-group session consisting of interactive didactics, with integration of case-based practice of skills with peer feedback followed by large-group debrief of each of the skills. We created case vignettes with opportunities for students to role-play three scenarios after brief didactics on the topics of mind, medications, and mobility, respectively. The matters most section included only a didactic component as students had participated in a communication session on advance care planning during the prior academic year. We designed the role-plays to allow students to work in triads, providing each student the opportunity to play the role of clinician, patient, and observer during one of the three cases. Student observers were assigned for each role-play to encourage small groups to engage in active reflection on performance.[24] This reflection continued when students returned to the large group to debrief. Our students were familiar with role-plays and the provision of learner-centered peer feedback, as this model was used for our communication curricular sessions.

We designed supplemental student handouts (Appendices C, D, and E) to provide each student with instructions for the roles of clinician, observer, or patient in the three cases. In case 1,

Copyright © 2022 Goldberg et al. This is an open-access publication distributed under the terms of the Creative Commons Attribution license.

Julia Cortes was a 78-year-old woman whose son expressed concerns about her memory; student A played the role of the clinician and was instructed to “perform a cognitive screen using the Mini-Mental State Exam (MMSE).” Students were provided with the MMSE screening tool[25] for use during this role-play as this was the cognitive screening tool used in our institution. In case 2, Maria Clark was an 85-year-old woman with a recent hospitalization; student B played the role of the clinician and was instructed to “perform a medication reconciliation.” In case 3, Catherine James, an 82-year-old woman, presented with a recent fall; student C played the role of the clinician and was instructed to “conduct a mobility assessment/fall history and assess the patient’s activities of daily living and instrumental activities of daily living.”

The initial session (cohort 1) was scheduled for March of 2020. We quickly needed to pivot our curricular plan due to the environmental restrictions imposed by the COVID-19 pandemic. This session was modified to be run virtually as a large-group session. The real-time, case-based role-play was forfeited and replaced with faculty role-modeling of skills, as our initial virtual platform did not accommodate breakout rooms.

The session was conducted again in March of 2021 (cohort 2). During this iteration, we had the opportunity to run the curriculum via a virtual platform that allowed for the use of breakout rooms. This enabled us to return to our original plan for student role-plays. Faculty were not present in breakout rooms as we did not have enough faculty available for the 33 breakout rooms running concurrently. Based on feedback and observations from cohort 1, the large-group content for cohort 2 was also updated to include an expansion of the matters most and advance care planning section, as well as additional content about activities of daily living. Table 1 provides an overview of the curricular components, timing, and educational strategies used for each cohort.

Evaluation Process

Surveys: Students in both cohorts were invited to complete presession and postsession surveys (Appendix F) adapted from prior work by Phillips and colleagues.[26] Students were provided

Table 1. Overview of the Curricular Components, Timing, and Associated Educational Strategies

Curricular Components	Time	Educational Strategies Cohort 1[a]	Educational Strategies Cohort 2[b]
Didactic session			
Interactive large group	20 minutes	Provide background of aging population and introduce 4Ms paradigm<br>Introduce mind/memory and tools used for their assessment	Provide background of aging population and introduce 4Ms paradigm<br>Introduce mind/memory and tools used for their assessment
Case 1: mind/memory	12 minutes	Faculty role-model case 1 (mind/memory): faculty demonstrate patient encounter	Breakout—role-play case 1 (mind/memory): students role-play in triads (student A as clinician, student B as patient, student C as observer)
Interactive large group	20 minutes	Discussion of case 1<br>Introduce prescribing cascade and medication reconciliation	Debrief role-play of case 1<br>Introduce prescribing cascade and medication reconciliation
Case 2: medication	12 minutes	Faculty role-model case 2 (medication): faculty demonstrate patient encounter	Breakout—role-play case 2 (medication): students role-play in triads (student A as observer, student B as clinician, student C as patient)
Interactive large group	20 minutes	Discussion of case 2<br>Introduce falls and mobility assessment	Debrief role-play of case 2<br>Introduce falls and mobility assessment
Case 3	12 minutes	Faculty role-model case 3 (falls and mobility): faculty demonstrate patient encounter	Breakout—role-play case 3 (falls and mobility): students role-play in triads (student A as patient, student B as observer, student C as clinician)
Interactive large group	20 minutes	Discussion of case 3<br>Introduce matters most/advance care planning<br>Summarize and conclude with take-home points	Debrief role-play of case 3<br>Introduce matters most/advance care planning<br>Summarize and conclude with take-home points
Clinical skills assessment			
Standardized patient encounter during end-of-course, summative, four-station clinical skills examination	15 minutes	Virtual/telehealth encounterThe medical student should be able to:<br>1. Gather a medical history<br>2. Demonstrate core communication skills, including empathy<br>3. Complete a geriatric assessment using the 4Ms framework: mind/memory, medications, mobility, matters most<br>4. Introduce screening tools with normalizing statements	In-person encounter at Clinical Skills CenterThe medical student should be able to:<br>1. Gather a medical history<br>2. Demonstrate core communication skills, including empathy<br>3. Complete a geriatric assessment using the 4Ms framework: mind/memory, medications, mobility, matters most<br>4. Introduce screening tools with normalizing statements

[a]The virtual platform did not allow for breakout rooms, so role-play of cases was replaced by faculty role-modeling of cases.
[b]Student role-play was reinstated as the virtual platform used for this iteration allowed for breakout rooms.

Copyright © 2022 Goldberg et al. This is an open-access publication distributed under the terms of the Creative Commons Attribution license.

with QR codes to scan to access these surveys, administered via Qualtrics, in real time at the beginning and end of the session. Students were instructed to create a participant ID using their favorite color and last four digits of their cell number to allow matching of pre- and postsurvey responses. The surveys asked students to self-assess their confidence in their knowledge and skill conducting components of the geriatric assessment. Students were also asked to share their take-home points for the session.

As part of routine operating procedures, a random sampling of a quarter of the students anonymously completed a postsession standardized session evaluation form on our online curriculum management system (Appendix G).

Clinical skills assessment: The ZSOM partnered with the Clinical Skills Center at the Northwell Health Center for Learning and Innovation (CLI) for the administration of clinical skills assessments. The Clinical Skills Center at CLI consisted of 14 rooms designed to resemble outpatient examination rooms. Our students were accustomed to completing an objective structured clinical skills examination (OSCE) with SPs during their examination week at the end of each of the four MS1 and three MS2 courses. We developed an OSCE case to assess students' completion of the geriatric assessment. The case was conducted as a 15-minute clinical encounter with a new patient in an outpatient geriatric practice. This case was one of four encounters conducted during the summative OSCE at the end of the integrated neurology and psychiatry course. Appendix H includes the SP training notes and the door chart provided to the students with instructions for the encounter. Students were also provided with access to a copy of the MMSE.

SPs were recruited by CLI from its regular pool of previously screened and trained SPs. The only specific screening criterion for SP recruitment for this case was the ability to portray a 65-year-old patient. As per CLI standard protocol, SPs were trained to the role, including to the use of checklists to assess student performance. In addition to our standard core communication checklist items, OSCE checklist items were specifically designed to align with the 4Ms session content (Appendix I). Due to restrictions related to the pandemic, cohort 1 completed the OSCE via a telehealth encounter administered by the Clinical Skills Center on the Zoom platform. Cohort 2 completed the OSCE in person at CLI.

The Hofstra University Institutional Review Board deemed the study exempt from ethical review.

Statistical Analysis

We used SPSS Statistics (Version 24.0, IBM) to analyze our data. Descriptive statistics are presented here as the number (and percent) of responses for each category. For the pre-post survey, we used a mixed analysis of variance to determine if there was an effect of group (cohort 1 vs. cohort 2) or of time (pre- vs. postsession) or an interaction between group and time. We used a Mann-Whitney U test to compare session evaluation items between the two cohorts. Individual OSCE checklist items completed by the trained SPs are presented here as the number (and percent) correct. We performed a chi-square analysis to assess group differences for the OSCE performance data. There was one OSCE item that allowed for partial credit. In that instance, we performed post hoc tests with an adjusted standardized residual analysis,[27] and Bonferroni-adjusted p values are presented here. In all cases, a p value $\leq .05$ was considered statistically significant.

Results

Pre- and Postsession Survey

We evaluated students' confidence in their knowledge and skills regarding core geriatric content corresponding to components of the 4M framework on pre- and postsession surveys. In cohort 1, 100 students participated in the session. Survey data for both pre- and postsession were matched for 32 students (32% response rate). The following data were not included in the current analysis: Thirteen students participated in the presession survey but not the postsession survey, and 55 students did not participate in either survey. In cohort 2, 99 students participated in the session. Survey data for both pre- and postsession were matched for 33 students (33% response rate). The following data were not included in the current analysis: Twenty-four students participated in the presession survey but not the postsession survey, four students participated in the postsession survey but not the presession survey, and 38 students did not participate in either survey.

The Figure shows the cumulative percentage of student responses for each survey item before and after participating in session. On all items, there was no significant difference between groups and no interaction between group and year (all $ps > .05$), but both groups showed substantial improvement over time (all $ps < .001$).

We received 32 narrative responses from cohort 1 and 33 narrative responses from cohort 2 to the open-ended question eliciting their take-home points. The students' self-identified

Copyright © 2022 Goldberg et al. This is an open-access publication distributed under the terms of the Creative Commons Attribution license.

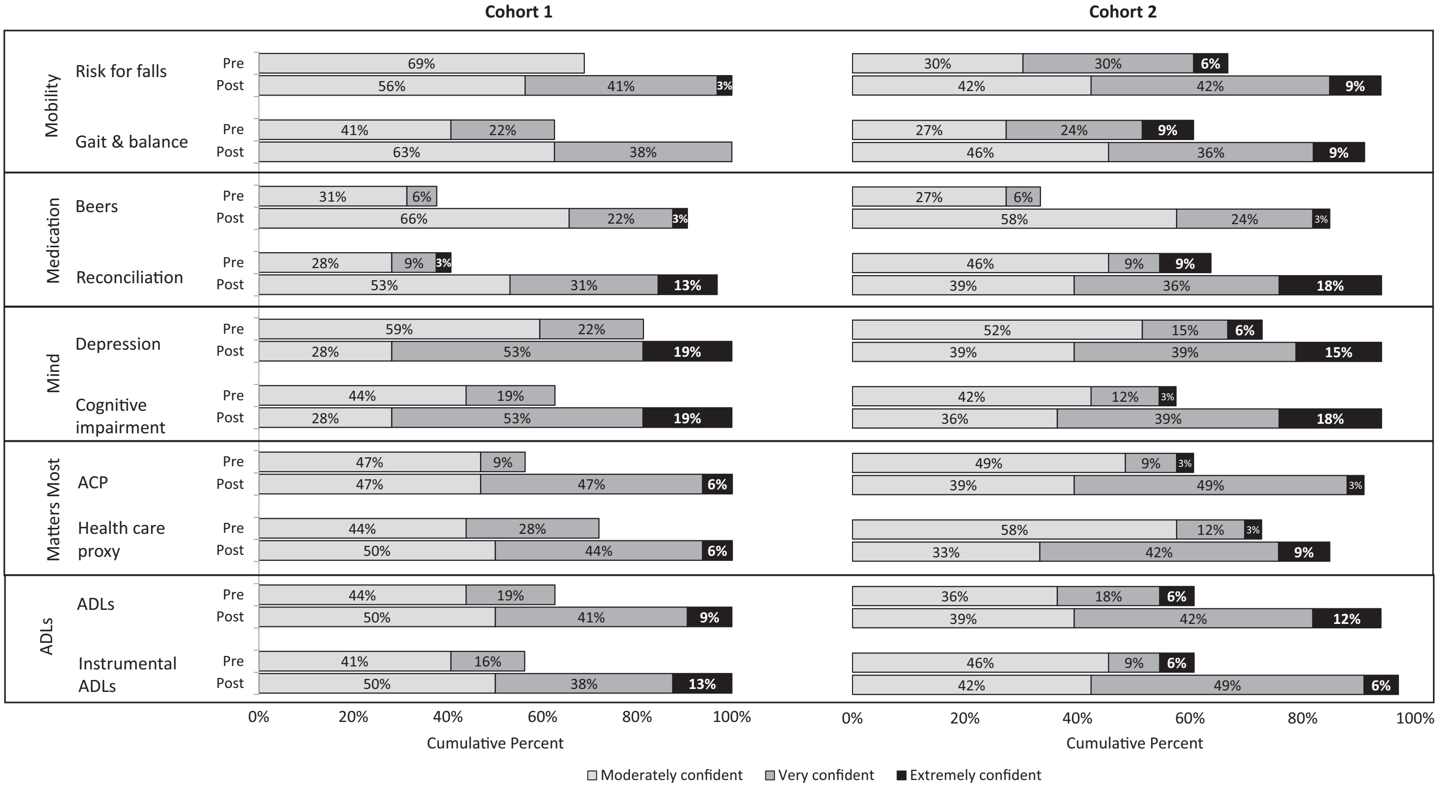

Figure. Student confidence in knowledge and skills before and after the session. Abbreviations: ACP, advance care planning; ADLs, activities of daily living; Beers, Beers criteria for potentially inappropriate medication use in older adults.

take-home points fell into the categories of general 4Ms (n = 20), mind/memory (n = 4), medications (n = 17), mobility (n = 11), matters most (n = 7), general geriatrics concepts (n = 3), and general communication skills (n = 3). Medication and general communication skills were more frequently highlighted by cohort 1 students, while the general 4Ms and mobility and matters most categories were more frequently highlighted by cohort 2.

Session Evaluation Survey

Twenty-five percent of students were strongly encouraged to complete a session evaluation at the conclusion of the session. In cohort 1, 19 students (76% response rate) and, in cohort 2, 24 students (97% response rate) completed the session evaluation. Table 2 shows the distribution of responses for each survey item by group. There were no significant differences comparing the two groups (all $ps > .05$).

Table 2. Session Evaluation Data

	Cohort 1 (N = 19)			Cohort 2 (N = 24)			
Item	SD/D	N	SA/A	SD/D	N	SA/A	Mann-Whitney U Test[a]
1. The learning objectives and prework prepared me for the session.	0%	21%	79%	0%	42%	58%	U = 198, p = .43
2. The faculty member fostered active learning.	5%	5%	89%	8%	21%	71%	U = 212, p = .67
3. The session appropriately balanced individual facts and conceptual knowledge as related to the goals of the session.	5%	0%	95%	13%	21%	67%	U = 183, p = .23
4. The instructor communicated material at a level that was appropriate for the learners.	5%	0%	95%	4%	17%	79%	U = 209, p = .61
5. The instructor was well organized, delivering content in a logical progression.	5%	0%	95%	4%	21%	75%	U = 195, p = .38
6. The tone or atmosphere of the session enabled learners to comfortably identify and address their concerns and/or limitations.	5%	11%	84%	8%	13%	79%	U = 215, p = .74
7. The session appropriately complemented/enhanced my learning relative to this week's theme.	5%	5%	89%	17%	17%	67%	U =213, p = .69

Abbreviations: SD/D, strongly disagree/disagree; N, neither agree nor disagree; SA/A strongly agree/agree.
[a]The Mann-Whitney U test was performed on the full 5-point Likert scale.

Copyright © 2022 Goldberg et al. This is an open-access publication distributed under the terms of the Creative Commons Attribution license.

MedEdPORTAL® The AAMC Journal of Teaching and Learning Resources

OSCE Performance

We assessed the impact of the curriculum on student performance of a geriatric assessment with an SP. For this OSCE, data from all students were available for analysis (cohort 1, $N = 100$; cohort 2, $N = 99$). Table 3 presents the number of participants who received full (or partial) credit for each checklist item. In cohort 2, significantly more students used a cognitive screening tool to assess the SP's cognitive function ($\chi^2 = 40.50$, $p < .001$) and asked about advance care planning ($\chi^2 = 48.03$, $p < .001$) than in cohort 1. There was a significant association between student group and whether the student asked about medications and how the SP was taking them ($\chi^2 = 24.68$, $p < .001$). A post hoc chi-square test on that item revealed that cohort 2 students were more likely to receive full credit (adjusted $p = .02$) or no credit (adjusted $p = .01$) and less likely to have partial credit (adjusted $p < .001$) than students in cohort 1.

Discussion

To address the need for all physicians to be trained in the principles of the geriatric assessment, we designed an interactive, skills-based curriculum for MS2s. Our session was well received by students. Students self-assessed improved confidence in their knowledge and skills performing components of the geriatric assessment after the session. After completing this curriculum virtually, students successfully performed the components of a basic geriatric assessment on an OSCE conducted either virtually (cohort 1) or in person (cohort 2). Updates to the curriculum after cohort 1 included active practice role-playing skills with debrief, as well as addition of curricular content pertaining to matters most. We believe the active engagement in the role-play component for cohort 2, in place of the passive role-modeling of skills for cohort 1, explains students' improved performance on the OSCE. The students in cohort 2 may also have performed better as their OSCE was conducted in person rather than virtually. The addition of advance care planning content for cohort 2 likely accounted for more students in this cohort identifying matters most as a take-home point from the session.

The session was scheduled during the final weeks of the final MS2 course, just prior to the study period for the Step 1 Exam. This timing may have impacted students' response rate to the surveys. We would have preferred a higher response rate to our pre-post surveys and must acknowledge that the students who completed both may have been more motivated and engaged; however, we are reassured by the similarities between cohorts. Based on student feedback, future iterations will include opportunities for role-modeling of the matters most portion of the encounter to review and consolidate this content. Four students in cohort 1, but no students in cohort 2, identified communication skills as a main takeaway. This may be explained by the fact that cohort 1 had role-modeling of the scenarios during the large group. An additional consideration for curricular improvement would therefore be integration of role-modeling of all stages of the geriatric assessment either during the session or as prework. This role-modeling may better prepare students for the communication skills necessary for real-time role-playing of scenarios during the session.

The major challenge we faced in the implementation of the curriculum was the COVID pandemic, which forced us to pivot to a virtual platform with minimal turnaround time. This pivot required adaptation of our curricular plan for cohort 1, with replacement of the real-time application of skills through student role-play of cases by faculty role-modeling of the cases. Despite this shift, students performed well on the clinical skills assessment. As a result of this pivot, we now have data to support the adaptability and versatility of this curriculum. The virtual format limited faculty's availability during the in-session role-plays. Future iterations of this curriculum will

Table 3. Number (%) of Students Receiving Full or Partial Credit on Standardized Patient Checklist Items During the Clinical Skills Encounter

Item	Cohort 1 ($N = 100$)	Cohort 2 ($N = 99$)	Chi-Square Analysis
Did the student:			
Ask about your medications and how you are taking them?			$\chi^2 = 24.68$, $p < .001$
Full credit	64 (64%)	81 (82%)	
Partial credit	36 (36%)	10 (10%)	
No credit	0 (0%)	8 (8%)	
Use a cognitive screening tool to assess your cognitive function?	40 (40%)	83 (84%)	$\chi^2 = 40.50$, $p < .001$
Ask you whether you ever completed a health care proxy or any other questions pertaining to advanced care planning?	14 (14%)	61 (62%)	$\chi^2 = 48.03$, $p < .001$
Ask any question to assess your fall risk (vision, rugs, stairs, prior fall history, use of assistive devices, balance, or coordination)?	84 (84%)	86 (87%)	$\chi^2 = 0.33$, $p = .57$
Elicit your ability to perform activities of daily living?	92 (92%)	96 (97%)	$\chi^2 = 2.35$, $p = .13$

Copyright © 2022 Goldberg et al. This is an open-access publication distributed under the terms of the Creative Commons Attribution license.

take place in person, in a large classroom, which will allow for geriatrics faculty to be present and circulating during the role-play portions to provide additional real-time feedback. Despite the provision of scripts for students to play the role of the patient in each scenario, we acknowledge that students are limited in their ability to authentically portray older adult patients. For centers with adequate resources, we would encourage the consideration of the use of SPs for the in-session role-plays.

Because the ultimate goal of this curriculum is to prepare learners for the care of the growing older adult population, we will consider further assessment of student skills later in the curriculum to assess for retention and application of these skills in the workplace. Additional curricular time is still needed for the fifth M of multicomplexity, including hazards of hospitalization, which is more applicable to students with experience in the inpatient setting.

"The 4Ms Approach to the Care of the Older Adult" curriculum is easily reproducible at any institution with the tools provided in this publication. As noted in the faculty guide (Appendix B), the curriculum will require minor edits, including identifying the cognitive screening tools and advance care planning documentation and resources pertinent to the local practice environment. The curriculum, as well as the supporting SP case, can be delivered effectively both virtually and in-person. The curriculum meets both the demographic imperative to prepare all medical students to care for older adults and the need to create adaptable pedagogies easily deliverable both in person and on virtual platforms.

Appendices

A. The 4Ms Approach.pptx
B. Faculty Guide.docx
C. Student A Handout.docx
D. Student B Handout.docx
E. Student C Handout.docx
F. Pre- and Postsession Student Surveys.docx
G. Large-Group Session Evaluation Form.docx
H. Geriatrics SP Case.docx
I. Geriatrics SP Checklist.docx

All appendices are peer reviewed as integral parts of the Original Publication.

Gabrielle R. Goldberg, MD: Assistant Professor and Director, Clinical Skills, Department of Science Education, Donald and Barbara Zucker School of Medicine at Hofstra/Northwell; ORCID: https://orcid.org/0000-0001-7837-7041

Gabriela Solis, MD: Assistant Professor, Department of Geriatrics and Palliative Care, Donald and Barbara Zucker School of Medicine at Hofstra/Northwell

Janice T. John, DO, MS, MPH: Assistant Professor, Department of Pediatrics, and Assistant Dean for Integrated Medical Education, Albert Einstein College of Medicine

Doreen M. Olvet, PhD: Associate Professor and Medical Education Research Project Manager, Department of Science Education, Donald and Barbara Zucker School of Medicine at Hofstra/Northwell

Kimberly A. Kranz, MD: Associate Professor, Department of Internal Medicine and Geriatrics, Renaissance School of Medicine at Stony Brook University

Disclosures:
None to report.

Funding/Support:
None to report.

Ethical Approval:
The Donald and Barbara Zucker School of Medicine Institutional Review Board deemed further review of this project not necessary.

References

1. Older people projected to outnumber children for first time in U.S. history. United States Census Bureau. 2018. Updated October 8, 2021. Accessed December 7, 2021. https://www.census.gov/newsroom/press-releases/2018/cb18-41-population-projections.html
2. Share of old age population (65 years and older) in the total U.S. population from 1950 to 2050. Statista. 2021. Accessed December 7, 2021. https://www.statista.com/statistics/457822/share-of-old-age-population-in-the-total-us-population/
3. Petriceks AH, Olivas JC, Srivastava S. Trends in geriatrics graduate medical education programs and positions, 2001 to 2018. *Gerontol Geriatr Med.* 2018;4:2333721418777659. https://doi.org/10.1177/2333721418777659
4. Blachman NL, Blaum CS, Zabar S. Reasons geriatrics fellows choose geriatrics as a career, and implications for workforce recruitment. *Gerontol Geriatr Educ.* 2021;42(1):38-45. https://doi.org/10.1080/02701960.2019.1604341
5. Meiboom AA, de Vries H, Hertogh CMPM, Scheele F. Why medical students do not choose a career in geriatrics: a systematic review. *BMC Med Educ.* 2015;15:101. https://doi.org/10.1186/s12909-015-0384-4

Copyright © 2022 Goldberg et al. This is an open-access publication distributed under the terms of the Creative Commons Attribution license.

6. Leipzig RM, Granville L, Simpson D, Anderson MB, Sauvigné K, Soriano RP. Keeping granny safe on July 1: a consensus on minimum geriatrics competencies for graduating medical students. *Acad Med.* 2009;84(5):604-610. https://doi.org/10.1097/ACM.0b013e31819fab70
7. Denham AC. Clinical decision-making for older patients with multiple chronic conditions: a case-based exercise for medical students. *MedEdPORTAL.* 2016;12:10477. https://doi.org/10.15766/mep_2374-8265.10477
8. Denham AC, Winzelberg G. A fall in an older adult: a case-based exercise for medical students. *MedEdPORTAL.* 2013;9:9354. https://doi.org/10.15766/mep_2374-8265.9354
9. Denham AC, Winzelberg G. Failure to thrive in an older adult: a case-based exercise for medical students. *MedEdPORTAL.* 2013;9:9347. https://doi.org/10.15766/mep_2374-8265.9347
10. Winzelberg GS, Denham AC. Altered mental status in an older adult: a case-based exercise for medical students. *MedEdPORTAL.* 2013;9:9319. https://doi.org/10.15766/mep_2374-8265.9319
11. Gentile M, Overbeck K, Basehore P, Chopra A, Alburger K. Margaret Pelli: a geriatric standardized patient case. *MedEdPORTAL.* 2013;9:9500. https://doi.org/10.15766/mep_2374-8265.9500
12. Basehore P, Chopra A, Overbeck K. Henry Corbett: a geriatric standardized patient case. *MedEdPORTAL.* 2013;9:9649. https://doi.org/10.15766/mep_2374-8265.9649
13. Overbeck K, Basehore P, Chopra A. Objective structured clinical examination case: Gina Babkins. *MedEdPORTAL.* 2013;9:9455. https://doi.org/10.15766/mep_2374-8265.9455
14. van Zuilen M, Gonzalez J, Aguilar E, Palacios JC, Mintzer M. Geriatrics medication history objective structured clinical exam. *MedEdPORTAL.* 2011;7:8274. https://doi.org/10.15766/mep_2374-8265.8274
15. Jirau-Rosaly W, Brown SP, Wood EA, Rockich-Winston N. Integrating an interprofessional geriatric active learning workshop into undergraduate medical curriculum. *J Med Educ Curric Dev.* 2020;7:2382120520923680. https://doi.org/10.1177/2382120520923680
16. Tinetti M, Huang A, Molnar F. The Geriatrics 5M's: a new way of communicating what we do. *J Am Geriatr Soc.* 2017;65(9):2115. https://doi.org/10.1111/jgs.14979
17. Geriatrics competencies for medical students. Association of Directors of Geriatrics Academic Programs. Accessed March 30, 2022. https://adgap.americangeriatrics.org/education-training/competencies/geriatrics-competencies-medical-students
18. Miller RK, Young ME, Sehgal M. Strategies that can integrate meaningful geriatrics content into medical school curricula. *Acad Med.* 2021;96(12):1626-1627. https://doi.org/10.1097/ACM.0000000000004158
19. Systems Age-Friendly Health. Institute for Healthcare Improvement. Accessed March 30, 2022. http://www.ihi.org/Engage/Initiatives/Age-Friendly-Health-Systems/Pages/default.aspx
20. Kelley AS, Back AL, Arnold RM, et al. Geritalk: communication skills training for geriatric and palliative medicine fellows. *J Am Geriatr Soc.* 2012;60(2):332-327. https://doi.org/10.1111/j.1532-5415.2011.03787.x
21. Taylor DCM, Hamdy H. Adult learning theories: implications for learning and teaching in medical education: AMEE Guide no. 83. *Med Teach.* 2013;35(11):e1561-e1572. https://doi.org/10.3109/0142159X.2013.828153
22. Yardley S, Teunissen PW, Dornan T. Experiential learning: AMEE Guide no. 63. *Med Teach.* 2012;34(2):e102-e115. https://doi.org/10.3109/0142159X.2012.650741
23. Carlson C, Merel SE, Yukawa M. Geriatric syndromes and geriatric assessment for the generalist. *Med Clin North Am.* 2015;99(2):263-279. https://doi.org/10.1016/j.mcna.2014.11.003
24. Schön DA. *The Reflective Practitioner: How Professionals Think in Action.* Basic Books; 1983.
25. Folstein MF, Folstein SE, McHugh PR. “Mini-Mental State”: a practical method for grading the cognitive state of patients for the clinician. *J Psychiatr Res.* 1975;12(3):189-198. https://doi.org/10.1016/0022-3956(75)90026-6
26. Phillips SC, Hawley CE, Triantafylidis LK, Schwartz AW. Geriatrics 5Ms for primary care workshop. *MedEdPORTAL.* 2019;15:10814. https://doi.org/10.15766/mep_2374-8265.10814
27. Beasley TM, Schumacker RE. Multiple regression approach to analyzing contingency tables: post hoc and planned comparison procedures. *J Exp Educ.* 1995;64(1):79-93. https://doi.org/10.1080/00220973.1995.9943797

Received: January 9, 2022
Accepted: May 13, 2022
Published: June 28, 2022

Copyright © 2022 Goldberg et al. This is an open-access publication distributed under the terms of the Creative Commons Attribution license.
